# Huaier Inhibits Gastric Cancer Growth and Hepatic Metastasis by Reducing Syntenin Expression and STAT3 Phosphorylation

**DOI:** 10.1155/2022/6065516

**Published:** 2022-06-15

**Authors:** Yunfu Shi, Li Yuan, Jingli Xu, Handong Xu, Lijing Wang, Ling Huang, Zhiyuan Xu, Xiangdong Cheng

**Affiliations:** ^1^Tongde Hospital Affiliated to Zhejiang Chinese Medical University (Tongde Hospital of Zhejiang Province), Hangzhou 310012, China; ^2^The First Clinical Medical College of Zhejiang Chinese Medical University, Hangzhou, Zhejiang 310053, China; ^3^The Cancer Hospital of the University of Chinese Academy of Sciences (Zhejiang Cancer Hospital), Institutes of Basic Medicine and Cancer (IBMC), Chinese Academy of Sciences, Hangzhou 310022, China; ^4^Zhejiang Provincial Research Center for Upper Gastrointestinal Tract Cancer, Zhejiang Cancer Hospital, Hangzhou 310022, China; ^5^Key Laboratory of Prevention, Diagnosis and Therapy of Upper Gastrointestinal Cancer of Zhejiang Province, Hangzhou 310022, China

## Abstract

Gastric cancer (GC) is a common malignant tumor worldwide and poses a serious threat to human health. As a traditional Chinese medicine, Huaier (*Trametes robiniophila* Murr.) has been used in the clinical treatment of GC. However, the mechanism underlying the anticancer effect of Huaier remains poorly understood. In this study, we used *in vivo* imaging technology to determine the anticancer effect of the Huaier n-butanol extract (HBE) on orthotopic and hepatic metastasis of GC mouse models. We found that HBE suppressed tumor growth and metastasis without causing apparent host toxicity. Proteomic analysis of GC cells before and after HBE intervention revealed syntenin to be one of the most significantly downregulated proteins after HBE intervention. We further demonstrated that HBE suppressed the growth and metastasis of GC by reducing the expression of syntenin and the phosphorylation of STAT3 at Y705 and reversing the epithelial-mesenchymal transition (EMT). In addition, we confirmed that syntenin was highly expressed in GC tissue and correlated with metastasis and poor prognosis. In conclusion, our results suggest that Huaier, a clinically used anticancer drug, may inhibit the growth and liver metastasis of GC by inhibiting the syntenin/STAT3 signaling pathway and reversing EMT.

## 1. Introduction

Gastric cancer (GC) has the fifth highest incidence and is the fourth leading cause of death worldwide [1]. According to the latest report from the International Agency for Research on Cancer (IARC), approximately 1.09 million people were diagnosed with GC and about 0.77 million deaths were attributed to GC in 2020 [1]. East Asia has a high incidence of this disease, and China accounts for approximately half of the new cases and deaths in the world [2]. The prognosis of GC is closely related to its clinical stage. The prognosis of patients with localized GC is good, with a 5-year survival rate of more than 60%, while that of patients with advanced GC is less than 30% [3]. The liver is one of the most common organs for gastric cancer metastasis [4]. In recent years, with the application of targeted therapy and immunotherapy, the mortality rates of patients with malignant tumors such as breast cancer and non-small-cell lung cancer (NSCLC) have decreased, and there is a renewed hope for conquering cancer. However, these new treatments have not significantly improved the survival rate of patients with GC [5]. Therefore, safe and effective drugs are urgently needed for the treatment of GC. At present, researchers are increasingly interested in screening traditional Chinese medicine (TCM) for anticancer activity.

Huaier (*Trametes robiniophila* Murr.) has been used in China for more than one thousand years. At present, Huaier granules are widely used in the clinical treatment of cancers including GC, and the effect was confirmed by clinical trials. Chen et al. revealed that Huaier granules as an adjuvant after radical hepatectomy prolonged recurrence-free survival and reduced the extrahepatic recurrence rate in patients [6]. Qi et al. confirmed that Huaier granules combined with tegafur-gimeracil-oteracil potassium could improve the prognosis of patients with GC, as the disease-free survival rate and the overall survival rate were improved [7]. However, the anticancer components and molecular mechanism of Huaier remains poorly understood.

In this study, we sought to determine the anticancer effect of the Huaier n-butanol extract (HBE) on orthotopic and hepatic metastasis GC mouse models. We found that HBE works by downregulating the syntenin/STAT3 signaling pathway and confirm that high syntenin expression is correlated with metastasis and poor prognosis of GC. These findings will provide a potential therapeutic strategy for the treatment of GC.

## 2. Materials and Methods

### 2.1. Cell Lines, Reagents, and Antibodies

The human gastric epithelial cell line GES-1 and gastric cancer MGC803, MKN74, AZ-521, and MKN28 cell lines were obtained from the Institute of Cancer, Zhejiang Chinese Medical University (Hangzhou, China). Cells were cultured at 37°C with 5% CO_2_ in the recommended medium supplemented with 10% FBS (Gibco, Grand Island, USA), 100 U/ml penicillin (Kino Co., Ltd, Hangzhou, China), and 100 *μ*g/ml streptomycin (Kino Co., Ltd, Hangzhou, China). The antibodies targeting syntenin (cat. no.: ab133267; lot: GR3375272-3), E-cadherin (cat. no.: ab76055; lot: 3360021-1), N-cadherin (cat. no.: ab76011; lot: GR3245174-11), and vimentin (cat. no.: ab92547; lot: GR3258719-22) were purchased from Abcam (Cambridge, UK), while antibodies against Stat3 (cat. no.: 30835S, lot: 1) and P-Stat3 (Y705) (cat. no.: 9145S; lot: 43) were purchased from Cell Signaling Technology (Boston, USA). Anti-GAPDH (cat. no.: 60004-1-Ig; lot: 10017731) was purchased from Proteintech (Chicago, IL, USA).

### 2.2. Preparation of the Huaier n-Butanol Extract

The HBE was prepared as described previously [8]. Potential compounds of HBE identified by LC-MS analysis (Figures S1–S8) are described in Table S1, which were used as quality control standards.

### 2.3. Orthotopic GC Mouse Models

Orthotopic GC mouse models were generated as previously reported [9]. A total of 5 × 10^6^ MGC803-Luc or MKN74-Luc cells were suspended in 100 *μ*l of PBS and subcutaneously injected into the bilateral flanks of nude mice. When subcutaneous tumors grew to 0.5–1 cm, mice were killed and tumors were resected under sterile conditions. Tumor tissues were minced into 1-2 mm^3^ fragments with scissors. Under general anesthesia, a 10–15 mm midline incision was made in the upper abdomen, and the stomach was carefully exposed. The serosal membrane in the middle of the greater curvature of the stomach was mechanically injured with a scalpel. A piece of tumor tissue was then fixed onto the injured site of the serosal surface with medical glue. The stomach was then sent back to the abdominal cavity, and the abdominal wall and skin were sutured.

One week later, the fluorescence intensity of the stomach was determined by *in vivo* imaging. Positive fluorescence in the stomach indicates the successful establishment of the orthotopic GC model. These mice were divided into two or three groups according to the fluorescence intensity. The MGC803 orthotopic model mice were treated with HBE at doses of 0 and 100 mg/kg/day daily for 4 weeks. For the MKN74 orthotopic model, mice were treated similarly with doses of 0, 50, and 100 mg/kg/day for 30 days.

### 2.4. Mouse Model of GC Hepatic Metastasis

A mouse model of GC hepatic metastasis was established as previously reported [10]. Under general anesthesia, incisions of 8–10 mm were made in the left upper abdomen, and the spleen was carefully exposed. A total of 5 × 10^6^ MGC803-Luc cells were suspended in 200 *μ*l of PBS and slowly injected into the spleen. The puncture site on the spleen was suppressed using cotton swabs for 3 min. To prevent tumor growth in the spleen, which would affect the fluorescence intensity of liver metastasis, the spleen was removed 10 min later [11]. Finally, the abdominal wall and skin were stitched with 5-0 sutures.

*In vivo* imaging was performed on the day after the operation. The mice were then divided into three groups according to the fluorescence intensity and gavaged with HBE at doses of 0, 50, and 100 mg/kg/day for 10 days.

### 2.5. *In Vivo* Imaging

The volumes of orthotopic tumors and hepatic metastasis were measured noninvasively using the *in vivo* imaging system (IVIS) Lumina LT (Caliper Life Sciences, USA). D-Luciferin sodium salt (150 mg/kg) was injected intraperitoneally, and then mice were anesthetized. Luciferase activity was measured using the IVIS at 10–15 min after the injection of D-Luciferin sodium salt. We used Living Image Ver. 4.3 (Caliper Life Sciences, USA) software to acquire the data.

### 2.6. Hematoxylin and Eosin Staining and Immunohistochemistry

Tumors and important organs were removed from the mice, fixed in 4% paraformaldehyde, and embedded in paraffin. The tumor and organ sections (4-5 *μ*m thick) were then deparaffinized, rehydrated, and washed. Sections were stained with H&E. Immunohistochemistry (IHC) staining of target proteins was performed using biotinylated antibodies and HRP/DAB detection.

### 2.7. Cell Viability Assay

The cell viability was performed using the cck-8 assay as previously reported [12]. In brief, MGC803, MKN74, AZ-521, MNK28, and GES-1 cells were cultured overnight in 96-well plates (2000–6000 cells/well), and HBE (0, 20, 40, 60, 80, 100, 120, 160, or 200 *μ*g/mL) was then added to the coculture for 24, 48, and 72 h. Finally, CCK-8 solution was added, absorbance was measured, and the cell viability and IC_50_ values were calculated.

### 2.8. Protein Extraction and Mass Spectrometry

Lysis buffer was added to the protein samples and sonicated three times on ice using a high-intensity ultrasound processor. The lysate was centrifuged at 12,000 g at 4°C for 10 min, and the supernatant was collected. Finally, the protein concentration was determined using the BCA kit.

Proteins were digested by the filter-aided sample preparation (FASP) method [13], and peptides were analyzed by Q-Exactive mass spectrometry (MS) as previously reported [14]. Raw MS files were analyzed using MaxQuant software [15].

### 2.9. Western Blotting Assay

The quantified protein samples were added to the gel wells, separated by SDS-PAGE, and transferred onto PVDF membranes (Millipore, MA, USA). The membranes were incubated with specific primary antibodies and corresponding secondary antibodies. Finally, the bands were visualized by enhanced chemiluminescence (ECL). Intensity was measured by Image Lab 5.2 software.

### 2.10. Lentiviral Construction and Infection

Lentiviral expression vectors encoding syntenin and shSyntenin as well as packaging vectors were transfected into 293T cells. Two days after transfection, the virus particles were collected and filtered using a 0.45 *μ*m filter. GC cells were transfected with lentivirus, and the transfected cells were collected after 72 h. Overexpression and knockdown cell lines were selected with puromycin. Transfection efficiency was determined by western blotting.

### 2.11. Human GC Samples and Tissue Microarray Construction

In total, 135 pairs of tumor tissues and corresponding adjacent noncancerous tissues from GC patients were selected between January 2013 and December 2017 in Zhejiang Cancer Hospital. These patients had no history of chemotherapy or radiotherapy treatment prior to specimen collection. Tissue microarrays (TMAs) were constructed as described previously [8].

### 2.12. Tissue Microarrays, Immunohistochemistry Staining, and Stratification of Syntenin Expression in GC

Immunohistochemistry staining of serial tissue microarrays (TMAs) was carried out as described previously, and brown cells (HRP/DAB stained) were considered positive. We assessed the staining intensity using the following scoring system: 0 (negative), 1 (weak), 2 (moderate), and 3 (strong). The staining area was scored as 0 (none), 1 (1–25%), 2 (26–50%), 3 (51–75%), or 4 (76–100%). The staining intensity score and staining area score were then multiplied to produce a final score. Scores of 0 to 6 were regarded as low expression, and scores of 7 to 12 were considered high expression.

### 2.13. Statistical Analysis

All statistical analyses were performed using SPSS 23.0 software (SPSS Inc., Chicago, IL, USA). Normally distributed measurement data are presented as the mean ± SEM, and nonnormally distributed measurement data are presented as the median (interquartile range, IQR). Parametric tests (Student's *t* test or one-way ANOVA) or nonparametric tests were used, depending on the type of data distribution and homogeneity of variance. Count data are presented by the rate or composition ratio, using the chi-square test and Fisher's exact test. Survival curves were estimated using the Kaplan–Meier method, and univariate and multivariate analyses were performed using a Cox proportional hazard model. *p* < 0.05 was considered statistically significant.

## 3. Results

### 3.1. HBE Suppresses GC Growth and Metastasis *In Vivo*

**Figure 1:**
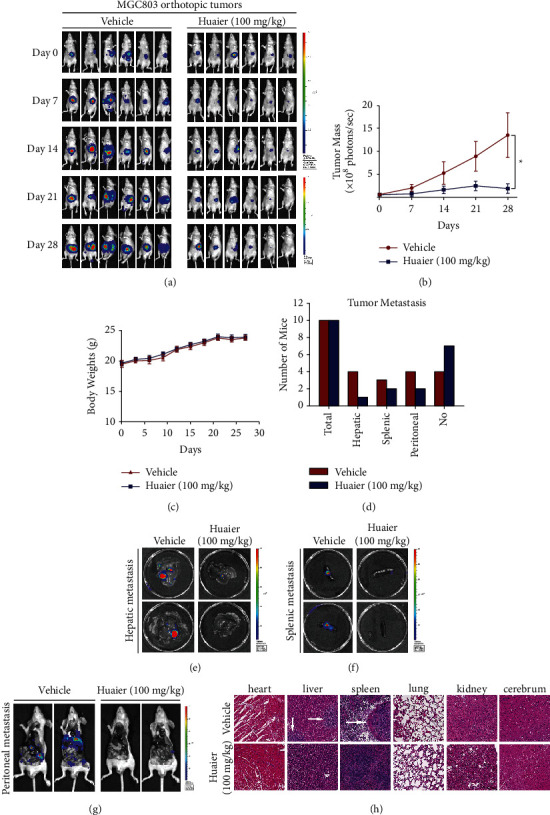
HBE suppresses the growth and metastasis of MGC803 orthotopic tumors without causing notable host toxicity. (a) Representative images of in vivo imaging. (b) The growth curves of tumor (^*∗*^*p* < 0.05). (c) Average body weight of mice bearing tumors. (d) Numbers of mice with liver, spleen, and peritoneal metastasis. (e) Representative images of hepatic metastasis. (f) Representative images of splenic metastasis. (g) Representative images of peritoneal metastasis. (h) Representative H&E staining images of important organs. Data are presented as the mean ± SEM.

**Figure 2:**
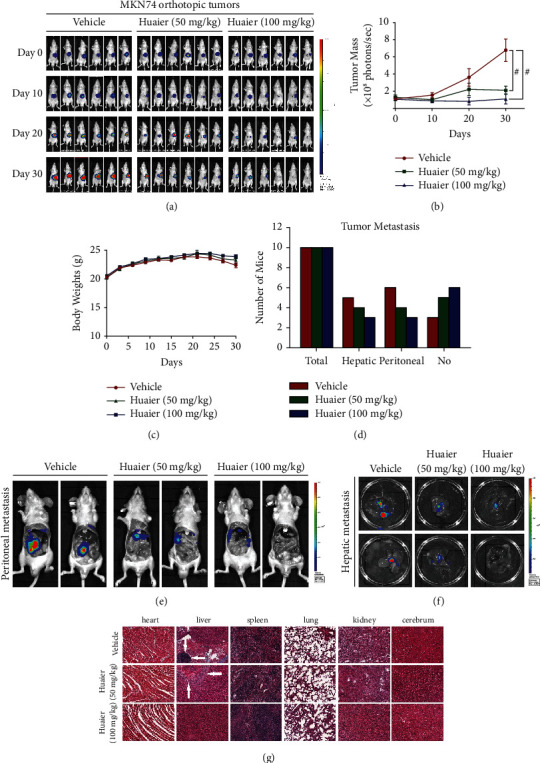
HBE suppresses the growth and metastasis of MNK74 orthotopic tumors without causing notable host toxicity. (a) Representative in vivo images. (b) The growth curves of tumor (^#^*p* < 0.01). (c) Average body weight of mice bearing tumors. (d) Numbers of mice with liver and peritoneal metastasis. (e) Representative images of peritoneal metastasis. (f) Representative images of hepatic metastasis. (g) Representative H&E staining images of important organs. Data are presented as the mean ± SEM.

We examined the anticancer effect of HBE in two orthotopic mouse models of GC: MGC803 and MKN74 orthotopic models. The results show that HBE at a dose of 100 mg/kg/day significantly inhibited the growth of MGC803 orthotopic tumors (Figures 1(a) and 1(b)). We also observed that HBE inhibited metastasis to the liver, peritoneum, and spleen (Figures 1(d)–1(h)). Similarly, HBE at doses of 50 and 100 mg/kg/day had anticancer effects in the MKN74 orthotopic tumor model (Figures 2(a) and 2(b)). However, there was no significant difference between the 50 and 100 mg/kg/day dose groups. At the same time, HBE inhibited metastasis to the liver and peritoneum (Figures 2(d)–2(g)) in the MKN74 orthotopic tumor model, but we did not observe splenic metastasis in this model. Importantly, HBE treatment had no effect on the body weight of the mice (Figures 1(c) and 2(c)), and no significant tissue damage was observed in the H&E staining of the heart, liver, spleen, lung, kidney, and cerebrum (Figures 1(h) and 2(g)). Moreover, both routine blood tests and liver and kidney function tests showed no abnormalities (Figure S9). These results suggested that HBE did not cause significant host toxicity.

### 3.2. HBE Inhibits the Formation of Hepatic Metastases *In Vivo*

**Figure 3:**
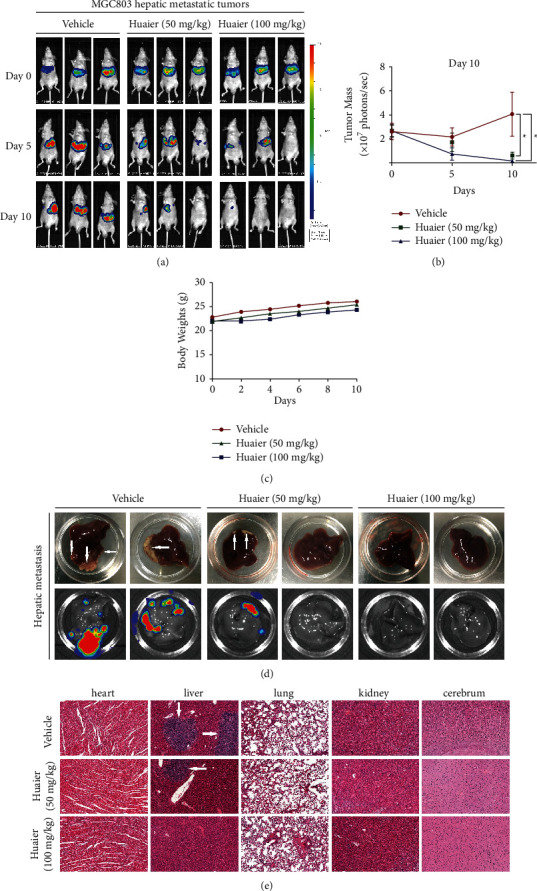
HBE inhibits the formation of hepatic metastasis. (a) Representative in vivo images. (b) The growth curves of tumor (^*∗*^*p* < 0.05). (c) Average body weights of mice bearing tumors. (d) Representative images of liver metastasis. (e) Representative H&E staining images of important organs. Data are presented as the mean ± SEM.

We further assessed the antimetastatic effect of HBE in a mouse model of GC hepatic metastasis. As shown in Figures 3(a), 3(b), and 3(d), HBE at doses of 50 and 100 mg/kg/day reduced liver metastasis in the MGC803 hepatic metastasis model. Moreover, H&E staining of the liver indicated that metastasis in the vehicle group was more obvious than that in the two treatment groups (Figure 3(e)). Similarly, HBE treatment had no effect on the body weight of the mice (Figure 3(c)), and H&E staining of the heart, liver, lung, kidney, and cerebrum showed no obvious abnormalities, indicating that HBE had no apparent host toxicity (Figure 3(e)).

### 3.3. HBE May Target Syntenin to Exert Its Anticancer Effect

**Figure 4:**
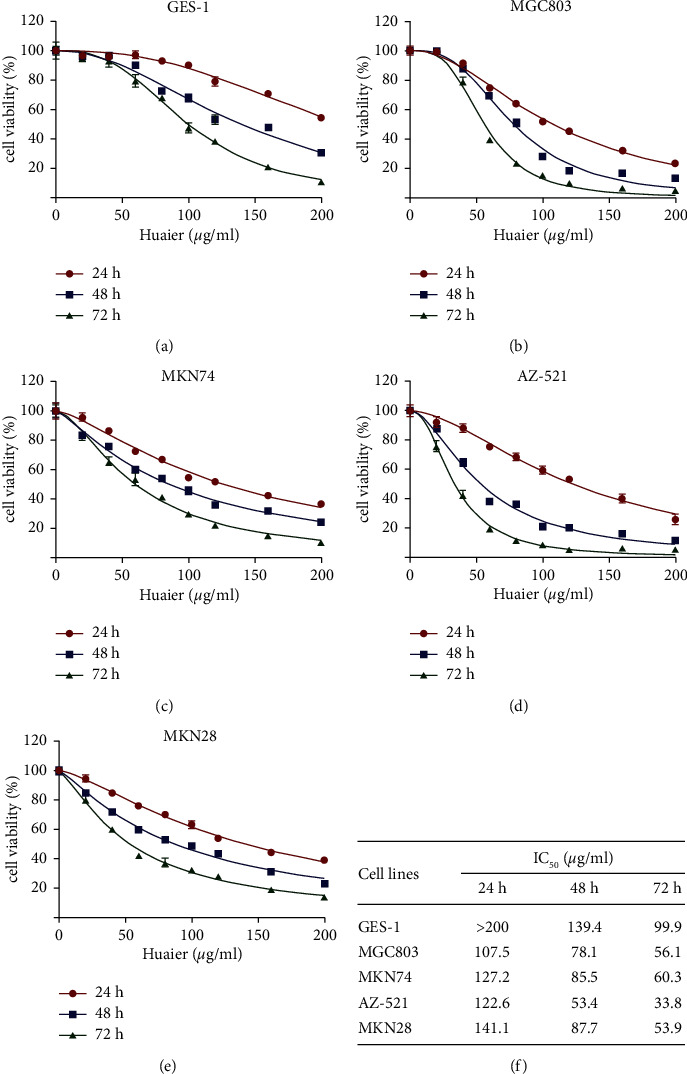
HBE inhibits the growth of GC cells in vitro. (a) Viability curve of GES-1 cells. (b) Viability curve of MGC803 cells. (c) Viability curve of MNK74 cells. (d) Viability curve of AZ-521 cells. (e) Viability curve of MNK28 cells. (f) IC_50_ value of each cell line. Data are presented as the mean ± SEM.

To explore the anticancer mechanism of HBE, we first carried out a CCK-8 experiment. HBE inhibited the growth of four human GC (MGC803, MKN74, AZ-521, and MKN28) cell lines, with IC_50_ values ranging from 107.5 to 141.1 *μ*g/mL for 24 h, 53.4 to 87.7 *μ*g/mL for 48 h, and 33.8 to 60.3 *μ*g/mL for 72 h (Figures 4(a)–4(f)). However, the IC_50_ value in the noncancerous GES-1 cells was higher than that in GC cell lines, indicating that HBE had selective cytotoxicity in GC cells.

**Figure 5:**
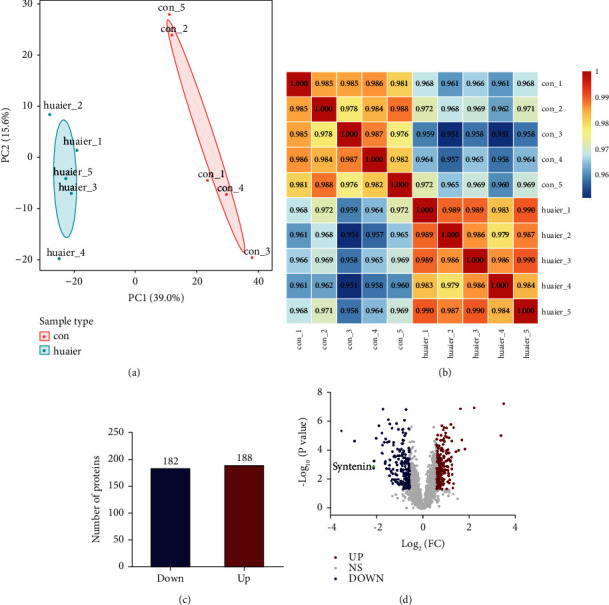
The differentially expressed proteins before and after HBE intervention were identified by proteomic analysis. (a) Representative images of principal component analysis. (b) Representative images of Pearson's correlation coefficient analysis. (c) Number of differentially expressed proteins. (d) Volcano map of the differentially expressed proteins.

We performed proteomic analysis of GC cells before and after HBE intervention to identify differentially expressed proteins. Principal component analysis (Figure 5(a)) and Pearson's correlation coefficient analysis (Figure 5(b)) revealed significant differences in protein expression between the control group and the HBE intervention group, and the samples in each group had good consistency. Using 1.5-fold as the threshold for differential expression and *p* value <0.05 as the threshold for significance, we found 188 proteins that were upregulated and 182 proteins that were downregulated after HBE treatment (Figures 5(c) and 5(d)). The top 10 differentially expressed proteins are shown in Table 1. Among these proteins, syntenin has been reported previously to be closely associated with invasion and metastasis in a variety of tumors [16–18] and may be the key target via which HBE exerts its anticancer effects.

### 3.4. HBE Inhibits the Syntenin/STAT3 Pathway and Reverses EMT Both *In Vivo* and *In Vitro*

**Figure 6:**
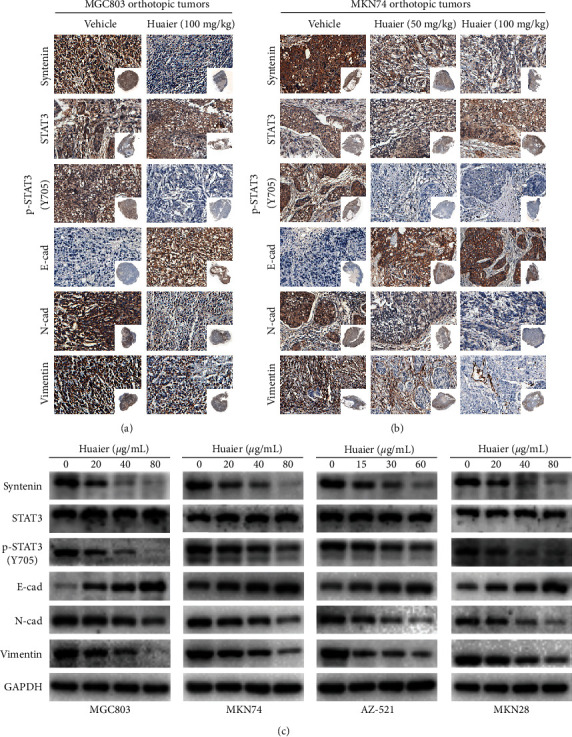
HBE inhibits the syntenin/STAT3 pathway and modulates the expression of EMT-related proteins both *in vivo* and *in vitro*. (a) Representative IHC images of MGC803 orthotopic tumors. (b) Representative IHC images of MKN74 orthotopic tumors. (c) Representative images of western blotting. E-cad: E-cadherin; N-cad: N-cadherin.

The signal transducer and activator of transcription 3 (STAT3) is a transcription factor that plays a pivotal role in cancer progression [19, 20]. Moreover, syntenin and STAT3 interactions have been observed in various cancers, and the syntenin/STAT3 pathway can promote tumor invasion and metastasis [21, 22]. As shown in Figures 6(a)–6(c), HBE suppressed the expression of syntenin both *in vivo* and *in vitro*. Moreover, HBE inhibited the activation of STAT3 by reducing the phosphorylation of STAT3 at Y705, suggesting that the syntenin/STAT3 pathway is targeted by HBE and may be involved in the anticancer mechanism of HBE.

Since EMT plays an important role in tumor metastasis, we further examined the expression levels of EMT-related markers to explore the mechanism of HBE against metastasis in GC. After treatment with HBE, the expression levels of N-cadherin and vimentin in four GC cell lines were reduced in a dose-dependent manner, while that of E-cadherin was increased (Figure 6(c)). The IHC analysis showed similar results *in vivo* (Figures 6(a) and 6(b)).

**Figure 7:**
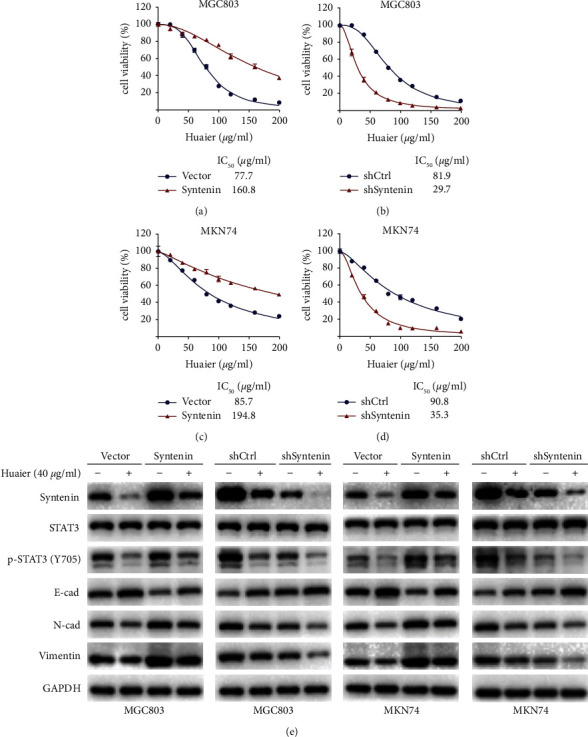
The syntenin/STAT3 pathway and EMT process are important for the anticancer effect of HBE. (a) Viability curve of MGC803 cells transfected syntenin and empty vector. (b) Viability curve of MGC803 cells transfected with shSyntenin and shCtrl. (c) Viability curve of MNK74 cells transfected with syntenin and empty vector. (d) Viability curve of MNK74 cells transfected with shSyntenin and shCtrl. (e) Representative images of western blotting. E-cad: E-cadherin; N-cad: N-cadherin. Data are presented as the mean ± SEM.

To determine whether HBE exerts its anticancer effect by targeting syntenin, MGC803 and MKN74 cells with stable overexpression or knockdown of syntenin were established and treated with HBE. The CCK-8 results showed that the IC_50_ value of syntenin-overexpressing cells was higher than that of control cells (Figures 7(a) and 7(c)), while the IC_50_ value of syntenin-knockdown cells was lower than that of control cells (Figures 7(b) and 7(d)). These results indicate that syntenin overexpression weakened the inhibitory effect of HBE, while syntenin knockdown strengthened the inhibitory effect of HBE.

As shown in Figure 7(e), HBE inhibited the activation of the syntenin/STAT3 pathway by reducing the expression of syntenin and phosphorylation of STAT3 at Y705 and modulated the expression of EMT-related proteins to inhibit the metastasis of GC. As expected, syntenin overexpression reversed the effect of HBE, while syntenin knockdown enhanced HBE regulation of the syntenin/STAT3 signaling pathway and EMT markers. Based on these results, syntenin is a key target for the metastasis of GC and is responsible for the anti-GC effect of HBE.

### 3.5. Syntenin Expression Is Markedly Upregulated in GC Tissues and Associated with Tumor Metastasis and Poor Prognosis

**Figure 8:**
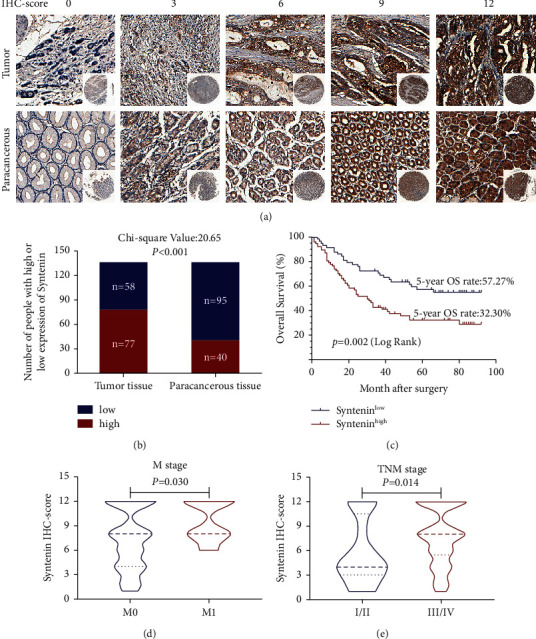
Syntenin is highly expressed in GC tissues and is associated with tumor metastasis and poor prognosis in GC. (a) Representative images of IHC staining of syntenin with different IHC scores in TMAs. (b) Differential expression of syntenin in GC and paracancerous tissues. (c) The survival curves of GC patients with different syntenin expression levels. (d) IHC staining intensities of syntenin in GC patients with different M stages. (e) IHC staining intensities of syntenin in GC patients with different TNM stages.

To further evaluate the role of syntenin in GC, the expression of syntenin was analyzed in tumor and paracancerous tissues of GC by IHC staining (Figure 8(a)). We found that 57.04% GC samples had strong syntenin staining in tumor tissues and the other 42.96% showed low syntenin expression in tumor tissues. In clear contrast, 29.63% GC samples exhibited strong syntenin intensity and 70.37% exhibited weak syntenin staining intensity in adjacent normal tissues (Figure 8(b)). These results indicated that syntenin was highly expressed in tumor tissues (*p* < 0.001).

We further compared the relationship between clinicopathological features and syntenin expression levels in gastric cancer patients and found that syntenin overexpression was associated with age, M stage, and TNM stage (Table 2, Figures 8(d) and 8(e)). Syntenin was found to be negatively correlated with the survival rate, shown by Kaplan–Meier plots (Figure 8(c)). The 5-year survival rate of GC patients with low syntenin expression was 57.27%, compared with high expression, which was only 32.30%. In addition, univariate and multivariate Cox regression analyses indicate that the syntenin expression is an independent prognostic factor (Table S2).

## 4. Discussion

TCM has been used for more than three thousand years in Asia. Because of their potential anticancer effect, low toxicity, low cost, and multiple targets, TCMs are increasingly being explored as anticancer drugs [23]. The TCM Huaier (*Trametes robiniophila* Murr.) has been used in the clinical treatment of various tumors, including gastric cancer [24–26]. At present, Huaier is clinically applied mainly as a water extract. However, we previously showed that the alcohol extract of Huaier, especially the n-butanol portion, had a better anticancer effect than the water extract [27]. In our present study, we found that HBE significantly inhibited the tumor growth and metastasis of orthotopic tumors without causing notable host toxicity. Moreover, we verified that HBE could inhibit the liver metastasis of GC in a hepatic metastasis model.

Syntenin was originally identified from metastatic human melanoma [28], also known as melanoma differentiation-associated gene-9 (MDA-9) and syndecan-binding protein (SDCBP). Syntenin plays an important regulatory role in multiple signaling pathways and is closely related to tumor metastasis [29–31]. Furthermore, many studies have reported that the expression of syntenin is elevated in tumor tissues. Kim et al. reported that syntenin expression was upregulated in SCLC cells and was more pronounced in patients with advanced stages of disease [32]. Bacolod et al. reported that syntenin expression is positively correlated with multiple cancers, including melanoma, prostate, and liver cancer, based on publicly available genomic datasets [33]. However, few studies have investigated the relationship between syntenin and GC, and only a small number were conducted at the cellular level. Koo et al. found that syntenin was highly expressed in metastatic human GC cell lines [18]. In our study, we found that syntenin was highly expressed in tumor tissues and was closely associated with age, M stage, and TNM stage. Multivariate Cox regression analysis indicated that the expression of syntenin was an independent prognostic factor in gastric cancer.

STAT3 pathway is closely related to tumor metastasis [34, 35]. Targeting the STAT3 protein in tumors has therapeutic promise, and several drugs targeting this protein have successfully entered the stage of clinical trials [36, 37]. Kegelman et al. showed that syntenin could phosphorylate STAT3, and the resulting activation enhanced the expression of the EMT-related markers MMP2 and MMP9 and promoted the migration of prostate cancer cells [31]. In our study, we found that HBE downregulated the expression of syntenin and the phosphorylation level of STAT3 at Y705, downregulated the levels of the EMT-related proteins N-cadherin and vimentin, and upregulated the level of E-cadherin. In addition, syntenin overexpression reversed the effect of HBE, while syntenin knockdown enhanced the HBE regulation of the syntenin/STAT3 signaling pathway and EMT process, indicating that syntenin is a key target for the metastasis of GC and responsible for the anti-GC metastasis effect of HBE.

In summary, our study shows that HBE can inhibit the growth and metastasis of GC, especially liver metastasis, by inhibiting the syntenin/STAT3 signaling pathway and reversing the EMT. This study provides a rational view of using HBE for the treatment of GC.

## Figures and Tables

**Table 1:** The top 10 differentially expressed proteins in HBE-treated MGC803 cells as identified by LFQ.

Regulation type	Accession no	Description	Gene name	Ratio
Down	O75306	NADH dehydrogenase iron-sulfur protein 2	NDUFS2	0.087
Down	P28331	NADH-ubiquinone oxidoreductase 75 kDa subunit	NDUFS1	0.129
Down	O00560	Syntenin	SDCBP	0.229
Down	P19404	NADH dehydrogenase flavoprotein 2	NDUFV2	0.229
Down	Q9C005	Protein dpy-30 homolog	DPY30	0.231
Up	P08243	Asparagine synthetase	ASNS	11.059
Up	Q13501	Sequestosome-1	SQSTM1	10.206
Up	P09601	Heme oxygenase 1	HMOX1	4.581
Up	Q12929	Epidermal growth factor receptor kinase substrate 8	EPS8	3.492
Up	Q06210	Glutamine-fructose-6-phosphate aminotransferase 1	GFPT1	3.182

**Table 2:** Correlations between the syntenin expression level and the clinicopathological features of GC patients.

Variables	Syntenin expression		Total	*χ* ^2^	*p* value
	High	Low			
*Age (years)*					
≤65	44	43	87	4.170	0.041^*∗*^
>65	33	15	48		
*Sex*					
Female	21	15	36	0.034	0.854
Male	56	43	99		
*Borrmann type*					
I + II	41	37	78	1.508	0.219
III + IV	36	21	57		
*Lauren type*					
Intestinal GC	41	30	71	2.319	0.314
Diffuse GC	27	16	43		
Mixed GC	9	12	21		
*Grade of differentiation*					
Well + moderate	42	31	73	0.016	0.899
Poor + not	35	27	62		
*T stage*					
T1	0	2	2	3.419	0.064
T2 + T3 + T4	77	56	133		
*N stage*					
N0	8	3	11	0.607	0.436
N1 + N2 + N3	69	55	124		
*M stage*					
M0	66	56	122	4.465	0.035^*∗*^
M1	11	2	13		
*TNM stage*					
I + II	5	12	17	6.057	0.014^*∗*^
III + IV	72	46	118		
*CEA (ng/ml)*					
≤5	56	43	99	0.034	0.854
>5	21	15	36		
*HER2*					
Negative	65	51	116	0.338	0.561
Positive	12	7	19		
*PD-L1*					
Negative	42	40	82	2.885	0.089
Positive	35	18	53		

^*∗*^Statistically significant (*p* < 0.05).

## Data Availability

The datasets and supporting data are available from the corresponding author upon reasonable request.

## Supplementary Materials

Supplementary Materials Figure S1: BPC chromatogram. Figure S2: mass 219.0286 with 30 ppm mass accuracy. Figure S3: mass 365.0903 with 30 ppm mass accuracy. Figure S4: mass 381.0808 with 30 ppm mass accuracy. Figure S5: mass 248.1147 with 30 ppm mass accuracy. Figure S6: mass 241.1557 with 30 ppm mass accuracy. Figure S7: mass 609.2589 with 30 ppm mass accuracy. Figure S8: mass 369.3534 with 30 ppm mass accuracy. Figure S9: the peripheral blood routine and the liver and kidney function showed no abnormalities. (A) The blood routine of mice in different groups. (B) The liver and kidney function in different groups. Table S1: the LC-MS component analysis of the Huaier n-butanol extract. Table S2: univariate and multivariate Cox analyses of prognostic factors for GC.
