# Supplementary material for: Huaier Inhibits Gastric Cancer Growth and Hepatic Metastasis by Reducing Syntenin Expression and STAT3 Phosphorylation
Source: J Oncol. 2022 Jun 15;2022:6065516. doi: 10.1155/2022/6065516 (PMC9217535; doi:10.1155/2022/6065516)

Supplementary Material

**Syntenin expression is related to poor prognosis in gastric cancer and plays a key role in the inhibitory effect of Huaier on gastric cancer hepatic metastasis**

Yunfu Shi^1,2#^, Li Yuan^3,4,5#^, Jingli Xu^2^, Handong Xu^2^, Lijing Wang^3^, Ling Huang^3^, Zhiyuan Xu^3,4,5^*, Jiangjiang Qin^3,4,5^*, Xiangdong Cheng^3,4,5^*

**1. The main components of Huaier n-butanol Extract by LC-MS**

1. 1 Methods

1.1.1Treatment of Huaier n-butanol Extract

1mg Huaier n-butanol Extract was added with 1ml of precooled methanol, vibrated for 20min, centrifuged with 10000g at 4 ℃ for 10min, 600μl of which was lyophilized, and then dissolved in 100μl methanol/water(4:1), and 4μl of supernatant was taken for experiment.

1.1.2 Chromatographic conditions

Chromatographic column: waters UPLC BEH C18(1.8μm 2.1mm*50mm).

mobile phase: A (0.1% formic acid in water) and B (acetonitrile). Elution procedure: 2% B was maintained for 1 min, then increased to 80% within 24 min, and reached 100% at 26 min, after 4 min, the column was balanced for 5 min. The flow rate was 0.35 ml/min, the injection volume was 4μl, the equilibrium time was 5 min before each injection, and the column temperature was 50 ℃.

1.1.3 Mass spectrometry identification

Positive ion detection mode ESI ionization mode was adopted, and the mass scanning range was 80-1000m/Z. Nitrogen was used in various gas paths. Mass spectrum parameters were shown in the following table.

| Survey Ion mode & Polarity: | ESI+ |
| --- | --- |
| Mass: | 80-1000M/Z |
| Capillary: | 3000V |
| Sampling Cone: | 35V |
| Desolvation Temperature: | 300℃ |
| Desolvation Gas Flow: | 500L/H |
| Cone Gas Flow: | 50L/H |
| Source Temperature: | 100℃ |
| Signal to Noise Ratio: | 5 |

1.2 Results

1.2.1 BPC Chromatogram (Figure S1)

1.2.2 Mass spectrum peak and compound after normalization

1.2.2.1 Mass 219.0286 with 30 ppm mass accuracy (Figure S2)

1.2.2.2 Mass 365.0903 with 30 ppm mass accuracy (Figure S3)

1.2.2.3 Mass 381.0808 with 30 ppm mass accuracy (Figure S4)

1.2.2.4 Mass 248.1147 with 30 ppm mass accuracy (Figure S5)

1.2.2.5 Mass 241.1557 with 30 ppm mass accuracy (Figure S6)

1.2.2.6 Mass 609.2589 with 30 ppm mass accuracy (Figure S7)

1.2.2.7 Mass 369.3534 with 30 ppm mass accuracy (Figure S8)

1.2.3 The LC-MS Component Analysis of Huaier n-butanol Extract

**Table S1** The LC-MS Component Analysis of Huaier n-butanol Extract

| No. | Molecular formula | Potential substance |
| --- | --- | --- |
| 1 | C12H10S2 | 4,4'-Biphenyldithiol |
| 2 | C12H10S2 | Diphenyl disulfide |
| 3 | C8H10O5S | Tyrosol 4-sulfate |
| 4 | C15H16N4O5S | Sulfometuron-Methyl |
| 5 | C17H16O9 | Xabthotoxol glucoside |
| 6 | C14H20O10S | 4-Methoxybenzyl O-(2-sulfoglucoside) |
| 7 | C13H21N2O7PS | O-Acetylserine |
| 8 | C10H18CIN3O2 | Semustine |
| 9 | C10H17NO6 | Malonylcarnitine |
| 10 | C10H17NO6 | Valinopine |
| 11 | C10H17NO6 | Linamarin |
| 12 | C16H13N3 | Yellow AB |
| 13 | C12H20N2O3 | Pirbuterol |
| 14 | C17H20O | Dehydrofalcarinone |
| 15 | C36H36N2O7 | Somniferine |
| 16 | C31H36N4O9 | Tyr Tyr Tyr Thr |
| 17 | C31H36N4O9 | Tyr Tyr Thr Tyr |
| 18 | C31H36N4O9 | Tyr Thr Tyr Tyr |
| 19 | C31H36N4O9 | Thr Tyr Tyr Tyr |
| 20 | C27H44 | 3-Deoxyvitamin D3 |
| 21 | C22H44N2O2 | Glyodin |

**2. Supplementary Figure S9**

**
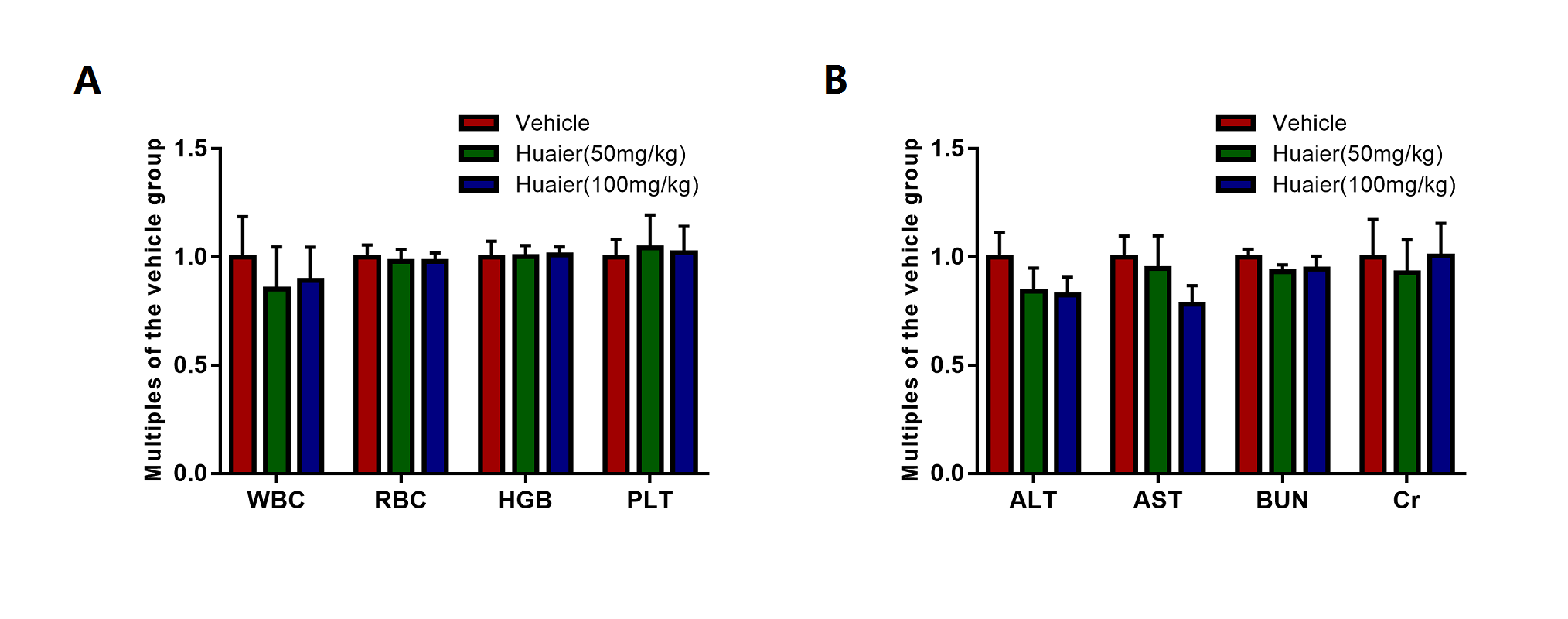
**

**Figure S9.** The peripheral blood routine and liver and kidney function showed no abnormalities. (A) The blood routine of mice in different groups. (B) The liver and kidney function in different groups.

**3. Supplementary Table S2**

**Table S2** Univariate and multivariate Cox analyses of prognostic factors for GC.

| Facts | Univariate analysis | | Multivariate analysis | |
| --- | --- | --- | --- | --- |
|  | HR (95%CI) | P value | HR (95%CI) | P value |
| Age (year) | | | | |
| >65 vs. ≤65 | 1.367 (0.857-2.178) | 0.189 | 1.313 (0.819-2.107) | 0.258 |
| Sex | | | | |
| Male vs. female | 0.868 (0.525-1.436) | 0.581 |  |  |
| Borrmann type | | | | |
| Ⅰ+Ⅱ vs. Ⅲ+Ⅳ | 0.484 (0.308-0.762) | **0.002*** | 0.475(0.297-0.761) | **0.002*** |
| Lauren type | | **0.014*** |  | **0.004*** |
| Diffuse vs. intestinal | 1.564 (0.941-2.600) | 0.085 | 1.332 (0.787-2.253) | 0.285 |
| Mixed vs. intestinal | 2.413 (1.312-4.440) | **0.005*** | 2.978(1.573-5.637) | **0.001*** |
| Grade of differentiation | | | | |
| Well+moderate vs. poor+not | 0.750 (0.478-1.177) | 0.211 |  |  |
| T stage | | | | |
| T1 vs. T2+T3+T4 | 0.593 (0.082-4.268) | 0.604 |  |  |
| N stage | | | | |
| N0 vs. N1+N2+N3 | 0.713 (0.260-1.960) | 0.512 |  |  |
| M stage | | | | |
| M0 vs. M1 | 0.230 (0.121-0.436) | **<0.001*** | 0.361 (0.182-0.715) | **0.003*** |
| TNM stage | | | | |
| Ⅰ+Ⅱ vs. Ⅲ+Ⅳ | 0.606 (0.279-1.320) | 0.208 |  |  |
| Syntenin expression | | | | |
| High vs. low | 2.119 (1.308-3.431) | **0.002*** | 1.851(1.111-3.083) | **0.018*** |
| CEA (ng/ml) | | | | |
| >5 vs. ≤5 | 1.052 (0.626-1.770) | 0.847 |  |  |
| HER2 | | | | |
| Positive vs. negative | 0.881 (0.465-1.670) | 0.698 |  |  |
| PD-L1 | | | | |
| Positive vs. negative | 1.165 (0.736-1.846) | 0.514 |  |  |

*****Statistically significant (p<0.05).

**4.Original Data**

**4.1 The data of** **MGC803 orthotopic GC mouse model**

**Table S3** The body weights of mice in two groups (g)

| Group | No. | Day 0 | Day 3 | Day 6 | Day 9 | Day 12 | Day 15 | Day 18 | Day 21 | Day 24 | Day 27 |
| --- | --- | --- | --- | --- | --- | --- | --- | --- | --- | --- | --- |
| Vehicle | 32 | 20.5 | 20.5 | 18.8 | 19.8 | 22.1 | 22.8 | 24.0 | 25.5 | 24.9 | 24.1 |
|  | 15 | 18.3 | 19.6 | 20.1 | 21.6 | 22.9 | 22.9 | 23.6 | 24.7 | 24.6 | 24.4 |
|  | 39 | 19.1 | 20.3 | 20.8 | 21.6 | 23.2 | 23.7 | 24.4 | 25.3 | 25.2 | 25.6 |
|  | 37 | 17.8 | 19.1 | 19.4 | 20.3 | 22.8 | 23.6 | 24.6 | 24.8 | 24.5 | 24.5 |
|  | 40 | 17.2 | 18.0 | 17.7 | 18.1 | 19.9 | 19.8 | 20.9 | 22.7 | 22.4 | 22.2 |
|  | 3 | 17.2 | 18.4 | 16.8 | 17.5 | 18.5 | 18.8 | 20.7 | 22.2 | 21.5 | 21.9 |
|  | 112 | 20.7 | 21.0 | 21.4 | 20.9 | 21.9 | 22.4 | 22.2 | 22.9 | 22.7 | 23.0 |
|  | 104 | 21.1 | 20.9 | 22.0 | 22.4 | 23.3 | 24.3 | 24.3 | 24.5 | 24.4 | 25.4 |
|  | 105 | 21.9 | 22.1 | 22.7 | 22.5 | 23.2 | 23.4 | 23.7 | 23.5 | 23.1 | 23.9 |
|  | 109 | 21.3 | 20.7 | 21.3 | 20.9 | 21.4 | 21.8 | 21.7 | 22.0 | 22.0 | 22.8 |
| Huaier  (100mg/kg) | 18 | 18.4 | 20.3 | 18.5 | 20.9 | 22.3 | 22.5 | 24.0 | 25.6 | 25.8 | 25.0 |
|  | 14 | 18.5 | 19.5 | 19.3 | 20.6 | 22.0 | 22.3 | 23.2 | 24.2 | 24.5 | 24.4 |
|  | 25 | 19.0 | 20.2 | 19.5 | 21.6 | 23.2 | 23.4 | 24.7 | 26.2 | 25.1 | 25.6 |
|  | 4 | 17.7 | 18.8 | 18.8 | 19.8 | 20.6 | 21.1 | 22.0 | 22.5 | 23.0 | 22.5 |
|  | 10 | 19.7 | 20.9 | 21.6 | 22.2 | 23.4 | 23.5 | 24.0 | 25.2 | 25.3 | 24.9 |
|  | 106 | 19.1 | 18.5 | 19.6 | 19.3 | 19.7 | 21.1 | 21.0 | 21.4 | 21.1 | 21.3 |
|  | 110 | 20.2 | 20.7 | 21.2 | 20.6 | 21.3 | 22.5 | 22.5 | 23.1 | 23.0 | 23.0 |
|  | 108 | 20.3 | 20.0 | 20.4 | 20.9 | 21.5 | 22.4 | 22.5 | 22.6 | 22.0 | 22.4 |
|  | 102 | 21.6 | 21.3 | 22.0 | 21.5 | 22.3 | 23.9 | 23.7 | 24.1 | 23.9 | 24.1 |
|  | 107 | 21.8 | 22.4 | 23.3 | 23.5 | 23.3 | 24.3 | 24.5 | 24.4 | 24.4 | 25.6 |

**Table S4 The fluorescence values of in vivo imaging in two groups (×10^8^ photons/sec)**

| Group | No. | Day 0 | Day 7 | Day 14 | Day 21 | Day 28 |
| --- | --- | --- | --- | --- | --- | --- |
| Vehicle | 32 | 0.321 | 1.2 | 0.526 | 5.43 | 4.5 |
|  | 15 | 2.33 | 0.508 | 2.23 | 18.7 | 14.1 |
|  | 39 | 0.251 | 0.181 | 0.0548 | 0.245 | 0.179 |
|  | 37 | 1.05 | 7.12 | 11.7 | 27.2 | 25.9 |
|  | 40 | 0.0735 | 0.878 | 0.672 | 0.324 | 0.613 |
|  | 3 | 0.231 | 3.33 | 24 | 2.98 | 37.5 |
|  | 112 | 0.938 | 5.28 | 8.77 | 22 | 38.9 |
|  | 104 | 0.134 | 0.275 | 0.43 | 0.21 | 0.331 |
|  | 105 | 0.225 | 0.315 | 3.83 | 11.5 | 12.9 |
|  | 109 | 0.0868 | 1.07 | 0.33 | 0.71 | 0.524 |
| Huaier  (100mg/kg) | 18 | 0.0187 | 0.00261 | 0.0365 | 0.351 | 0.51 |
|  | 14 | 2.16 | 0.0326 | 1.64 | 5.18 | 0.925 |
|  | 25 | 0.417 | 0.0376 | 2.97 | 1.75 | 0.126 |
|  | 4 | 1.66 | 6.17 | 8.52 | 10.9 | 3.46 |
|  | 10 | 0.478 | 0.168 | 0.0299 | 1.92 | 0.281 |
|  | 106 | 0.0238 | 0.0135 | 0.0195 | 0.00793 | 0.0258 |
|  | 110 | 0.0138 | 0.0727 | 0.108 | 0.155 | 0.0948 |
|  | 108 | 0.225 | 0.0607 | 0.0826 | 0.377 | 0.371 |
|  | 102 | 0.391 | 0.495 | 1.58 | 2.66 | 10.7 |
|  | 107 | 0.104 | 0.126 | 0.724 | 1.4 | 2.11 |

**4.2 The data of MKN74 orthotopic GC mouse model**

**Table S5** The body weights of mice in three groups (g)

| Group | No. | Day 0 | Day 3 | Day 6 | Day 9 | Day 12 | Day 15 | Day 18 | Day 21 | Day 24 | Day 27 | Day 30 |
| --- | --- | --- | --- | --- | --- | --- | --- | --- | --- | --- | --- | --- |
| Vehicle | 1 | 21.2 | 22.4 | 23.1 | 23.8 | 24.0 | 24.1 | 24.7 | 24.7 | 24.5 | 24.3 | 24.3 |
|  | 8 | 21.5 | 23.1 | 23.2 | 23.3 | 23.5 | 23.7 | 24.1 | 22.6 | 23.4 | 23.6 | 22.1 |
|  | 10 | 19.9 | 21.2 | 21.3 | 21.3 | 22.3 | 22.1 | 23.2 | 22.8 | 22.2 | 21.3 | 21.2 |
|  | 13 | 19.0 | 21.0 | 21.3 | 21.5 | 23.2 | 22.8 | 23.7 | 23.8 | 23.5 | 22.8 | 20.1 |
|  | 20 | 19.9 | 21.5 | 22.3 | 23.1 | 24.0 | 23.7 | 24.5 | 24.7 | 24.0 | 23.7 | 23.2 |
|  | 27 | 20.0 | 21.8 | 22.4 | 22.7 | 23.1 | 22.9 | 23.3 | 23.2 | 22.7 | 21.5 | 20.5 |
|  | 29 | 20.6 | 22.2 | 22.8 | 22.8 | 23.9 | 23.7 | 23.9 | 24.1 | 23.8 | 23.1 | 23.1 |
|  | 39 | 20.3 | 21.9 | 22.6 | 23.5 | 23.2 | 24.3 | 24.2 | 24.0 | 24.4 | 23.1 | 23.2 |
|  | 42 | 20.0 | 21.2 | 21.5 | 22.8 | 21.9 | 21.8 | 22.1 | 23.1 | 22.6 | 23.4 | 22.7 |
|  | 43 | 20.7 | 23.0 | 23.6 | 24.1 | 24.1 | 23.5 | 24.4 | 25.7 | 25.5 | 24.8 | 23.5 |
| Huaier  (50mg/kg) | 5 | 18.7 | 20.5 | 21.0 | 21.2 | 22.1 | 22.0 | 22.6 | 22.8 | 23.4 | 22.4 | 22.2 |
|  | 7 | 20.0 | 21.7 | 22.8 | 23.1 | 23.2 | 23.8 | 24.0 | 24.4 | 23.8 | 23.2 | 23.1 |
|  | 15 | 20.1 | 21.1 | 21.7 | 22.4 | 23.6 | 23.5 | 23.8 | 24.1 | 23.6 | 23.4 | 22.9 |
|  | 19 | 20.4 | 21.9 | 22.4 | 22.8 | 23.1 | 23.8 | 23.7 | 24.8 | 24.3 | 23.4 | 22.8 |
|  | 23 | 21.6 | 22.6 | 23.3 | 23.9 | 23.8 | 24.6 | 24.2 | 25.3 | 25.0 | 24.8 | 24.5 |
|  | 24 | 19.3 | 20.5 | 21.5 | 22.3 | 21.8 | 22.0 | 21.9 | 22.0 | 21.8 | 20.5 | 20.2 |
|  | 26 | 19.6 | 21.7 | 22.5 | 22.6 | 23.2 | 23.0 | 23.9 | 24.4 | 23.9 | 22.7 | 22.4 |
|  | 31 | 22.7 | 23.8 | 24.6 | 25.4 | 26.7 | 26.1 | 26.4 | 27.2 | 27.0 | 27.3 | 26.9 |
|  | 33 | 21.3 | 23.3 | 24.6 | 24.9 | 24.9 | 24.5 | 25.3 | 26.5 | 24.8 | 23.9 | 23.7 |
|  | 41 | 19.6 | 21.0 | 21.9 | 22.6 | 23.1 | 22.7 | 22.9 | 23.3 | 24.1 | 23.8 | 24.2 |

| Group | No. | Day 0 | Day 3 | Day 6 | Day 9 | Day 12 | Day 15 | Day 18 | Day 21 | Day 24 | Day 27 | Day 30 |
| --- | --- | --- | --- | --- | --- | --- | --- | --- | --- | --- | --- | --- |
| Huaier  (100mg/kg) | 3 | 20.1 | 22.4 | 22.5 | 22.6 | 23.9 | 23.9 | 24.0 | 24.5 | 23.9 | 23.3 | 23.5 |
|  | 9 | 19.6 | 21.2 | 22.3 | 23.1 | 23.1 | 23.6 | 23.9 | 24.1 | 25.2 | 24.0 | 24.3 |
|  | 14 | 20.3 | 21.6 | 22.4 | 23.5 | 23.2 | 23.7 | 24.1 | 24.4 | 23.7 | 24.5 | 24.0 |
|  | 21 | 21.4 | 23.5 | 24.0 | 24.8 | 24.7 | 24.6 | 25.3 | 25.9 | 25.2 | 26.1 | 25.9 |
|  | 25 | 21.0 | 22.7 | 23.0 | 23.2 | 23.3 | 23.2 | 23.5 | 23.8 | 24.5 | 24.1 | 23.6 |
|  | 30 | 22.1 | 23.1 | 23.8 | 24.6 | 24.4 | 24.8 | 24.7 | 25.0 | 25.3 | 24.8 | 24.7 |
|  | 35 | 20.0 | 21.0 | 22.1 | 22.9 | 23.1 | 23.6 | 24.1 | 24.0 | 24.2 | 23.5 | 23.5 |
|  | 37 | 20.9 | 22.3 | 22.8 | 23.3 | 22.6 | 22.9 | 23.7 | 23.9 | 24.8 | 23.9 | 23.8 |
|  | 38 | 20.5 | 22.1 | 22.8 | 23.8 | 24.0 | 24.5 | 24.6 | 25.2 | 24.7 | 23.8 | 24.2 |
|  | 40 | 19.7 | 21.1 | 21.9 | 22.8 | 23.1 | 23.6 | 23.8 | 23.6 | 23.1 | 22.7 | 21.5 |

**Table S6 The fluorescence values of in vivo imaging in three groups (×10^8^ photons/sec)**

| Group | No. | Day 0 | Day 10 | Day 20 | Day 30 |
| --- | --- | --- | --- | --- | --- |
| Vehicle | 1 | 0.58 | 0.355 | 0.358 | 2.01 |
|  | 8 | 2.16 | 1.04 | 2.36 | 5.29 |
|  | 10 | 1.03 | 1.91 | 1.42 | 9.09 |
|  | 13 | 1.34 | 3.02 | 2.5 | 14 |
|  | 20 | 0.598 | 0.235 | 0.425 | 0.878 |
|  | 27 | 0.547 | 0.956 | 8.12 | 8.26 |
|  | 29 | 1.74 | 2.28 | 2.36 | 9.07 |
|  | 39 | 1.22 | 1.28 | 2.21 | 10.3 |
|  | 42 | 0.915 | 2.6 | 8.85 | 5.93 |
|  | 43 | 0.853 | 1.8 | 1.53 | 2.87 |
| Huaier  (50mg/kg) | 5 | 3.72 | 2.21 | 7.1 | 0.724 |
|  | 7 | 0.529 | 0.214 | 1.74 | 2.14 |
|  | 15 | 1.43 | 1.36 | 0.403 | 2.42 |
|  | 19 | 0.0863 | 1.61 | 3.85 | 5 |
|  | 23 | 1.17 | 0.56 | 0.449 | 3.21 |
|  | 24 | 1.28 | 0.801 | 2.33 | 1.1 |
|  | 26 | 0.798 | 0.278 | 0.437 | 2.79 |
|  | 31 | 0.683 | 0.185 | 0.553 | 0.318 |
|  | 33 | 1.87 | 2.14 | 4.37 | 3.05 |
|  | 41 | 1.1 | 0.876 | 0.872 | 0.328 |
| Huaier  (100mg/kg) | 3 | 1.49 | 2.19 | 0.144 | 0.956 |
|  | 9 | 0.59 | 0.788 | 0.0996 | 0.301 |
|  | 14 | 0.273 | 0.0062 | 0.0616 | 0.038 |
|  | 21 | 1.12 | 1.09 | 0.159 | 0.974 |
|  | 25 | 1.09 | 0.914 | 0.484 | 0.398 |
|  | 30 | 2.51 | 0.422 | 1.63 | 1.07 |
|  | 35 | 0.451 | 0.0975 | 0.0655 | 0.0745 |
|  | 37 | 1.32 | 0.535 | 0.428 | 0.0324 |
|  | 38 | 1.36 | 2.02 | 4.6 | 6.07 |
|  | 40 | 0.747 | 0.767 | 0.735 | 0.991 |

**4.3 The data of MGC803 GC hepatic metastasis mouse model**

**Table S7** The body weights of mice in three groups (g)

| Group | No. | Day 0 | Day 2 | Day 4 | Day 6 | Day 8 | Day 10 |
| --- | --- | --- | --- | --- | --- | --- | --- |
| Vehicle | 76 | 23.1 | 24.3 | 24.9 | 25.6 | 25.8 | 26.0 |
|  | 81 | 23.8 | 24.6 | 25.3 | 25.8 | 26.4 | 26.5 |
|  | 82 | 22.0 | 23.5 | 23.7 | 24.6 | 25.0 | 25.6 |
|  | 86 | 22.3 | 23.7 | 24.1 | 24.9 | 25.6 | 26.2 |
|  | 89 | 23.7 | 24.7 | 25.1 | 25.8 | 26.8 | 26.6 |
|  | 92 | 21.9 | 22.8 | 23.5 | 24.4 | 25.1 | 25.5 |
| Huaier  (50mg/kg) | 66 | 21.5 | 22.5 | 23.2 | 23.5 | 24.8 | 25.6 |
|  | 77 | 23.0 | 23.8 | 24.0 | 24.8 | 25.3 | 25.9 |
|  | 78 | 22.5 | 23.4 | 24.5 | 24.9 | 25.2 | 25.7 |
|  | 83 | 21.8 | 22.6 | 23.2 | 23.4 | 24.8 | 25.5 |
|  | 87 | 21.1 | 21.6 | 22.7 | 23.2 | 23.8 | 24.5 |
|  | 88 | 21.8 | 22.3 | 23.8 | 24.4 | 24.4 | 25.2 |
| Huaier  (100mg/kg) | 63 | 22.0 | 21.6 | 22.3 | 23.2 | 23.8 | 23.7 |
|  | 72 | 22.6 | 23.2 | 22.5 | 23.7 | 24.0 | 24.5 |
|  | 75 | 23.3 | 23.0 | 22.8 | 23.6 | 24.2 | 24.9 |
|  | 80 | 21.3 | 21.7 | 22.6 | 23.2 | 23.9 | 24.5 |
|  | 85 | 22.8 | 22.4 | 23.2 | 24.1 | 24.8 | 25.6 |
|  | 90 | 20.0 | 20.4 | 21.0 | 22.2 | 22.6 | 22.9 |

**Table S8 The fluorescence values of in vivo imaging in three groups (×10^7^ photons/sec)**

| Group | No. | Day 0 | Day 5 | Day 10 |
| --- | --- | --- | --- | --- |
| Vehicle | 76 | 0.6399 | 0.2336 | 0.1154 |
|  | 81 | 3.425 | 2.064 | 0.5274 |
|  | 82 | 5.274 | 1.715 | 4.605 |
|  | 86 | 1.128 | 5.71 | 8.91 |
|  | 89 | 2.605 | 2.00 | 9.919 |
|  | 92 | 2.586 | 1.277 | 0.2393 |
| Huaier  (50mg/kg) | 66 | 0.625 | 0.009977 | 0.01039 |
|  | 77 | 2.497 | 2.49 | 1.602 |
|  | 78 | 2.172 | 1.517 | 0.01567 |
|  | 83 | 4.302 | 5.081 | 1.141 |
|  | 87 | 3.818 | 0.3914 | 0.01149 |
|  | 88 | 2.59 | 0.816 | 0.9046 |
| Huaier  (100mg/kg) | 63 | 3.6 | 0.00297 | 0.01012 |
|  | 72 | 0.996 | 0.01509 | 0.01168 |
|  | 75 | 1.65 | 0.01723 | 0.01338 |
|  | 80 | 3.841 | 0.3567 | 0.01613 |
|  | 85 | 2.447 | 0.9208 | 0.1414 |
|  | 90 | 3.408 | 3.203 | 0.7717 |

**4.4 The data of CCK-8 assays**

**Table S9 The optical delnsity (OD) value of each cell line**

| Cell lines | Time | control | Huaier (μg/ml) | | | | | | | | |
| --- | --- | --- | --- | --- | --- | --- | --- | --- | --- | --- | --- |
|  |  |  | 0 | 20 | 40 | 60 | 80 | 100 | 120 | 160 | 200 |
| GES-1 | 24 h | 0.107492 | 1.093987 | 1.08559 | 1.06334 | 1.04384 | 1.01969 | 1.02185 | 0.904098 | 0.827551 | 0.636826 |
|  |  | 0.105518 | 1.13023 | 1.05841 | 1.07518 | 1.10328 | 1.0388 | 0.992031 | 0.89346 | 0.810176 | 0.652552 |
|  |  | 0.10689 | 1.1115 | 1.07724 | 1.05513 | 1.09231 | 1.03121 | 1.02148 | 0.933546 | 0.799593 | 0.666723 |
|  |  | 0.108 | 1.082924 | 1.06303 | 1.05004 | 1.06933 | 1.05405 | 0.996949 | 0.855405 | 0.816813 | 0.650423 |
|  | 48 h | 0.104624 | 1.13614 | 1.11854 | 1.17211 | 1.07668 | 0.888732 | 0.820507 | 0.733047 | 0.620962 | 0.456574 |
|  |  | 0.106329 | 1.23466 | 1.16273 | 1.14253 | 1.09981 | 0.902105 | 0.817719 | 0.68624 | 0.619271 | 0.430153 |
|  |  | 0.10556 | 1.2578 | 1.1692 | 1.13533 | 1.07574 | 0.90399 | 0.870235 | 0.65599 | 0.618181 | 0.436654 |
|  |  | 0.104732 | 1.13949 | 1.14683 | 1.18224 | 1.08589 | 0.880014 | 0.8609 | 0.660329 | 0.643598 | 0.42519 |
|  | 72 h | 0.104481 | 1.18986 | 1.09012 | 1.10868 | 0.878946 | 0.817445 | 0.555807 | 0.482413 | 0.344076 | 0.221767 |
|  |  | 0.105841 | 1.16987 | 1.127 | 1.14831 | 0.96137 | 0.824564 | 0.615176 | 0.526338 | 0.324922 | 0.218945 |
|  |  | 0.106038 | 1.17487 | 1.12995 | 1.04975 | 0.98131 | 0.835753 | 0.644575 | 0.514583 | 0.315111 | 0.222363 |
|  |  | 0.10496 | 1.11703 | 1.08262 | 1.06241 | 0.96657 | 0.828717 | 0.618075 | 0.533186 | 0.327333 | 0.215148 |
| MGC803 | 24 h | 0.117878 | 1.07142 | 1.098767 | 1.001508 | 0.837503 | 0.720596 | 0.605111 | 0.536167 | 0.415046 | 0.347341 |
|  |  | 0.11716 | 1.08225 | 1.071526 | 1.003182 | 0.845109 | 0.747934 | 0.619578 | 0.555288 | 0.435425 | 0.355171 |
|  |  | 0.12048 | 1.08885 | 1.085063 | 1.007263 | 0.845843 | 0.755876 | 0.627188 | 0.581275 | 0.423018 | 0.345434 |
|  |  | 0.119638 | 1.1387 | 1.091227 | 1.026647 | 0.852927 | 0.746263 | 0.643329 | 0.563692 | 0.449298 | 0.334572 |
|  | 48 h | 0.109382 | 1.09701 | 1.06497 | 0.979061 | 0.765995 | 0.605816 | 0.382395 | 0.27847 | 0.257644 | 0.249121 |
|  |  | 0.110763 | 1.08461 | 1.05998 | 0.968299 | 0.811359 | 0.578432 | 0.375025 | 0.273649 | 0.279018 | 0.239917 |
|  |  | 0.109756 | 1.07627 | 1.09155 | 0.946325 | 0.791147 | 0.63664 | 0.387598 | 0.31011 | 0.272871 | 0.241472 |
|  |  | 0.1092 | 1.06939 | 1.09739 | 0.946973 | 0.762259 | 0.59591 | 0.381165 | 0.286048 | 0.277924 | 0.216374 |

| Cell lines | Time | control | Huaier (μg/ml) | | | | | | | | |
| --- | --- | --- | --- | --- | --- | --- | --- | --- | --- | --- | --- |
|  |  |  | 0 | 20 | 40 | 60 | 80 | 100 | 120 | 160 | 200 |
| MGC803 | 72 h | 0.106798 | 1.10087 | 1.06354 | 0.847466 | 0.475332 | 0.319745 | 0.262578 | 0.213076 | 0.161774 | 0.152169 |
|  |  | 0.103976 | 1.07116 | 1.10287 | 0.886362 | 0.511868 | 0.346111 | 0.253318 | 0.209841 | 0.171619 | 0.151941 |
|  |  | 0.105672 | 1.11105 | 1.08952 | 0.912607 | 0.493858 | 0.342013 | 0.250442 | 0.203745 | 0.17476 | 0.153432 |
|  |  | 0.105626 | 1.08548 | 1.07698 | 0.897381 | 0.501078 | 0.325951 | 0.262125 | 0.177929 | 0.170401 | 0.155674 |
| MKN74 | 24 h | 0.135579 | 1.07958 | 1.0833 | 1.017468 | 0.873089 | 0.834418 | 0.714054 | 0.672676 | 0.561445 | 0.508079 |
|  |  | 0.132235 | 1.19816 | 1.14836 | 1.021514 | 0.89058 | 0.810896 | 0.705153 | 0.673901 | 0.559464 | 0.516272 |
|  |  | 0.132792 | 1.18543 | 1.13919 | 1.039478 | 0.874142 | 0.803565 | 0.674855 | 0.662773 | 0.59068 | 0.516735 |
|  |  | 0.132885 | 1.19985 | 1.11905 | 1.029044 | 0.890769 | 0.850915 | 0.69803 | 0.665183 | 0.571968 | 0.507183 |
|  | 48 h | 0.110637 | 1.03326 | 0.92922 | 0.86875 | 0.719467 | 0.616513 | 0.569387 | 0.468337 | 0.417251 | 0.338888 |
|  |  | 0.109554 | 1.13654 | 0.92339 | 0.844471 | 0.691561 | 0.648113 | 0.587937 | 0.457286 | 0.422108 | 0.356732 |
|  |  | 0.109649 | 1.12022 | 0.91189 | 0.845477 | 0.684041 | 0.657952 | 0.534567 | 0.453722 | 0.446659 | 0.352127 |
|  |  | 0.109602 | 1.04456 | 0.92108 | 0.835174 | 0.679178 | 0.621602 | 0.534137 | 0.465088 | 0.398272 | 0.340875 |
|  | 72 h | 0.155035 | 1.14129 | 0.95918 | 0.804268 | 0.711339 | 0.589659 | 0.453514 | 0.373838 | 0.302464 | 0.257363 |
|  |  | 0.154481 | 1.12898 | 0.9831 | 0.803623 | 0.713611 | 0.548194 | 0.468022 | 0.367343 | 0.316996 | 0.274113 |
|  |  | 0.154934 | 1.20558 | 1.02763 | 0.865616 | 0.735026 | 0.57657 | 0.458622 | 0.381739 | 0.311624 | 0.26095 |
|  |  | 0.155358 | 1.21071 | 0.99727 | 0.819742 | 0.637291 | 0.58771 | 0.440015 | 0.388995 | 0.304661 | 0.262554 |
| AZ-521 | 24 h | 0.181871 | 0.8193 | 0.78227 | 0.777877 | 0.67738 | 0.62383 | 0.580564 | 0.524998 | 0.438292 | 0.364767 |
|  |  | 0.181038 | 0.858434 | 0.786178 | 0.74937 | 0.69673 | 0.638981 | 0.609915 | 0.55028 | 0.433997 | 0.322735 |
|  |  | 0.178626 | 0.880306 | 0.832378 | 0.787868 | 0.682483 | 0.653252 | 0.57362 | 0.554921 | 0.475211 | 0.356418 |
|  |  | 0.178751 | 0.875913 | 0.818139 | 0.794358 | 0.70884 | 0.664146 | 0.569263 | 0.53388 | 0.466179 | 0.378486 |

| Cell lines | Time | control | Huaier (μg/ml) | | | | | | | | |
| --- | --- | --- | --- | --- | --- | --- | --- | --- | --- | --- | --- |
|  |  |  | 0 | 20 | 40 | 60 | 80 | 100 | 120 | 160 | 200 |
| AZ-521 | 48 h | 0.167036 | 0.889289 | 0.810796 | 0.63486 | 0.450007 | 0.435266 | 0.327277 | 0.314998 | 0.284587 | 0.254911 |
|  |  | 0.167951 | 0.907575 | 0.80038 | 0.631849 | 0.462907 | 0.434099 | 0.322247 | 0.316458 | 0.285389 | 0.254277 |
|  |  | 0.167139 | 0.910906 | 0.811582 | 0.66987 | 0.446115 | 0.438651 | 0.326394 | 0.31712 | 0.290919 | 0.256789 |
|  |  | 0.17175 | 0.892919 | 0.814764 | 0.630627 | 0.430133 | 0.424622 | 0.320511 | 0.319967 | 0.285531 | 0.251663 |
|  | 72 h | 0.162752 | 0.836306 | 0.632281 | 0.421959 | 0.29379 | 0.239478 | 0.216271 | 0.200281 | 0.20818 | 0.197911 |
|  |  | 0.163572 | 0.819671 | 0.652017 | 0.450853 | 0.292992 | 0.234821 | 0.236389 | 0.199011 | 0.207421 | 0.197865 |
|  |  | 0.168974 | 0.803464 | 0.688154 | 0.452691 | 0.288758 | 0.244783 | 0.212864 | 0.200755 | 0.20673 | 0.203585 |
|  |  | 0.165138 | 0.820569 | 0.673579 | 0.461222 | 0.283498 | 0.240749 | 0.221194 | 0.200047 | 0.208483 | 0.208453 |
| MKN28 | 24 h | 0.1161 | 1.20978 | 1.12601 | 1.0342 | 0.933001 | 0.880595 | 0.776147 | 0.681305 | 0.592368 | 0.519401 |
|  |  | 0.114233 | 1.20948 | 1.17524 | 1.041171 | 0.96154 | 0.902659 | 0.811855 | 0.729314 | 0.625475 | 0.563126 |
|  |  | 0.113058 | 1.20263 | 1.16991 | 1.057796 | 0.961978 | 0.883625 | 0.844692 | 0.713579 | 0.601906 | 0.566074 |
|  |  | 0.116146 | 1.20876 | 1.12728 | 1.032099 | 0.929472 | 0.861332 | 0.794426 | 0.695882 | 0.585363 | 0.520011 |
|  | 48 h | 0.123681 | 1.08447 | 0.934928 | 0.835003 | 0.676626 | 0.603466 | 0.573527 | 0.520042 | 0.403381 | 0.344357 |
|  |  | 0.124194 | 1.05916 | 0.897304 | 0.849364 | 0.684024 | 0.612607 | 0.596916 | 0.530768 | 0.416834 | 0.337116 |
|  |  | 0.123132 | 1.03068 | 0.926497 | 0.862113 | 0.68799 | 0.625194 | 0.590777 | 0.533939 | 0.429873 | 0.337039 |
|  |  | 0.12485 | 1.04476 | 0.892912 | 0.828331 | 0.668805 | 0.631556 | 0.549196 | 0.538624 | 0.412868 | 0.344173 |
|  | 72 h | 0.114734 | 1.13976 | 0.917444 | 0.706777 | 0.542145 | 0.527587 | 0.420115 | 0.384979 | 0.305473 | 0.254996 |
|  |  | 0.113722 | 1.10794 | 0.925619 | 0.7521 | 0.544509 | 0.488993 | 0.456091 | 0.396858 | 0.311546 | 0.257618 |
|  |  | 0.115063 | 1.13756 | 0.930269 | 0.716781 | 0.548535 | 0.456248 | 0.450071 | 0.407959 | 0.310606 | 0.255936 |
|  |  | 0.11423 | 1.12791 | 0.940663 | 0.716774 | 0.536089 | 0.507603 | 0.448156 | 0.407525 | 0.309894 | 0.261718 |

**Table S10 The optical delnsity (OD) value of MGC803 cells with stable syntenin overexpression and knockdown**

| Cell lines | | control | Huaier (μg/ml) | | | | | | | | |
| --- | --- | --- | --- | --- | --- | --- | --- | --- | --- | --- | --- |
|  |  |  | 0 | 20 | 40 | 60 | 80 | 100 | 120 | 160 | 200 |
| MGC803 | vector | 0.110892 | 1.08291 | 1.04883 | 0.913601 | 0.770213 | 0.604672 | 0.382516 | 0.275594 | 0.216868 | 0.186823 |
|  |  | 0.112292 | 1.07193 | 1.05547 | 0.984258 | 0.809789 | 0.576727 | 0.378162 | 0.269896 | 0.238913 | 0.185387 |
|  |  | 0.111155 | 1.06383 | 1.07997 | 0.959608 | 0.755041 | 0.621136 | 0.386493 | 0.306153 | 0.226754 | 0.177918 |
|  |  | 0.110995 | 1.04753 | 1.08415 | 0.944243 | 0.782683 | 0.569934 | 0.359584 | 0.281485 | 0.223303 | 0.223436 |
|  | syntenin | 0.0992196 | 1.05287 | 0.96614 | 0.956825 | 0.909401 | 0.877784 | 0.799735 | 0.724687 | 0.612058 | 0.453127 |
|  |  | 0.0985392 | 1.01387 | 1.00174 | 0.92882 | 0.904731 | 0.889435 | 0.832738 | 0.691732 | 0.549666 | 0.435756 |
|  |  | 0.0999657 | 1.06005 | 0.983745 | 0.928784 | 0.908177 | 0.837579 | 0.814411 | 0.674039 | 0.579511 | 0.472132 |
|  |  | 0.101734 | 1.02096 | 1.01946 | 0.934756 | 0.91366 | 0.865265 | 0.809329 | 0.654107 | 0.569269 | 0.43449 |
|  | shCtrl | 0.114222 | 0.998797 | 0.977683 | 0.890581 | 0.732227 | 0.533784 | 0.414396 | 0.359311 | 0.239263 | 0.209094 |
|  |  | 0.113141 | 1.00022 | 1.00309 | 0.924263 | 0.709559 | 0.553815 | 0.416798 | 0.353625 | 0.253695 | 0.205492 |
|  |  | 0.114185 | 0.997334 | 1.01982 | 0.897309 | 0.741462 | 0.580052 | 0.421942 | 0.388303 | 0.255373 | 0.206583 |
|  |  | 0.113797 | 1.00381 | 0.993446 | 0.900625 | 0.711089 | 0.559344 | 0.457161 | 0.361443 | 0.256748 | 0.233712 |
|  | shSyntenin | 0.123752 | 0.887438 | 0.645913 | 0.367985 | 0.266109 | 0.221272 | 0.184024 | 0.162469 | 0.151365 | 0.149357 |
|  |  | 0.125604 | 0.860236 | 0.611918 | 0.407503 | 0.280204 | 0.213842 | 0.192513 | 0.166712 | 0.153255 | 0.147452 |
|  |  | 0.127733 | 0.889047 | 0.676401 | 0.398191 | 0.301364 | 0.214828 | 0.195738 | 0.172432 | 0.153014 | 0.145485 |
|  |  | 0.124279 | 0.882557 | 0.617156 | 0.41493 | 0.29087 | 0.233845 | 0.188228 | 0.171571 | 0.152323 | 0.139306 |

**Table S11 The optical delnsity (OD) value of MKN74 cells with stable syntenin overexpression and knockdown**

| Cell lines | | control | Huaier (μg/ml) | | | | | | | | |
| --- | --- | --- | --- | --- | --- | --- | --- | --- | --- | --- | --- |
|  |  |  | 0 | 20 | 40 | 60 | 80 | 100 | 120 | 160 | 200 |
| MKN74 | vector | 0.11016 | 1.04097 | 0.99418 | 0.90114 | 0.788894 | 0.57669 | 0.516715 | 0.478839 | 0.40327 | 0.35305 |
|  |  | 0.109287 | 1.16612 | 1.0126 | 0.871994 | 0.757629 | 0.60514 | 0.510043 | 0.464831 | 0.38942 | 0.357688 |
|  |  | 0.10941 | 1.12916 | 0.98664 | 0.869396 | 0.763452 | 0.623665 | 0.52119 | 0.449055 | 0.402886 | 0.347664 |
|  |  | 0.109068 | 1.05614 | 0.99757 | 0.865827 | 0.759801 | 0.597298 | 0.539652 | 0.483949 | 0.359647 | 0.338544 |
|  | syntenin | 0.12936 | 0.979509 | 0.965577 | 0.872848 | 0.832991 | 0.763094 | 0.697115 | 0.662959 | 0.611457 | 0.552117 |
|  |  | 0.13006 | 0.973973 | 0.954474 | 0.88075 | 0.81488 | 0.803551 | 0.730698 | 0.676701 | 0.619069 | 0.548704 |
|  |  | 0.128943 | 0.985697 | 0.937629 | 0.892336 | 0.789136 | 0.801548 | 0.737382 | 0.680283 | 0.618391 | 0.552171 |
|  |  | 0.12997 | 1.006888 | 0.952365 | 0.849159 | 0.796499 | 0.765021 | 0.673029 | 0.66197 | 0.617843 | 0.565129 |
|  | shCtrl | 0.10814 | 1.11984 | 0.961436 | 0.929805 | 0.765332 | 0.58845 | 0.566793 | 0.542162 | 0.429972 | 0.299468 |
|  |  | 0.107424 | 1.062461 | 0.98926 | 0.89979 | 0.748719 | 0.604698 | 0.599554 | 0.518152 | 0.435803 | 0.317883 |
|  |  | 0.10742 | 1.11447 | 0.977184 | 0.892333 | 0.751096 | 0.613541 | 0.53669 | 0.514753 | 0.451835 | 0.316709 |
|  |  | 0.107255 | 1.08114 | 0.982171 | 0.902557 | 0.746229 | 0.591737 | 0.543629 | 0.546466 | 0.408732 | 0.305642 |
|  | shSyntenin | 0.109942 | 0.940108 | 0.691806 | 0.470435 | 0.331889 | 0.237803 | 0.191036 | 0.18894 | 0.192531 | 0.159726 |
|  |  | 0.110491 | 0.922797 | 0.690072 | 0.48177 | 0.359264 | 0.238271 | 0.196289 | 0.190286 | 0.191737 | 0.158966 |
|  |  | 0.115528 | 0.909834 | 0.699131 | 0.516015 | 0.35904 | 0.235779 | 0.197313 | 0.189897 | 0.193641 | 0.15871 |
|  |  | 0.112844 | 0.922749 | 0.700569 | 0.499911 | 0.369874 | 0.241957 | 0.194167 | 0.196524 | 0.191574 | 0.159888 |

**5. Uncut original image**

5.1 Uncut original image of Western Blot in figure 6.

MGC803 Syntenin MGC803 STAT3


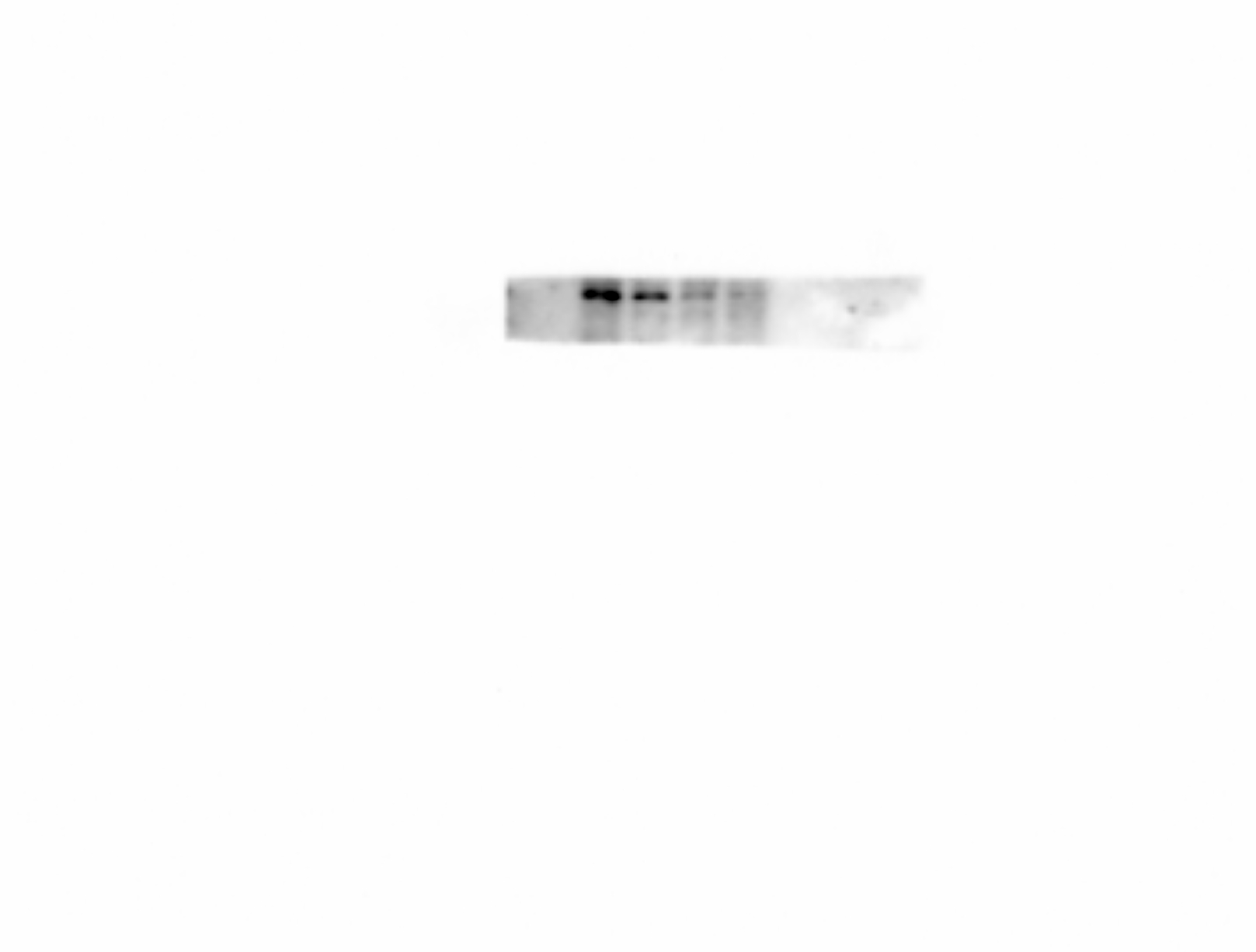

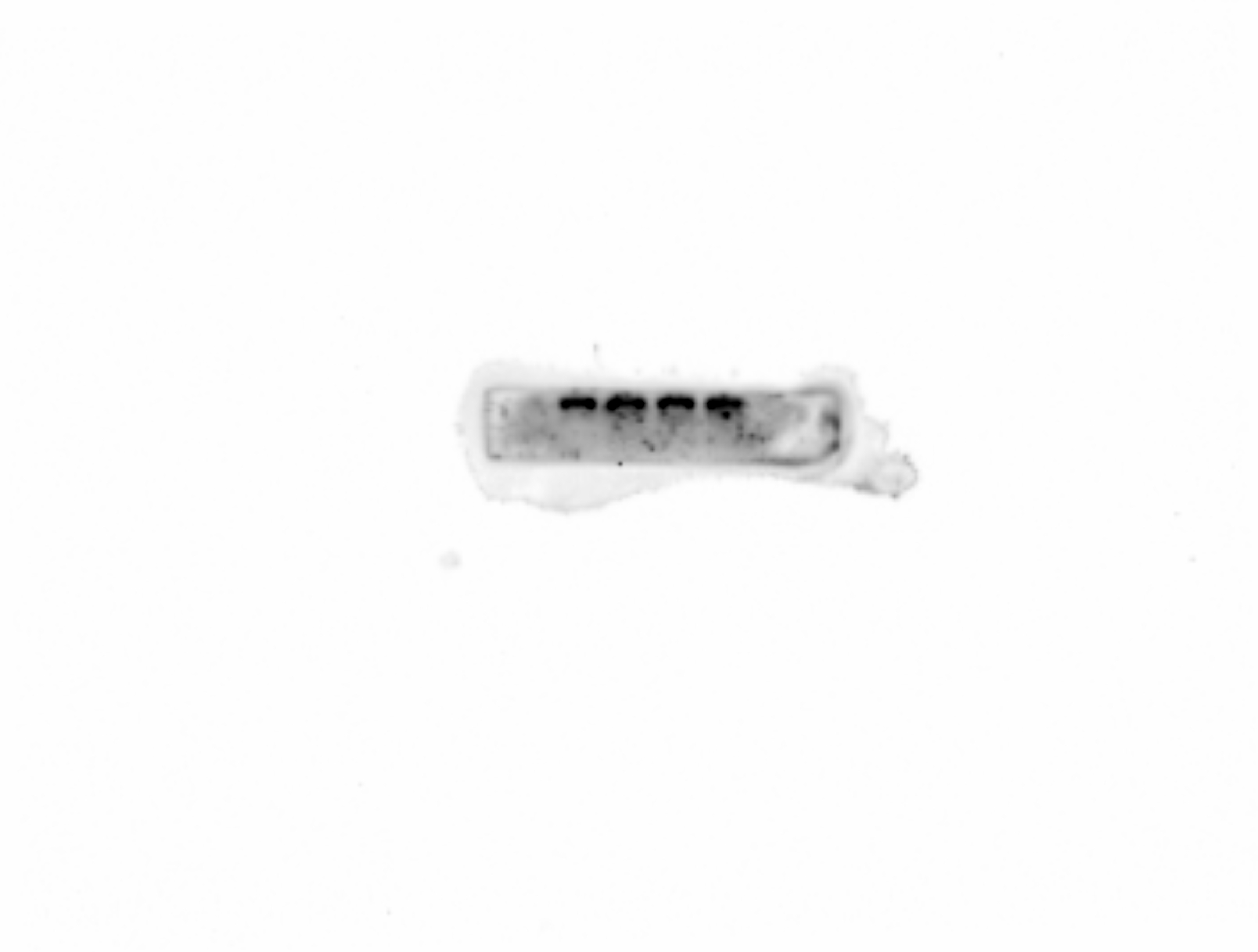


MGC803 p-STAT3(Y705) MGC803 E-cad


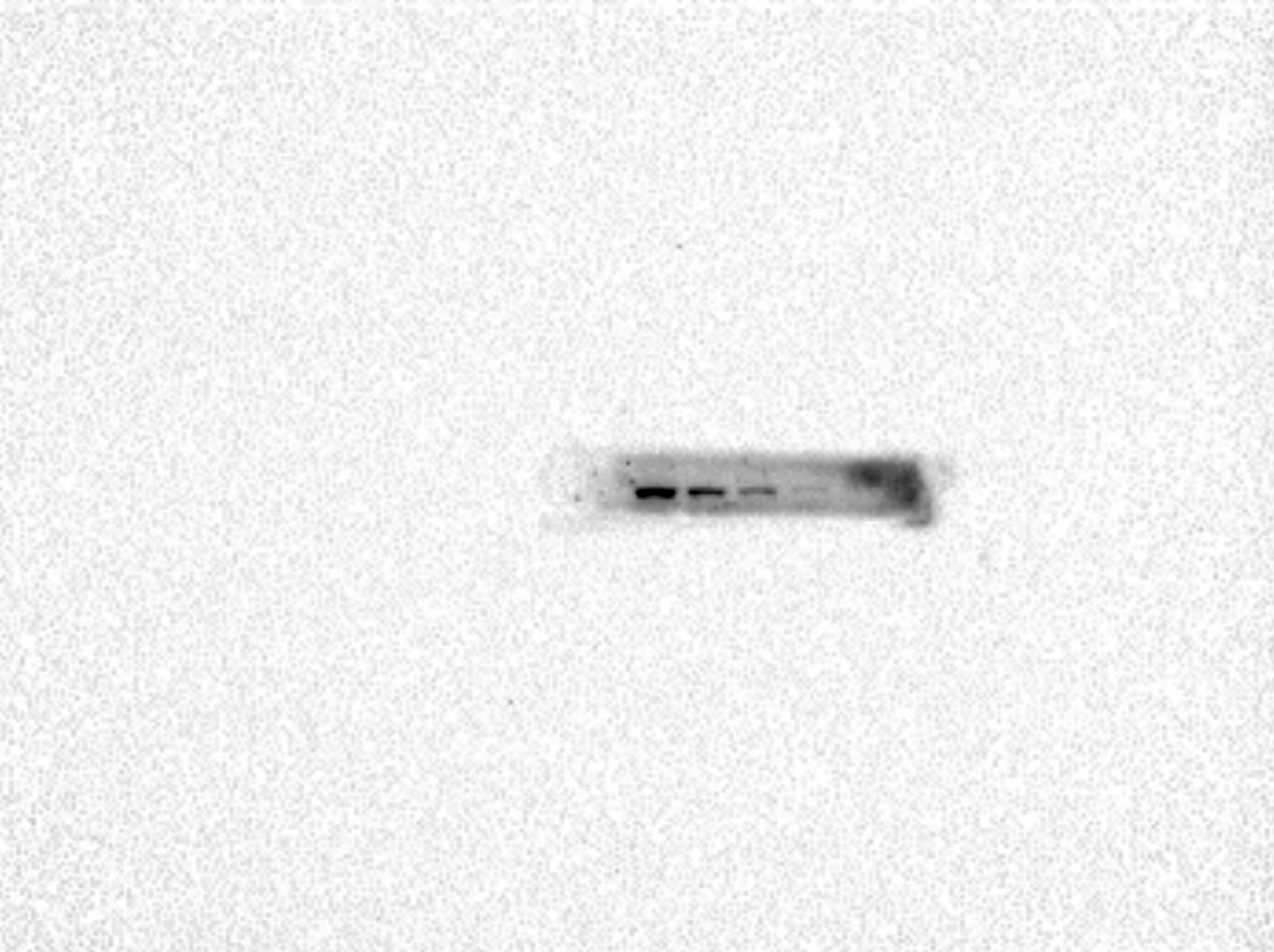

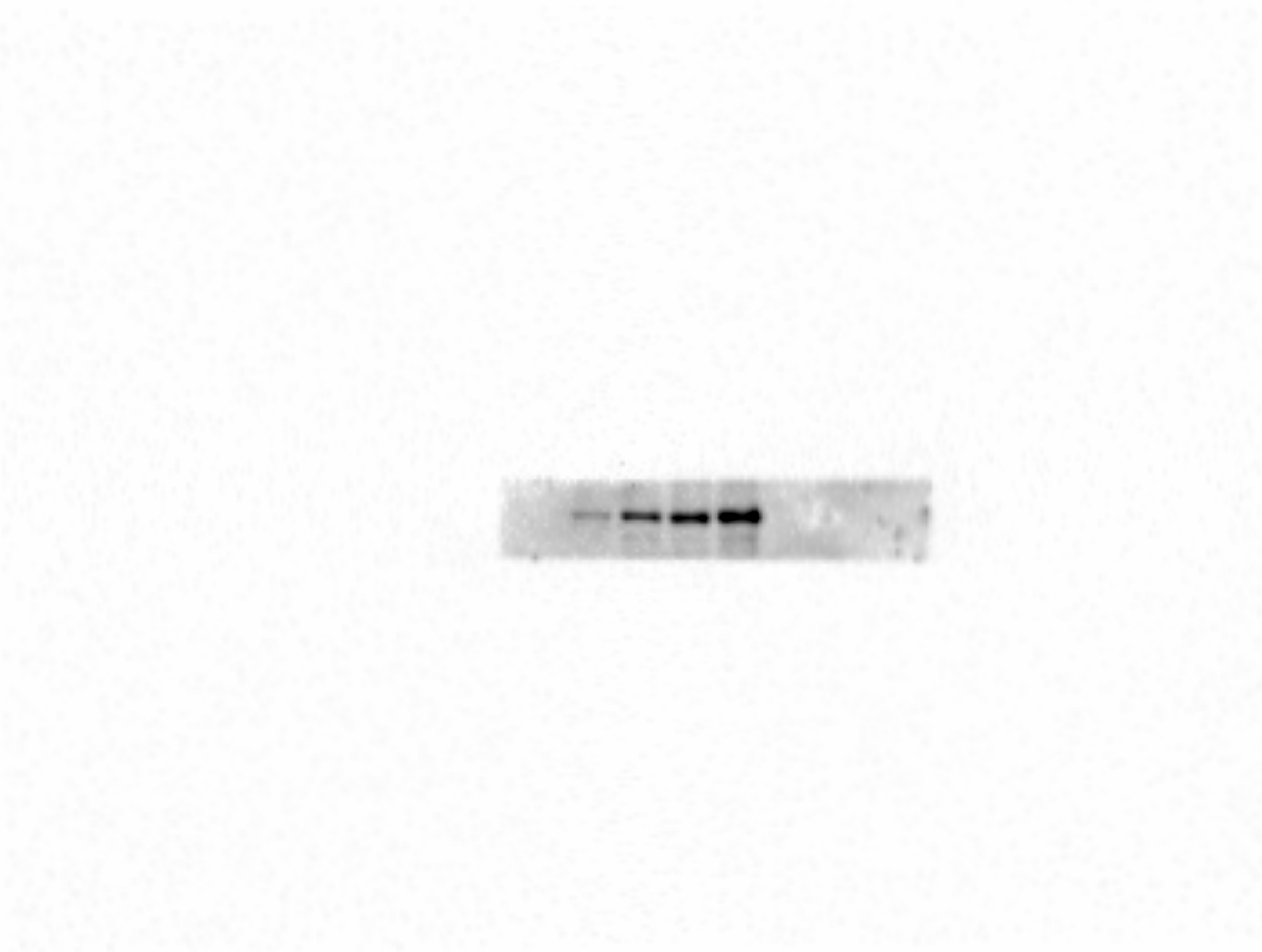


MGC803 N-cad MGC803 Vimentin


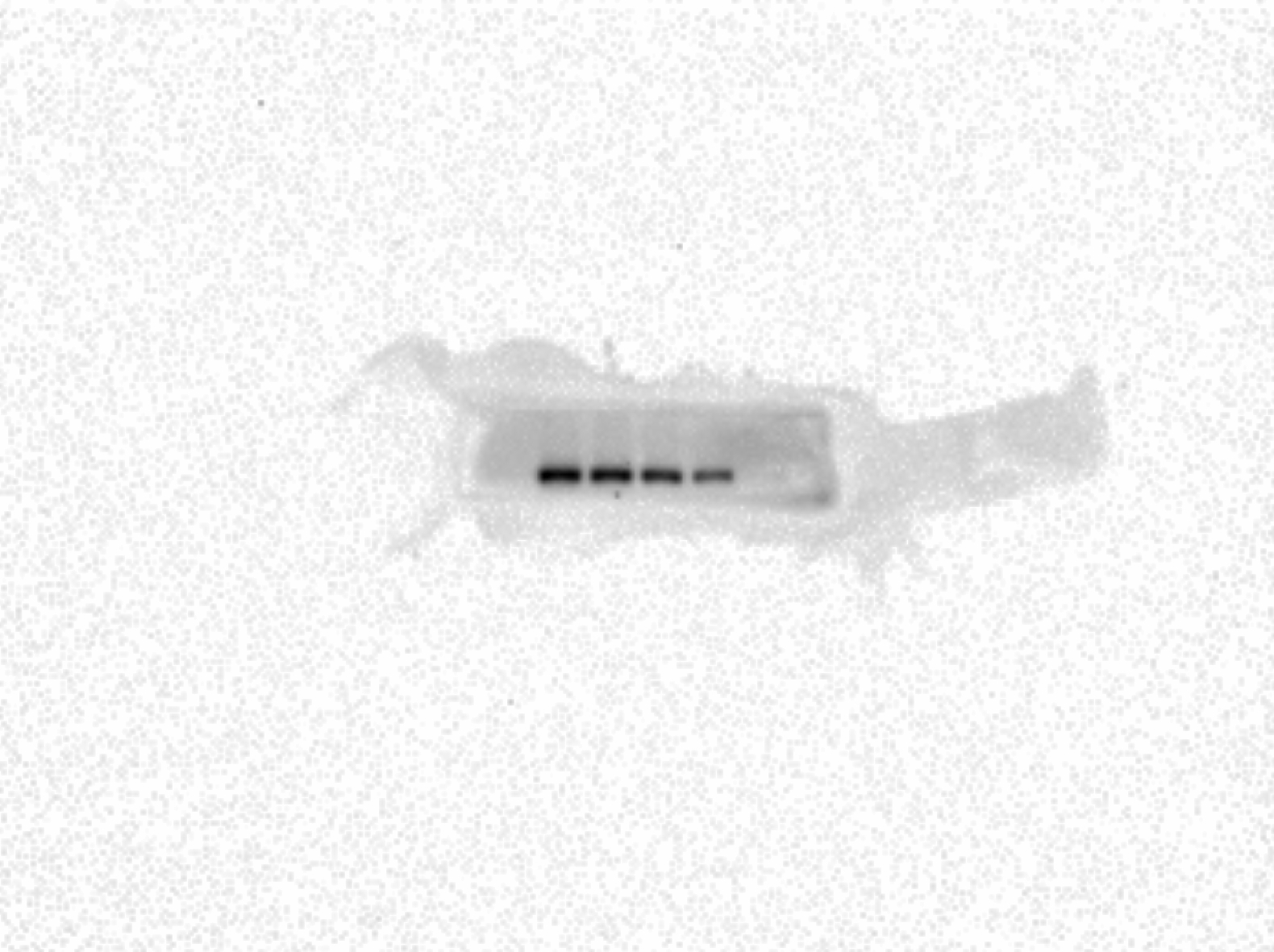

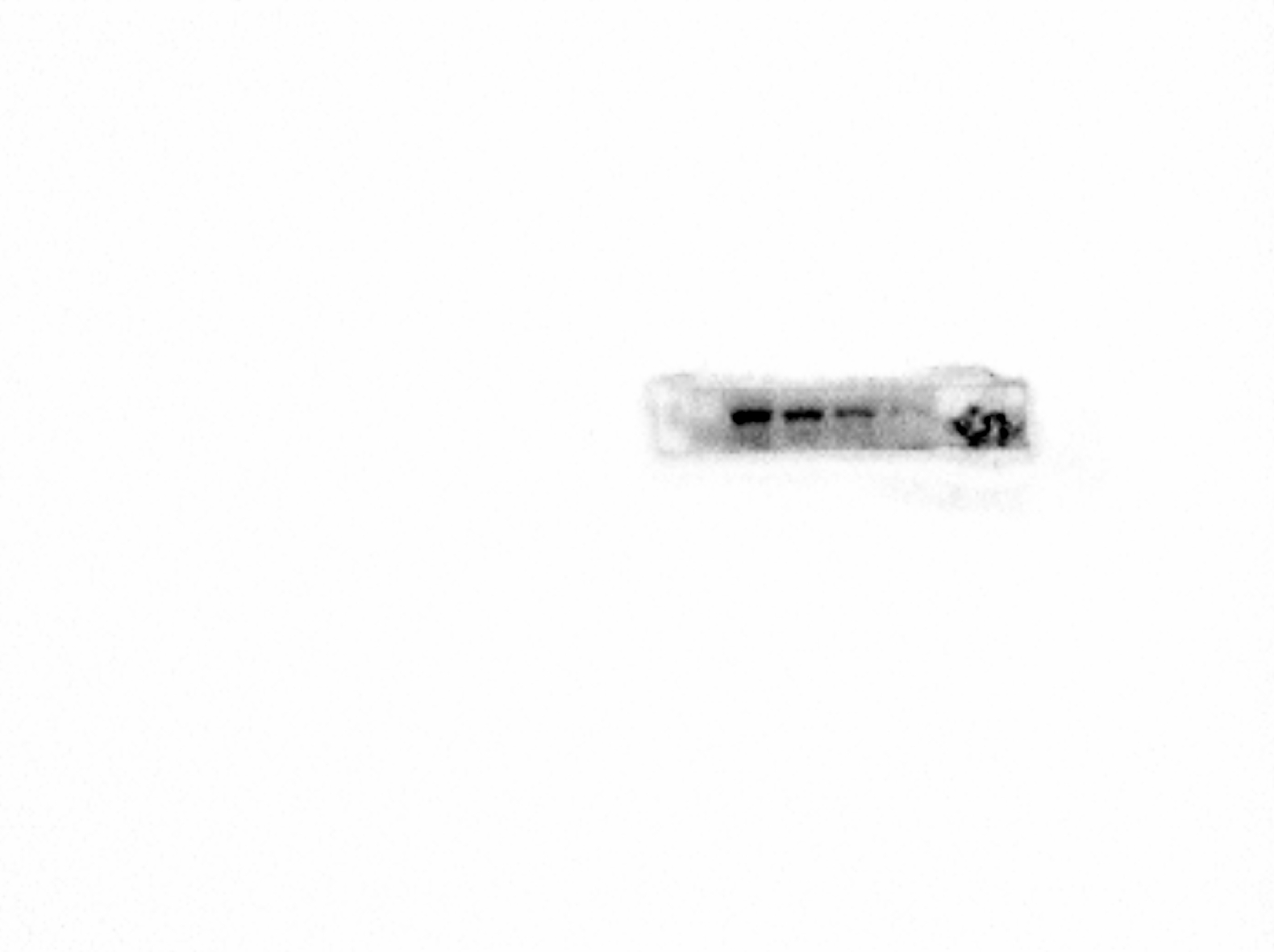


MGC803 GAPDH


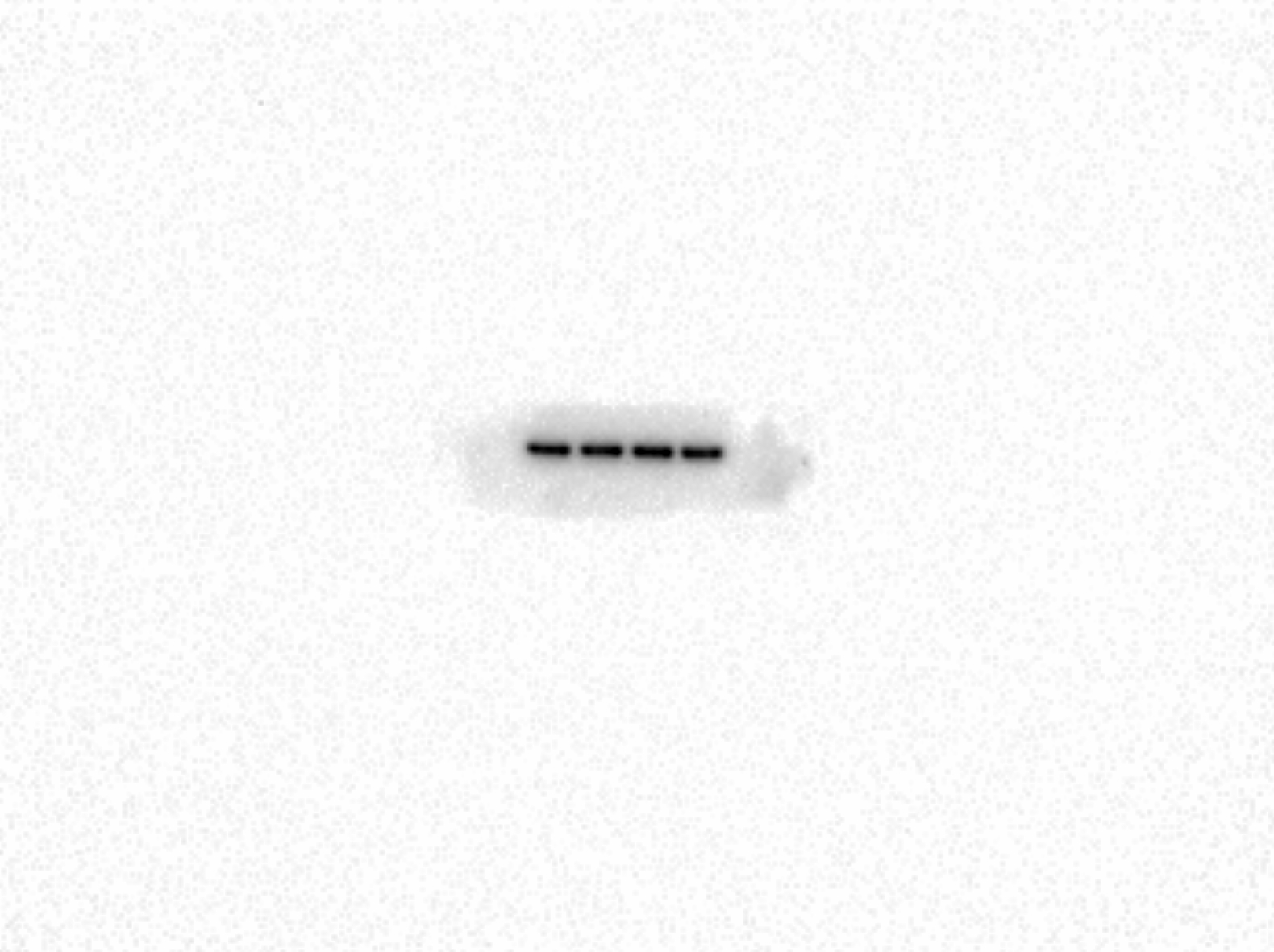


MKN74 Syntenin MKN74 STAT3


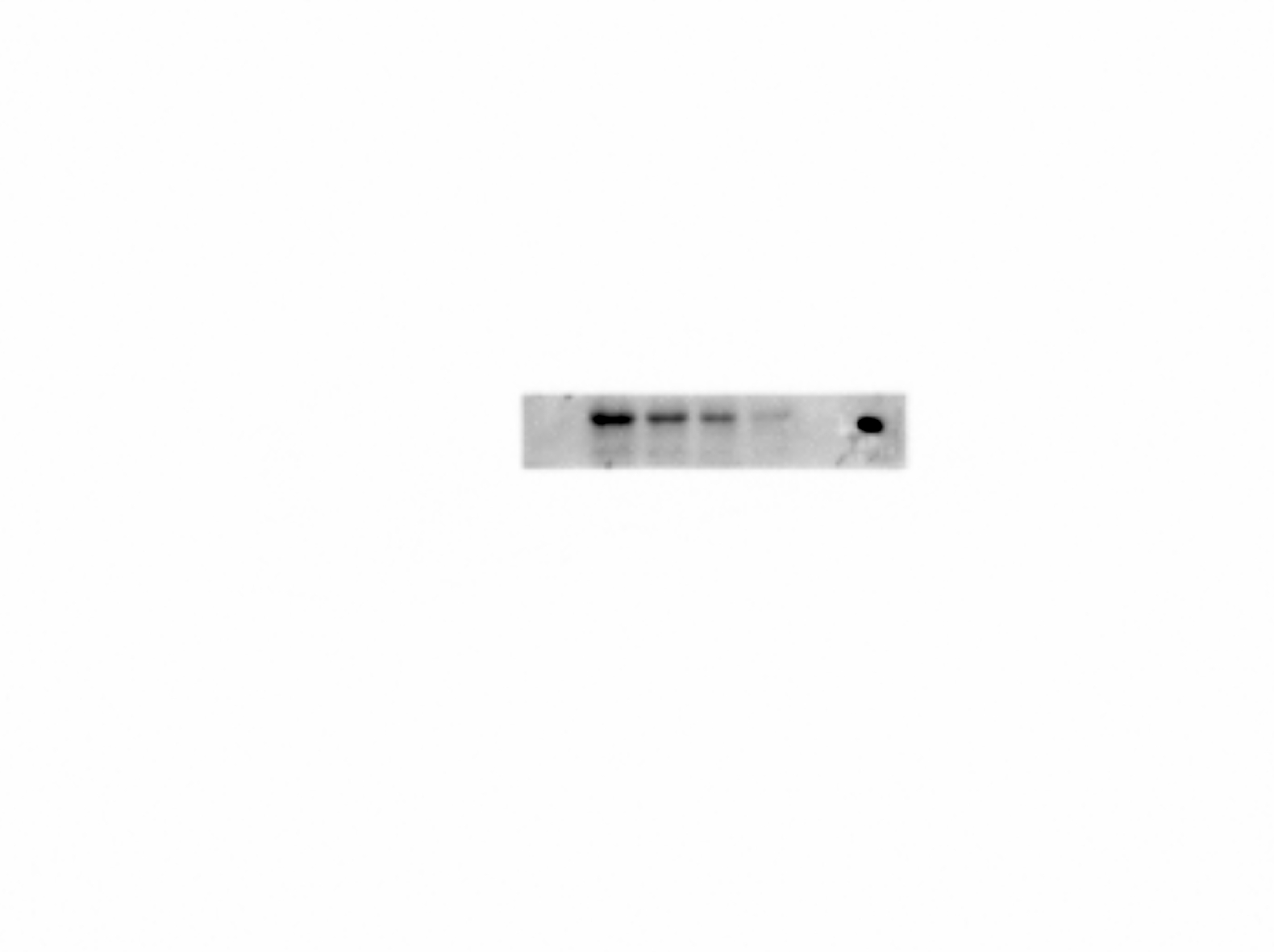

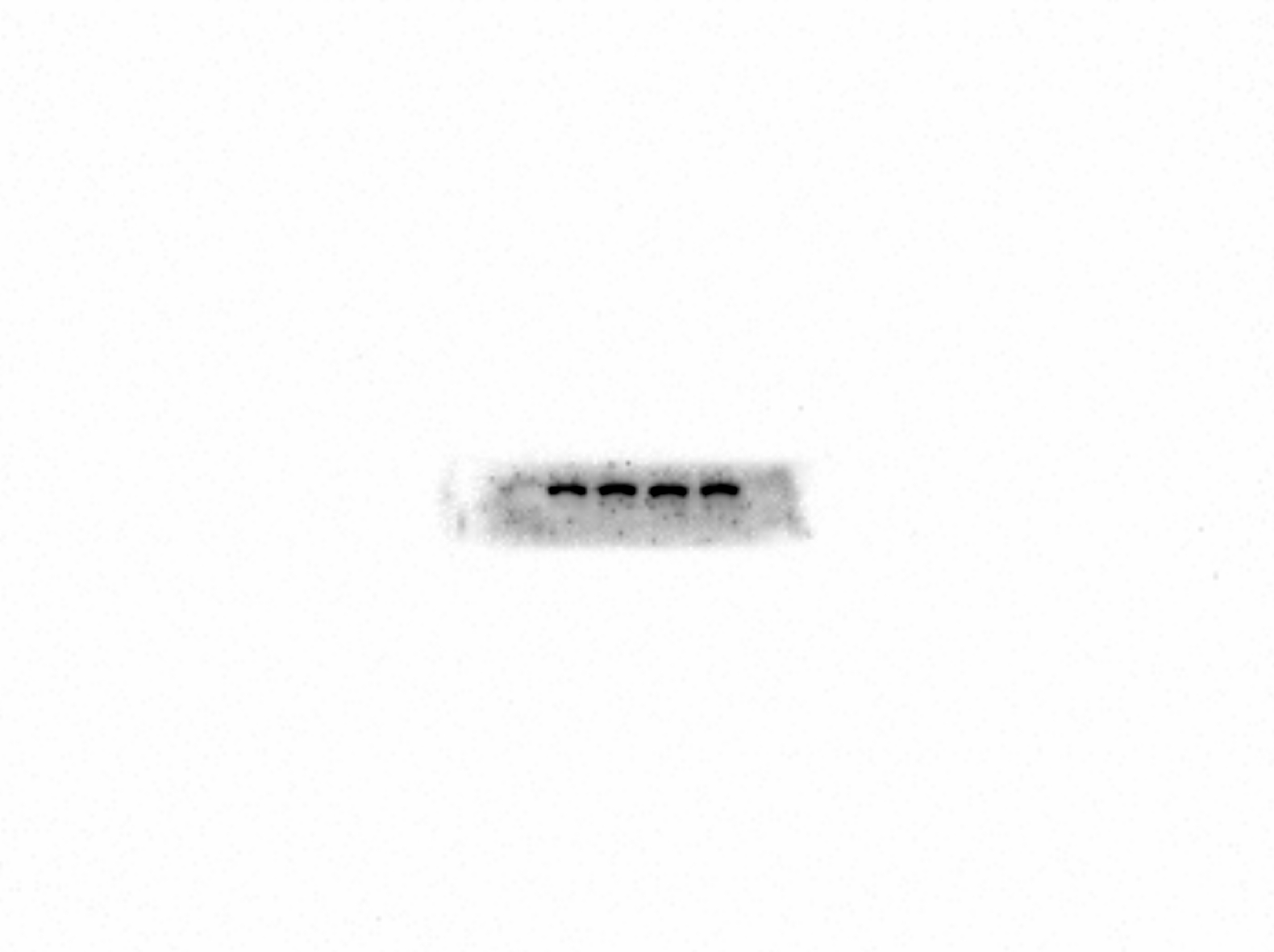


MKN74 p-STAT3(Y705) MKN74 E-cad


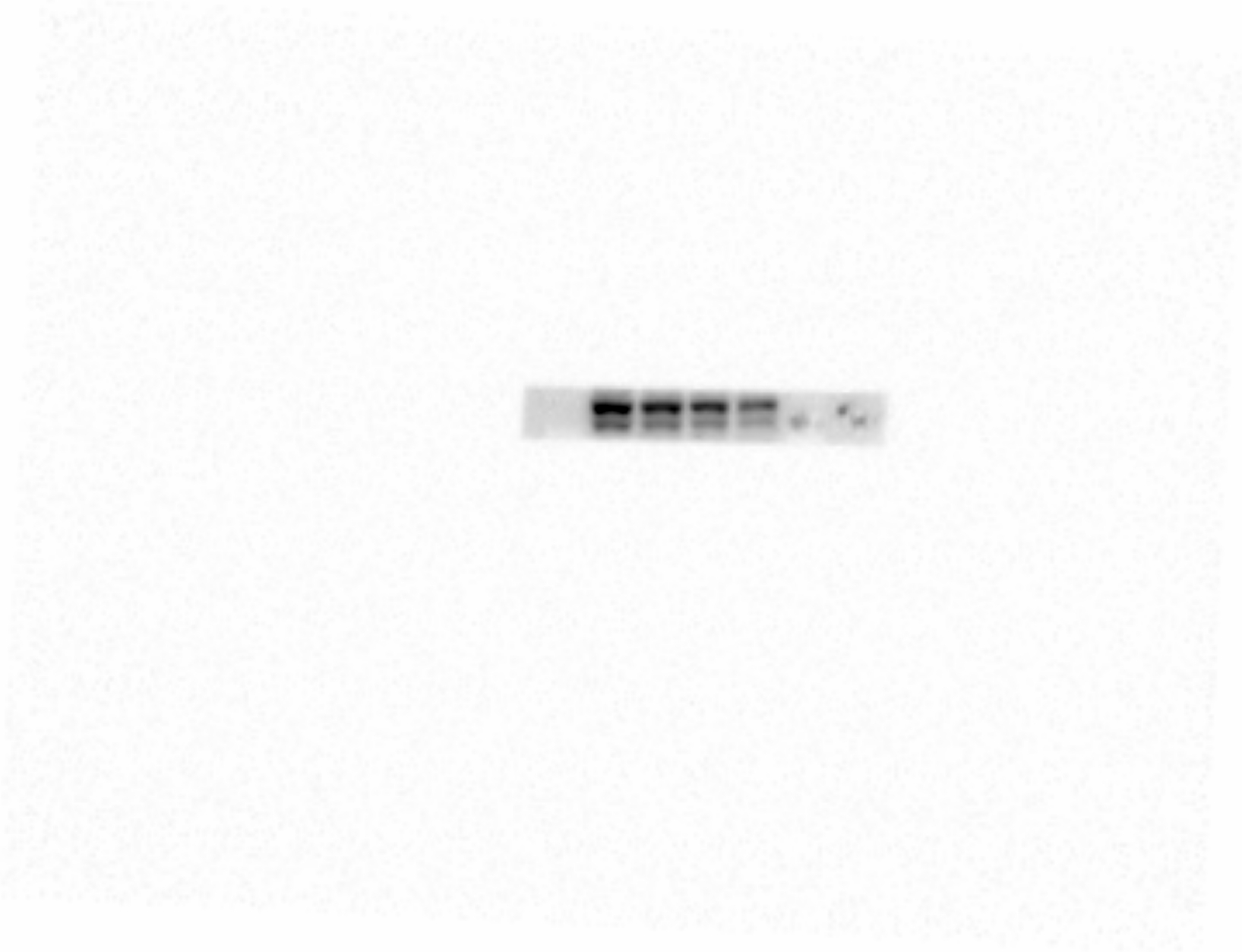

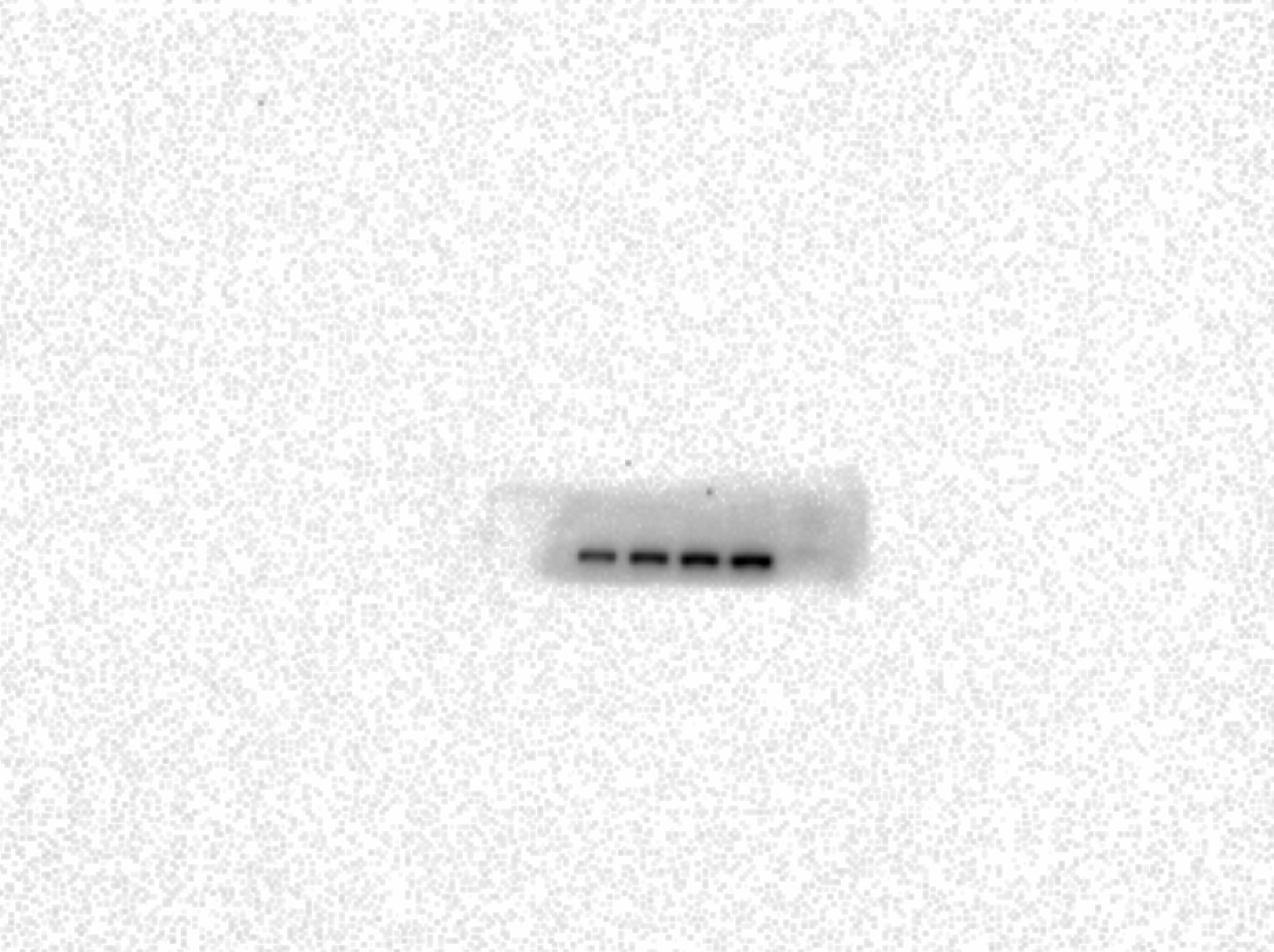


MKN74 N-cad MKN74 Vimentin


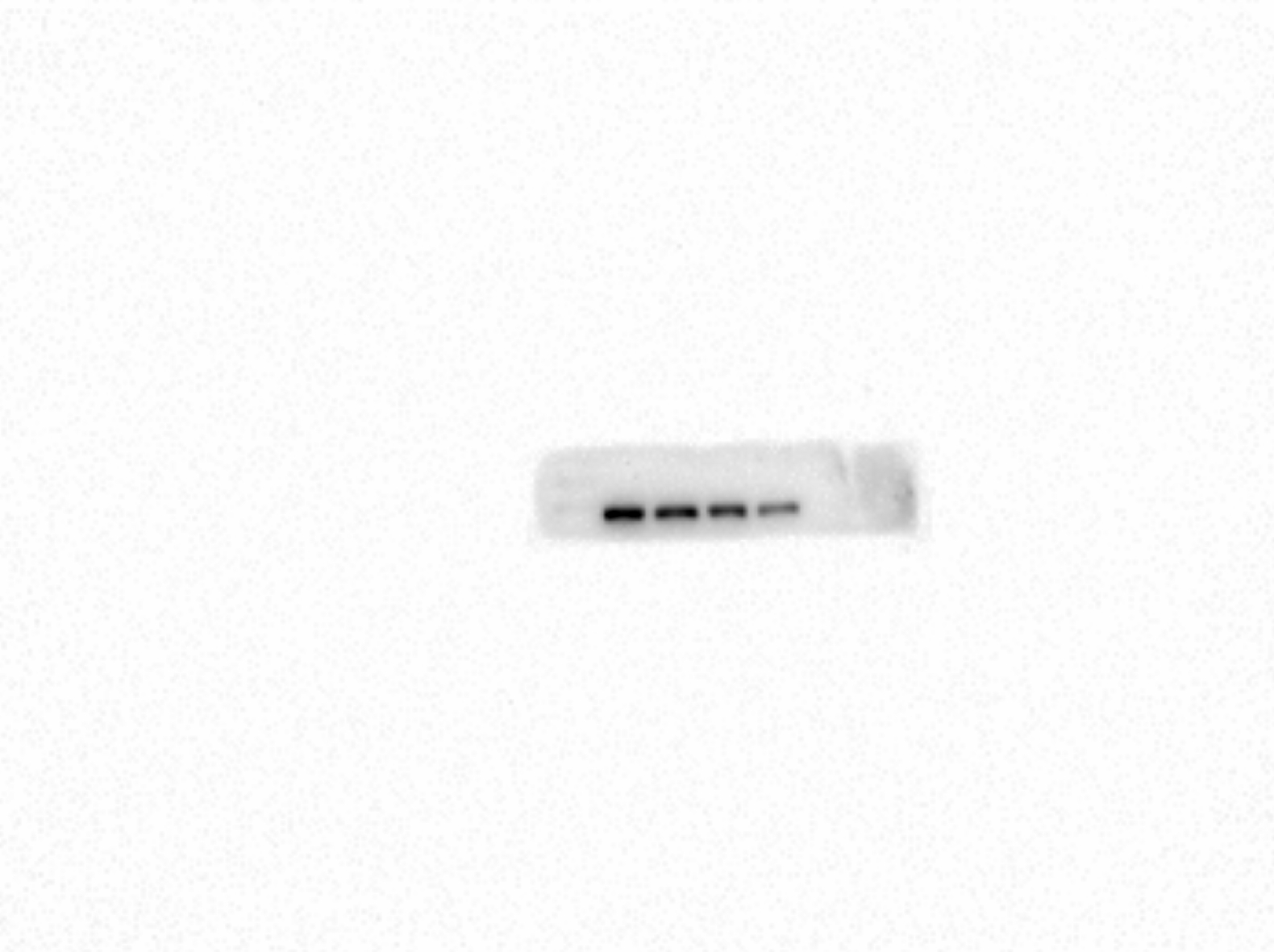

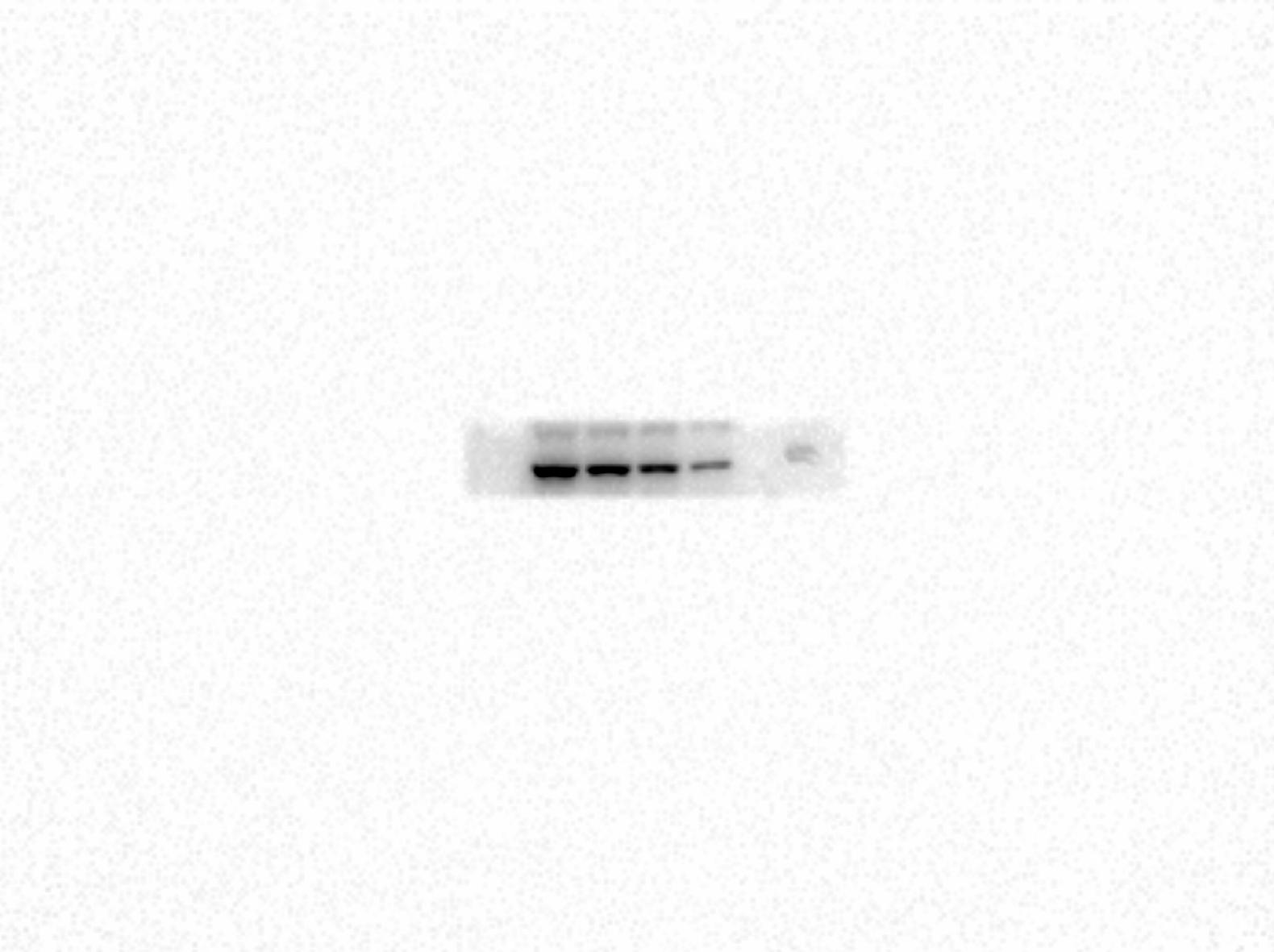


MKN74 GAPDH


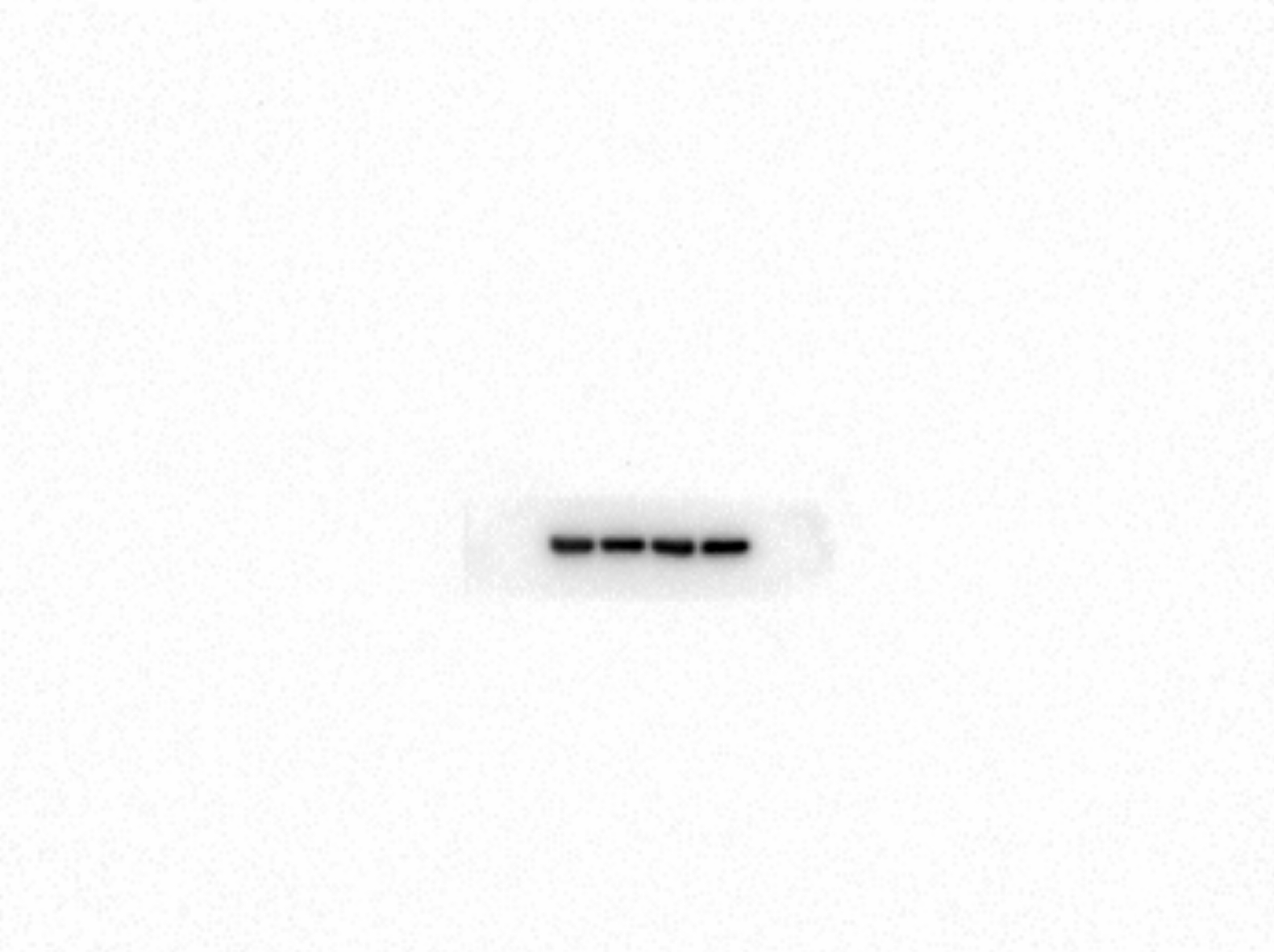


AZ-521 Syntenin AZ-521 STAT3


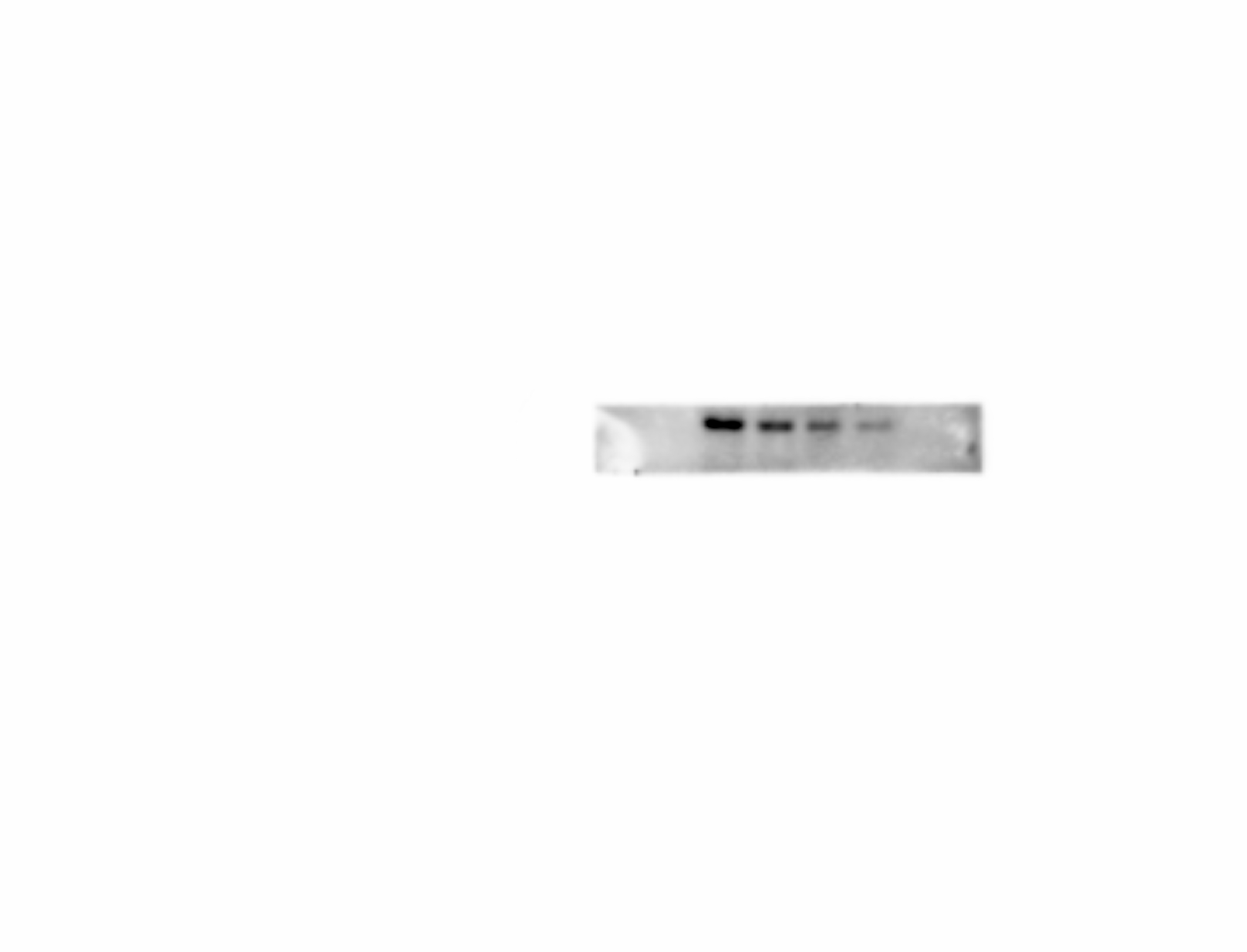

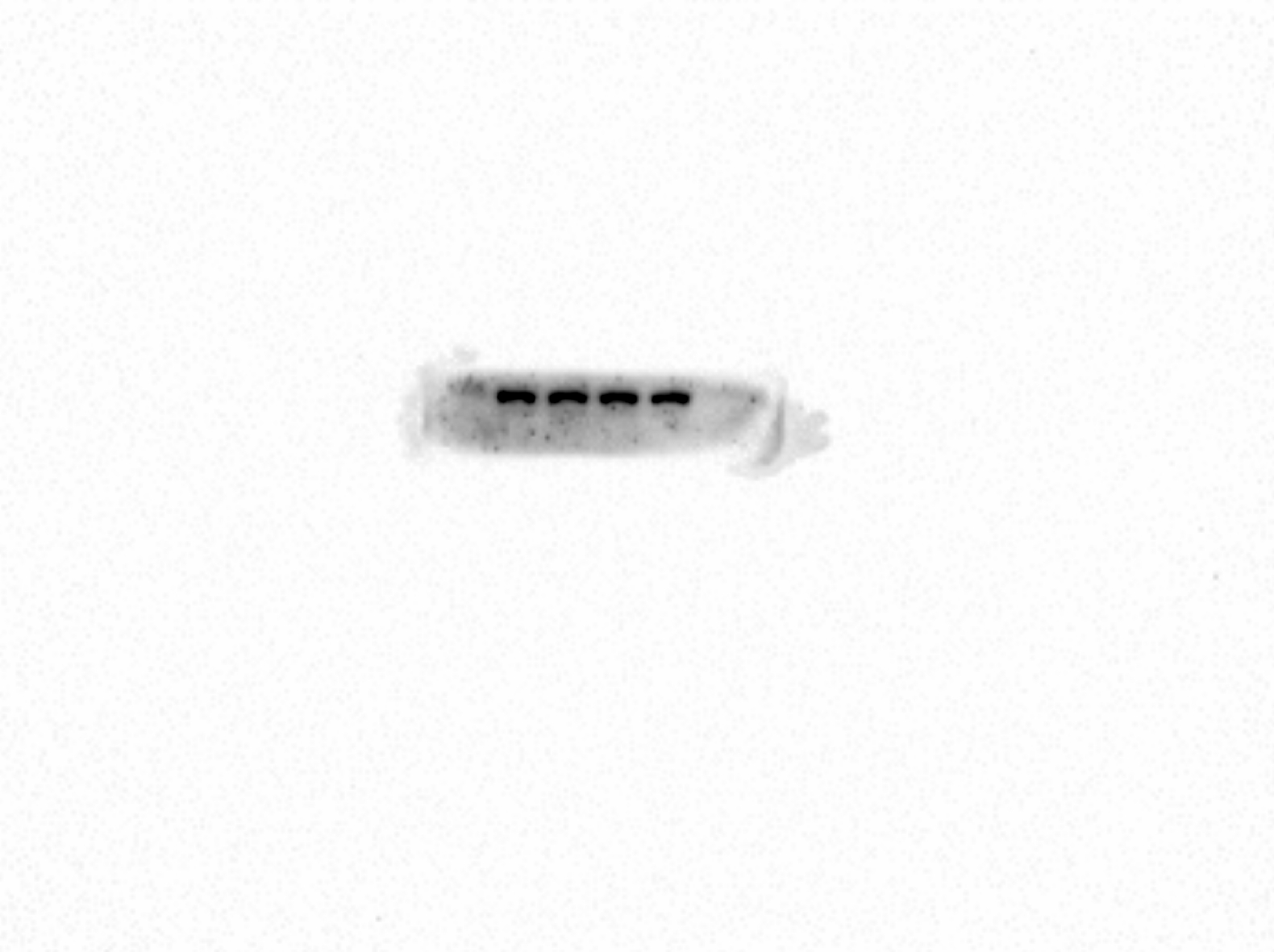


AZ-521 p-STAT3(Y705) AZ-521 E-cad


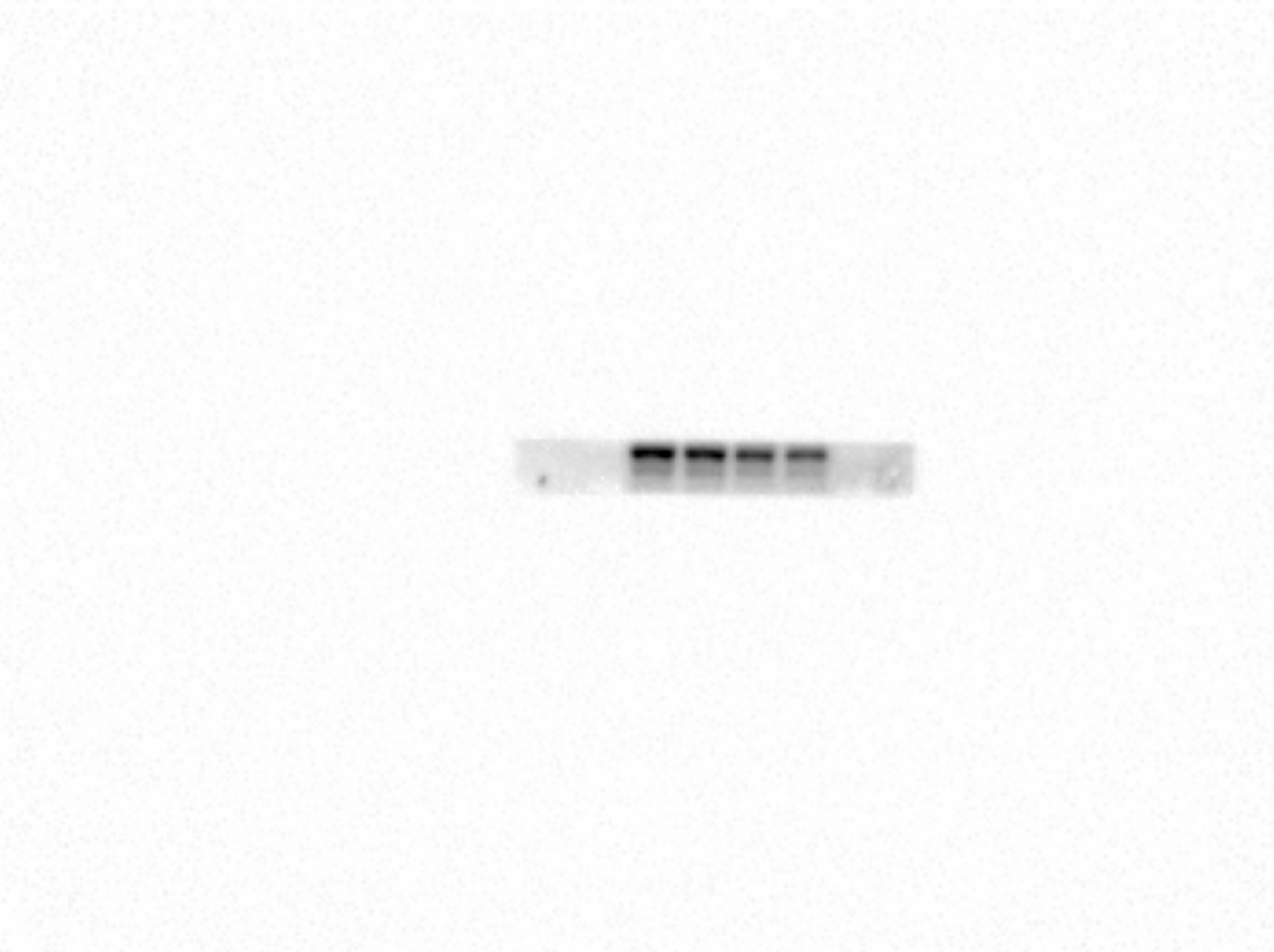

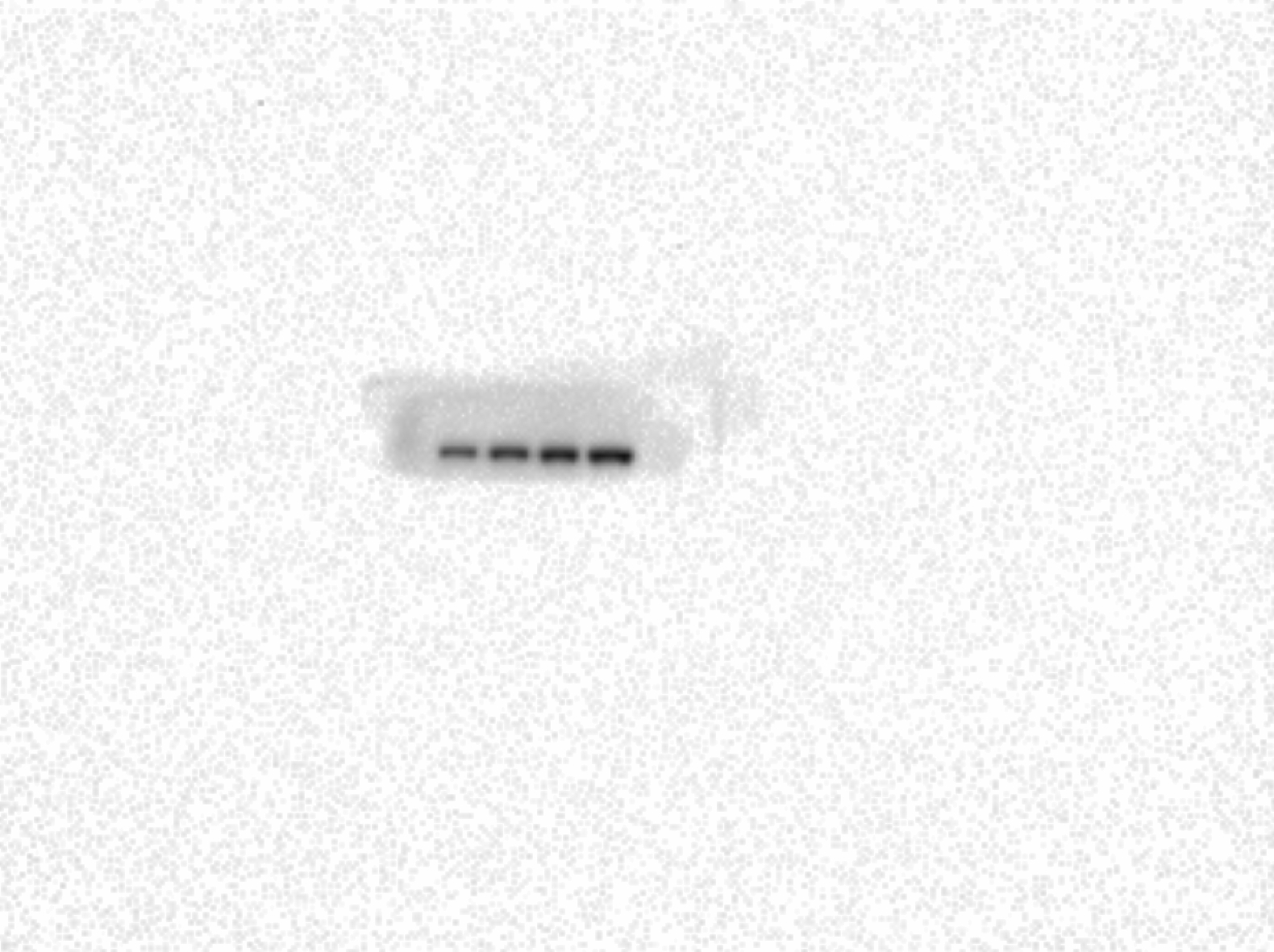


AZ-521 N-cad AZ-521 Vimentin


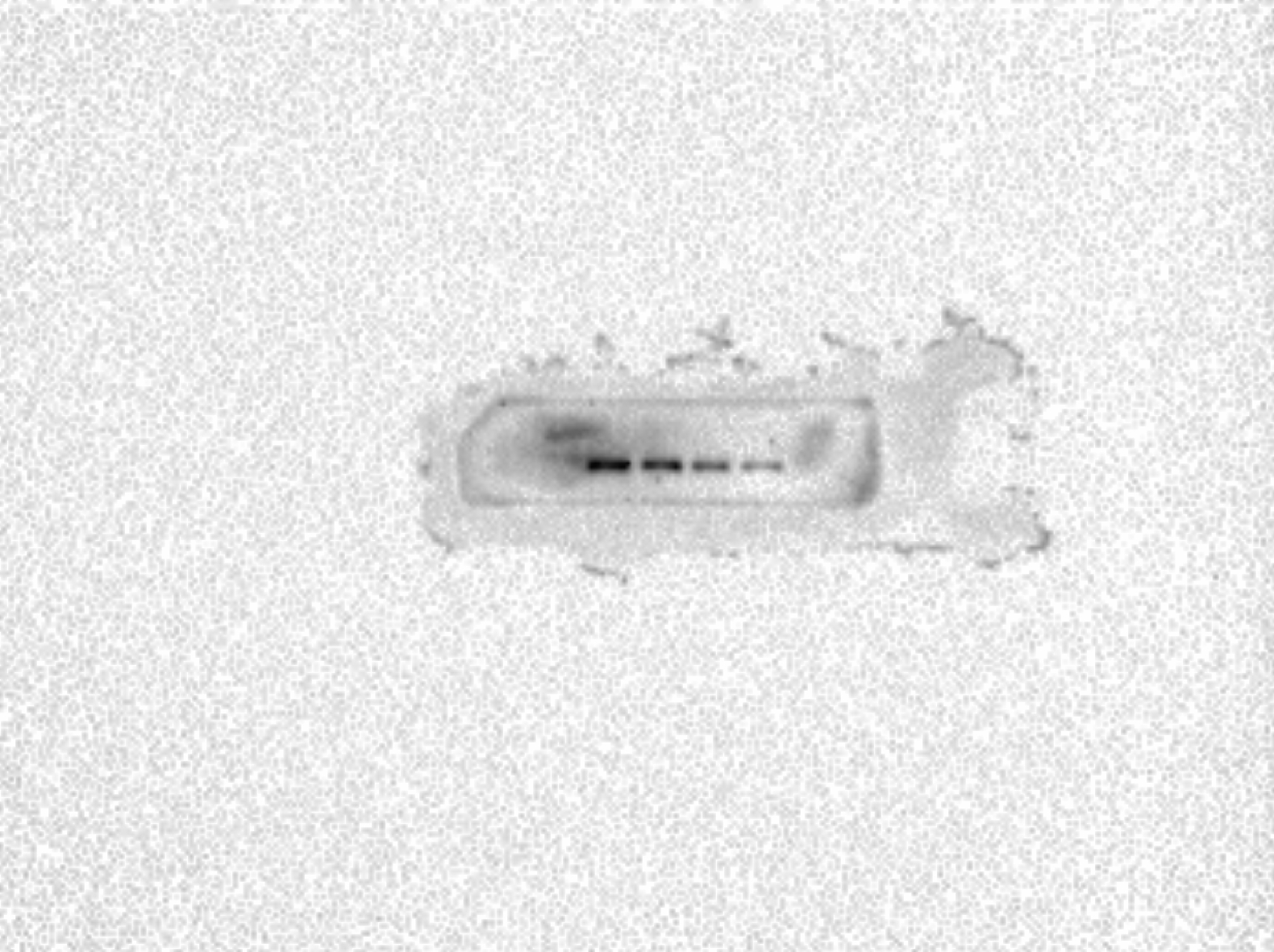

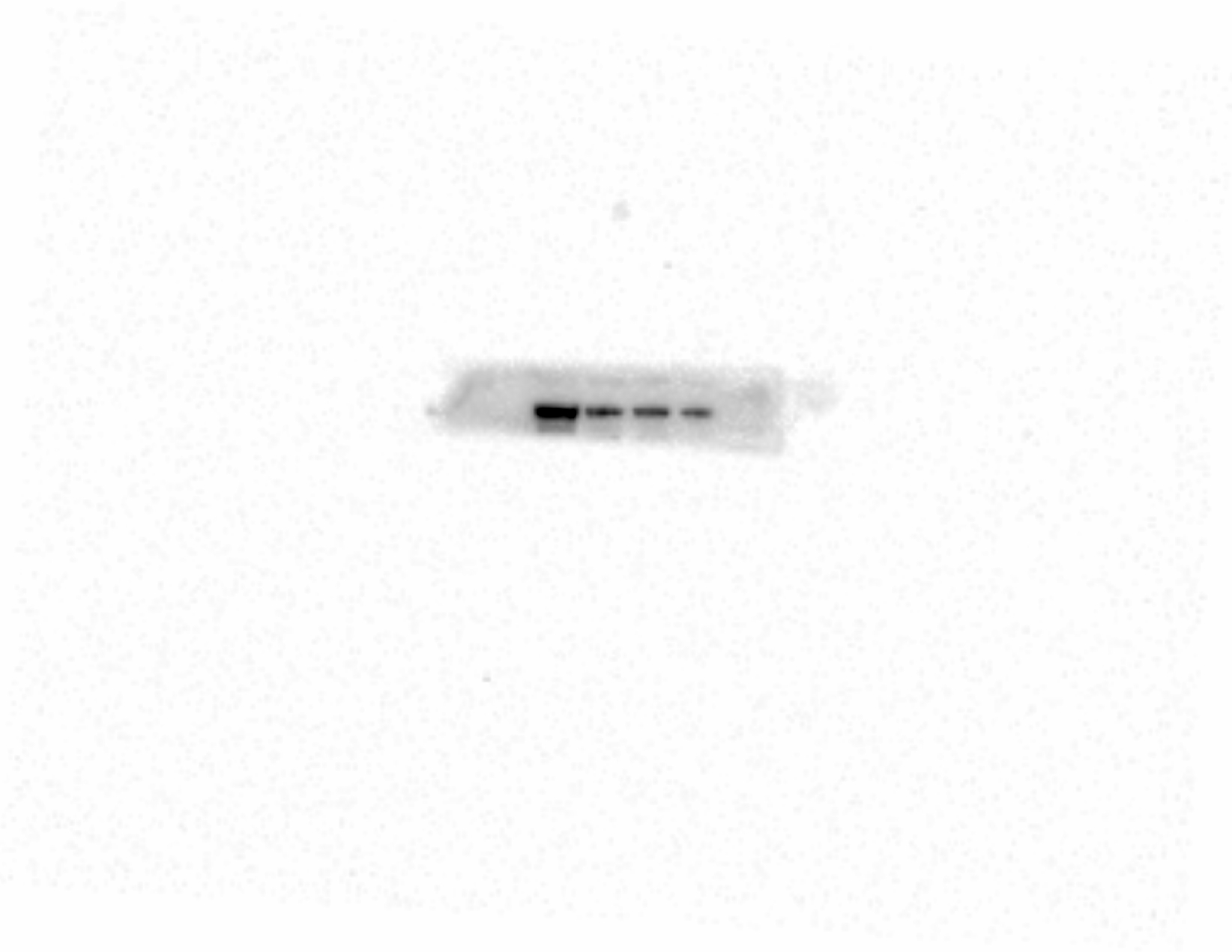


AZ-521 GAPDH


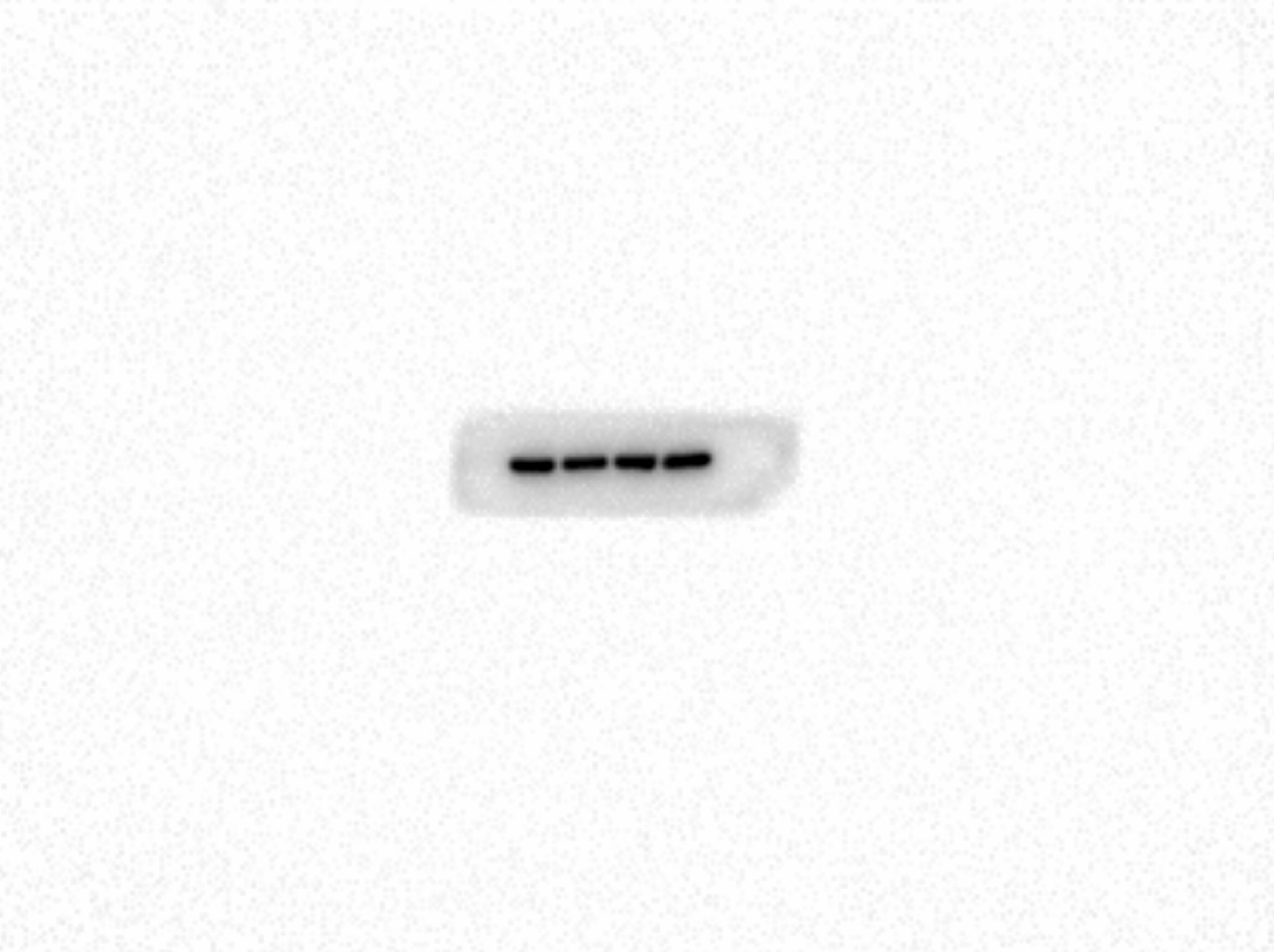


MKN28 Syntenin MKN28 STAT3


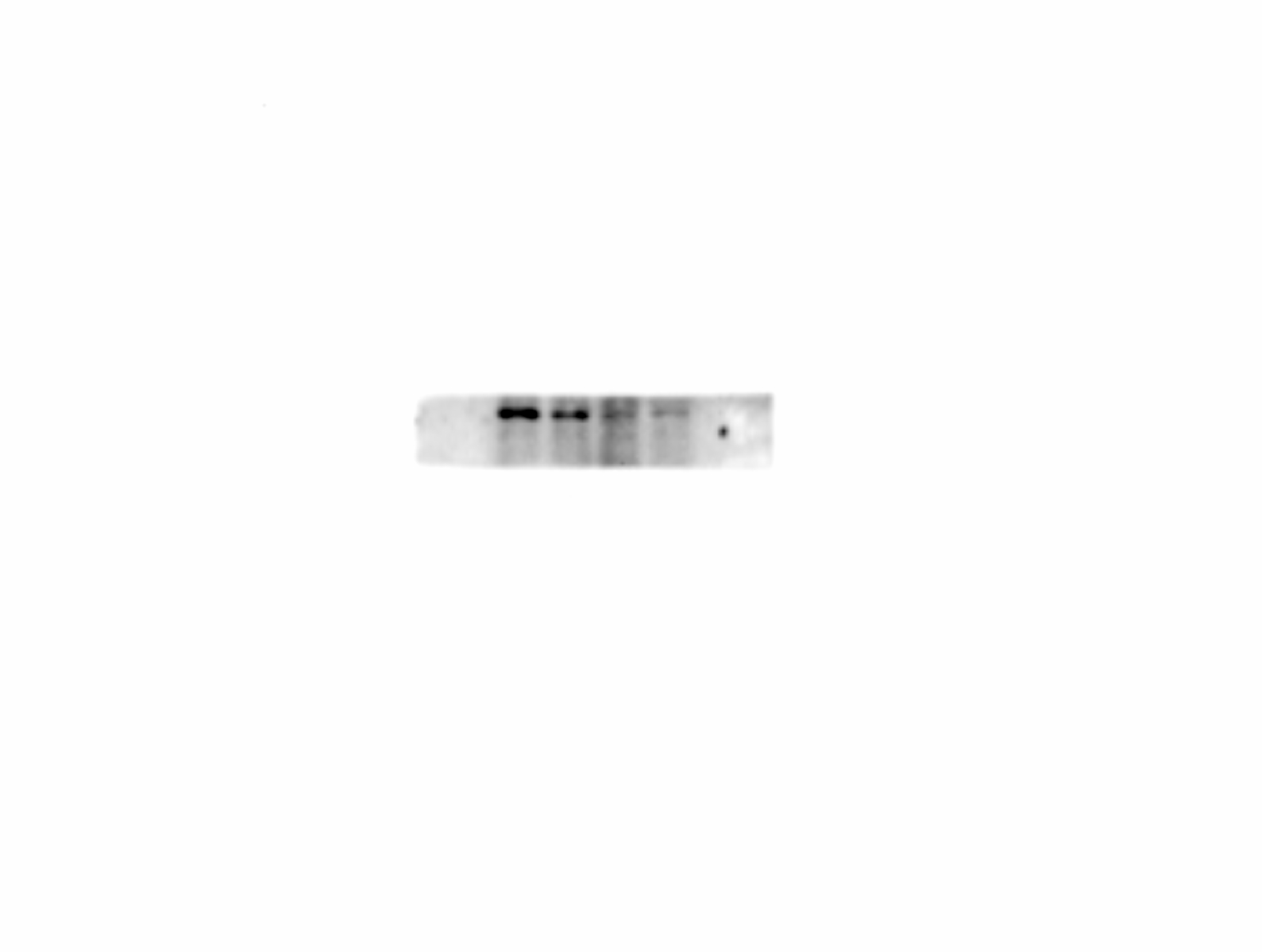

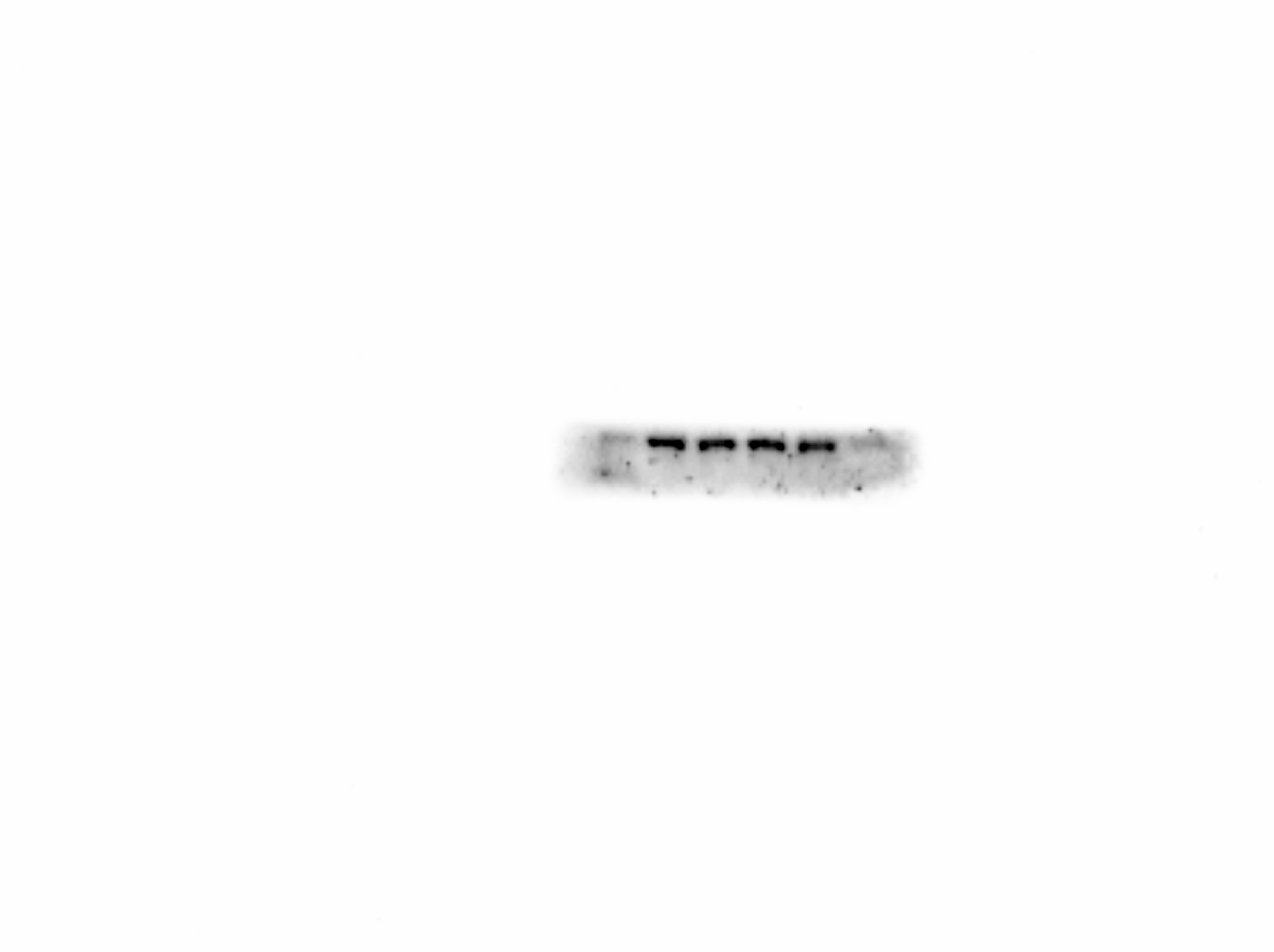


MKN28 p-STAT3(Y705) MKN28 E-cad


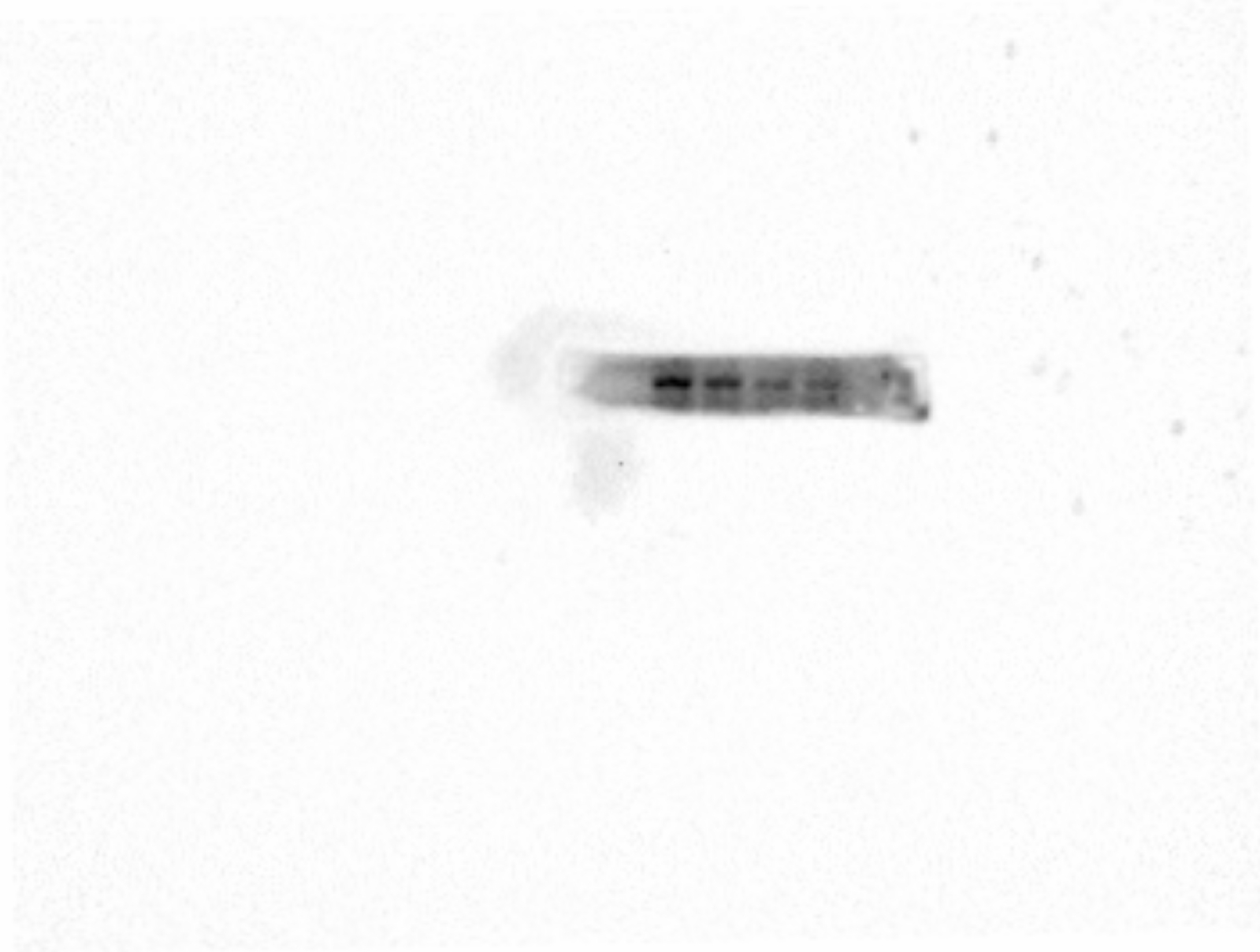

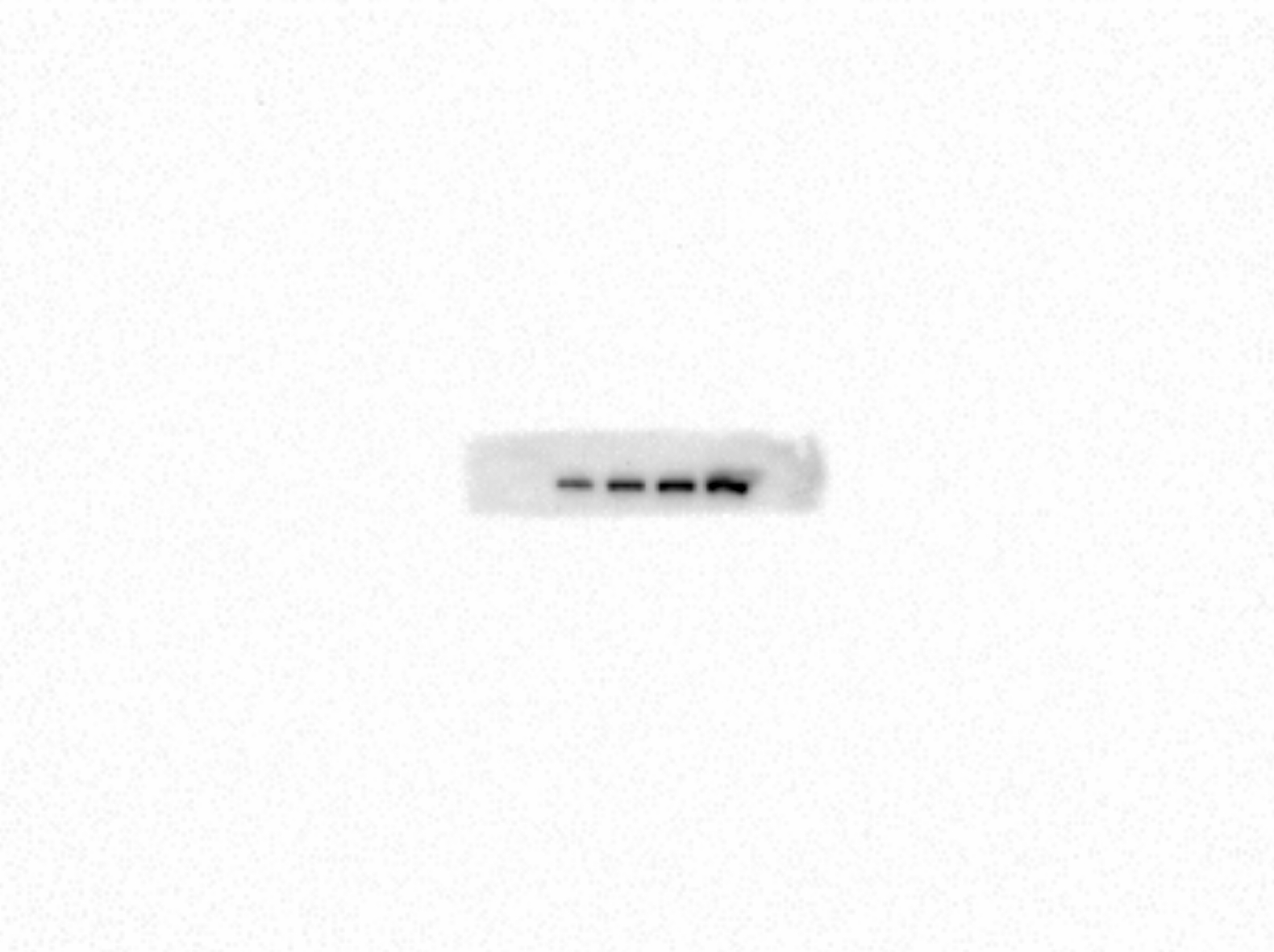


MKN28 N-cad MKN28 Vimentin


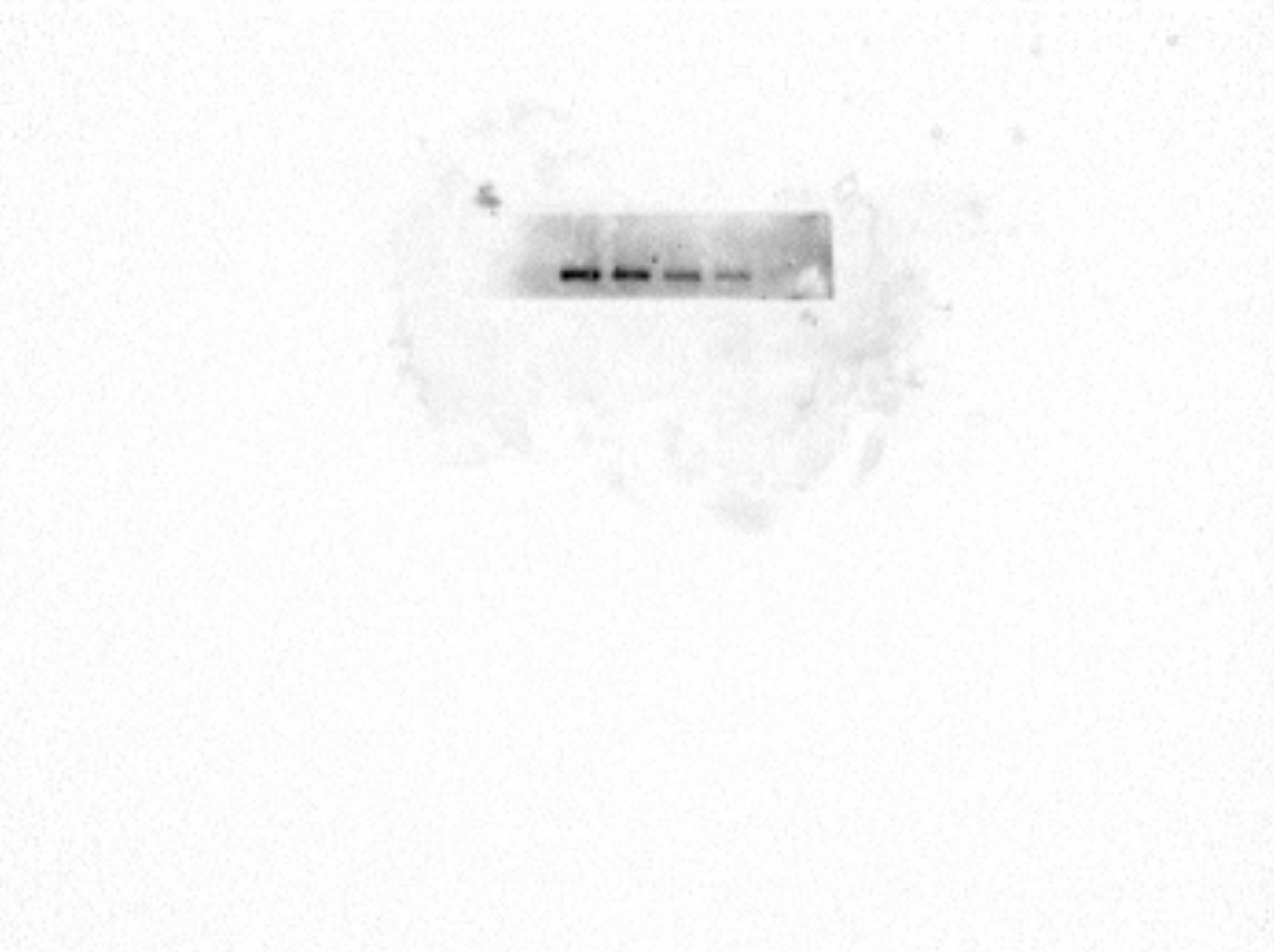

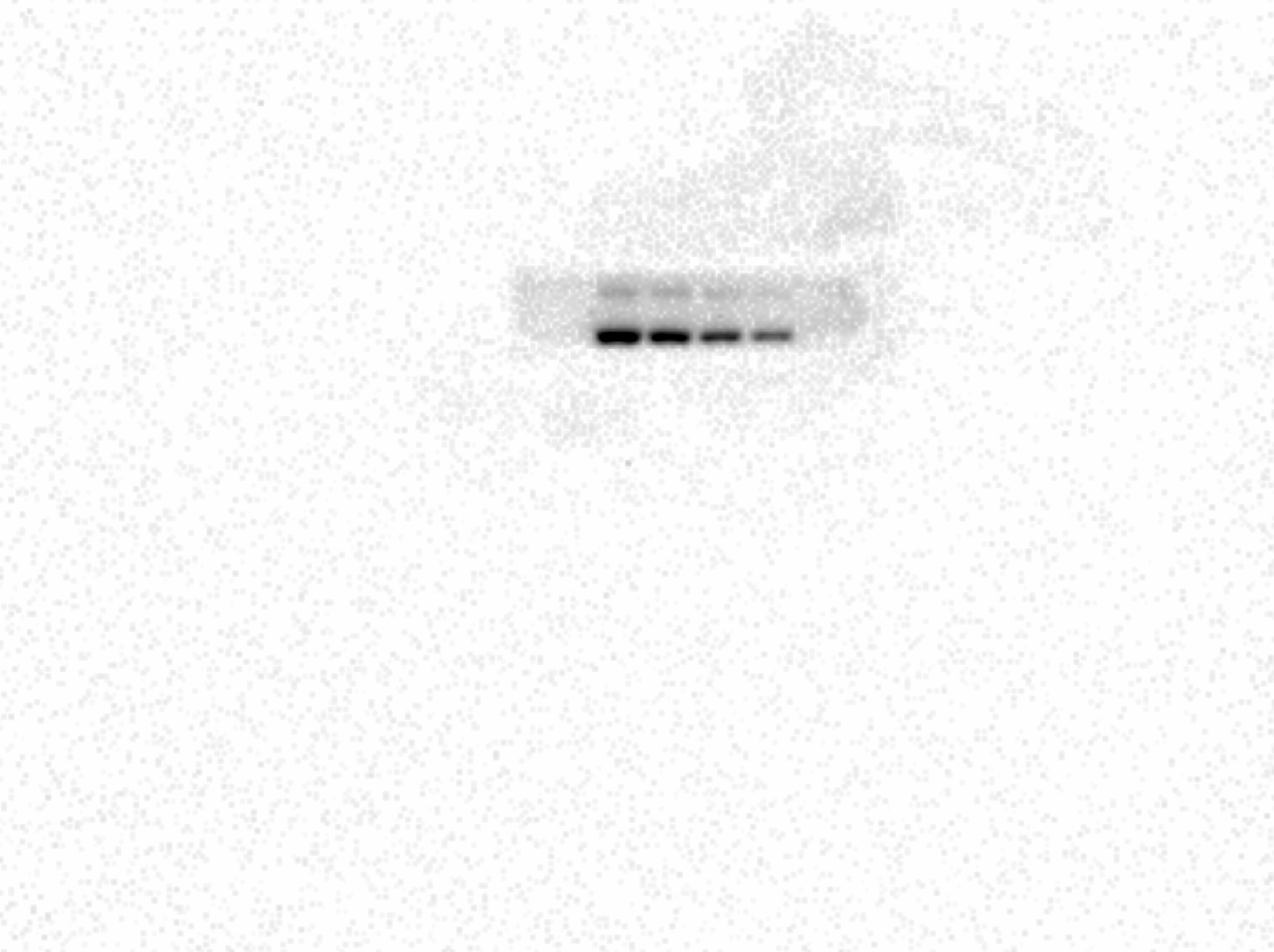


MKN28 GAPDH


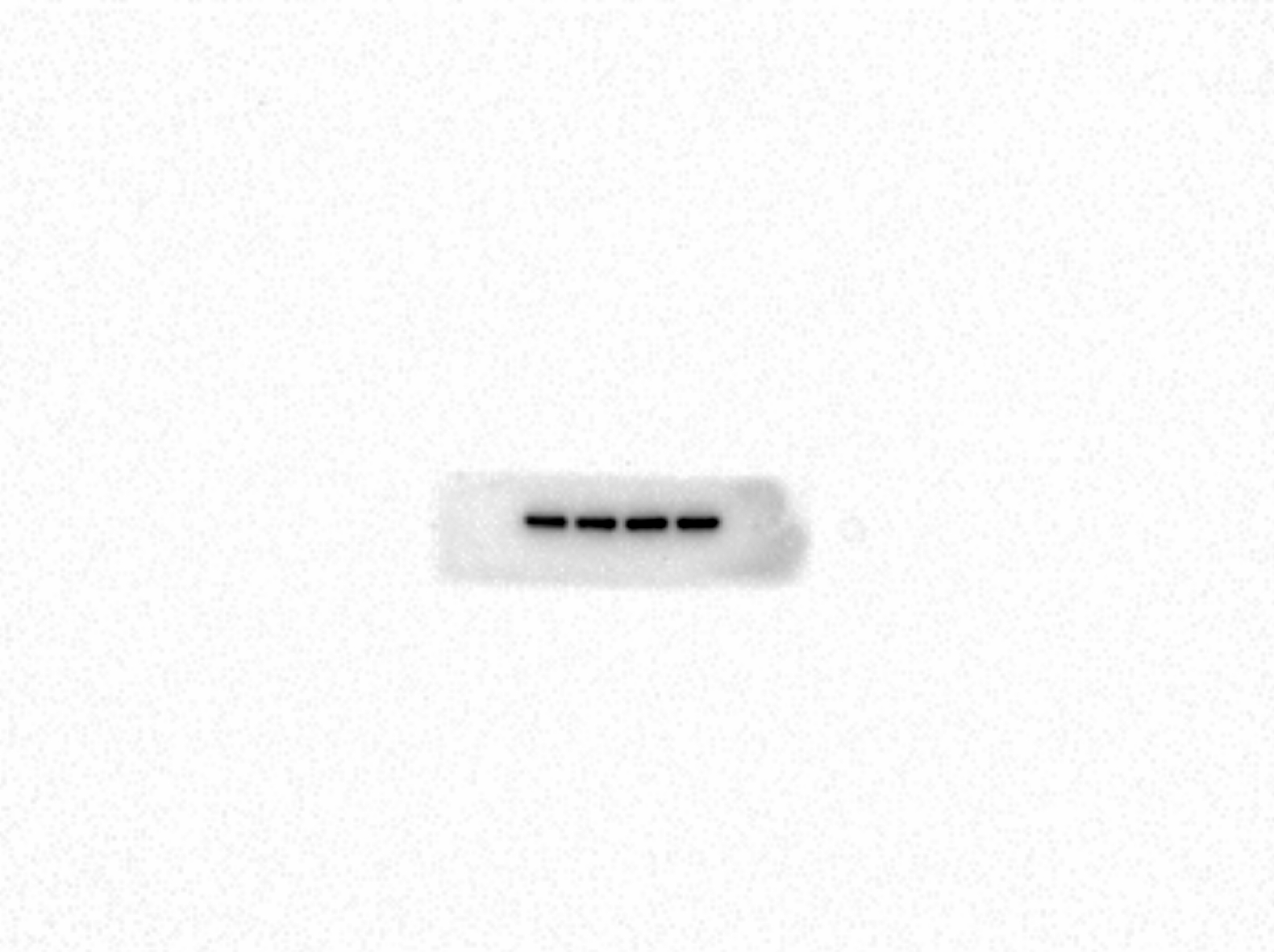


5.2 Uncut original image of Western Blot in figure 7.

MGC803(OE) Syntenin MGC803(OE) STAT3


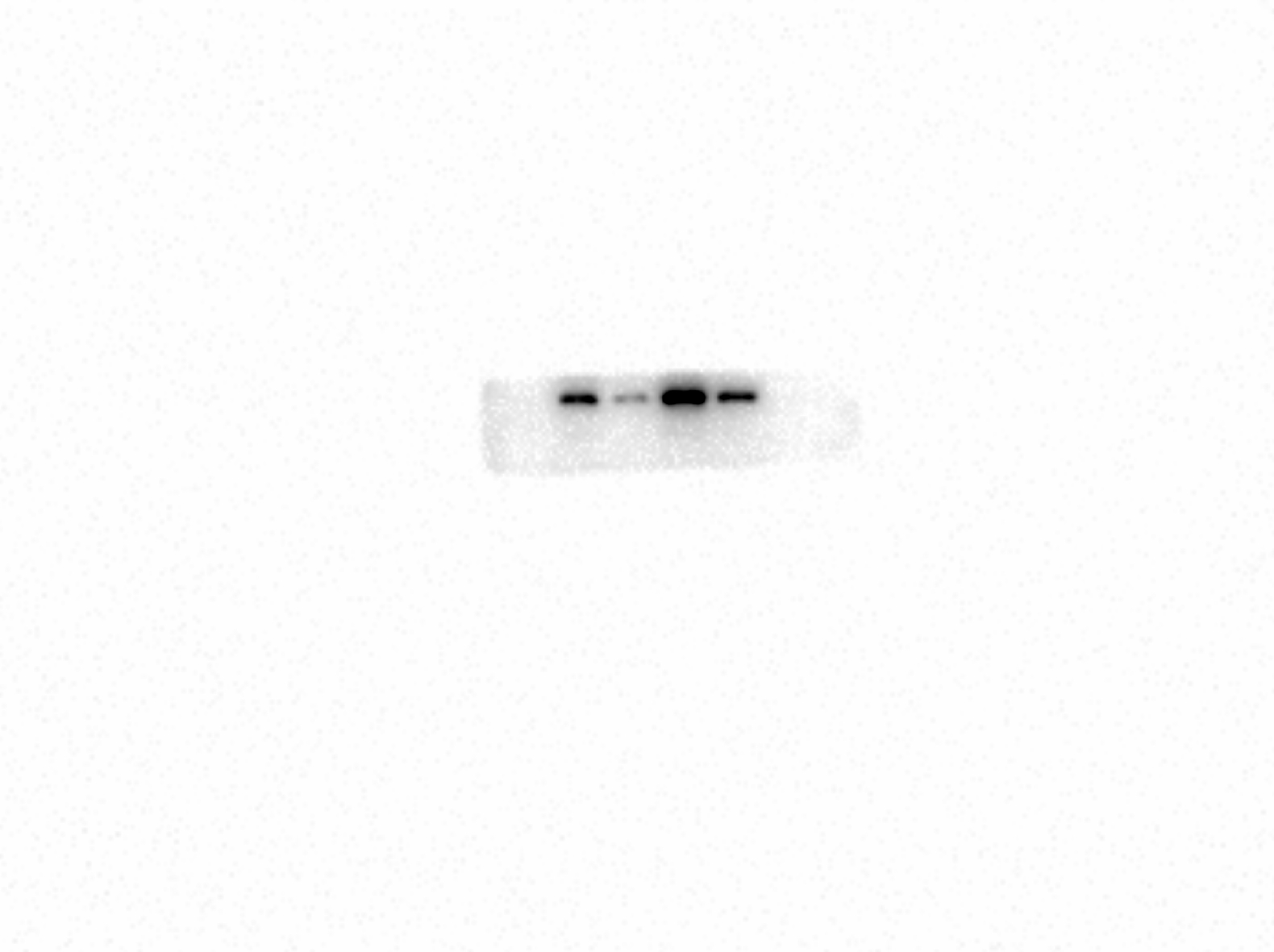

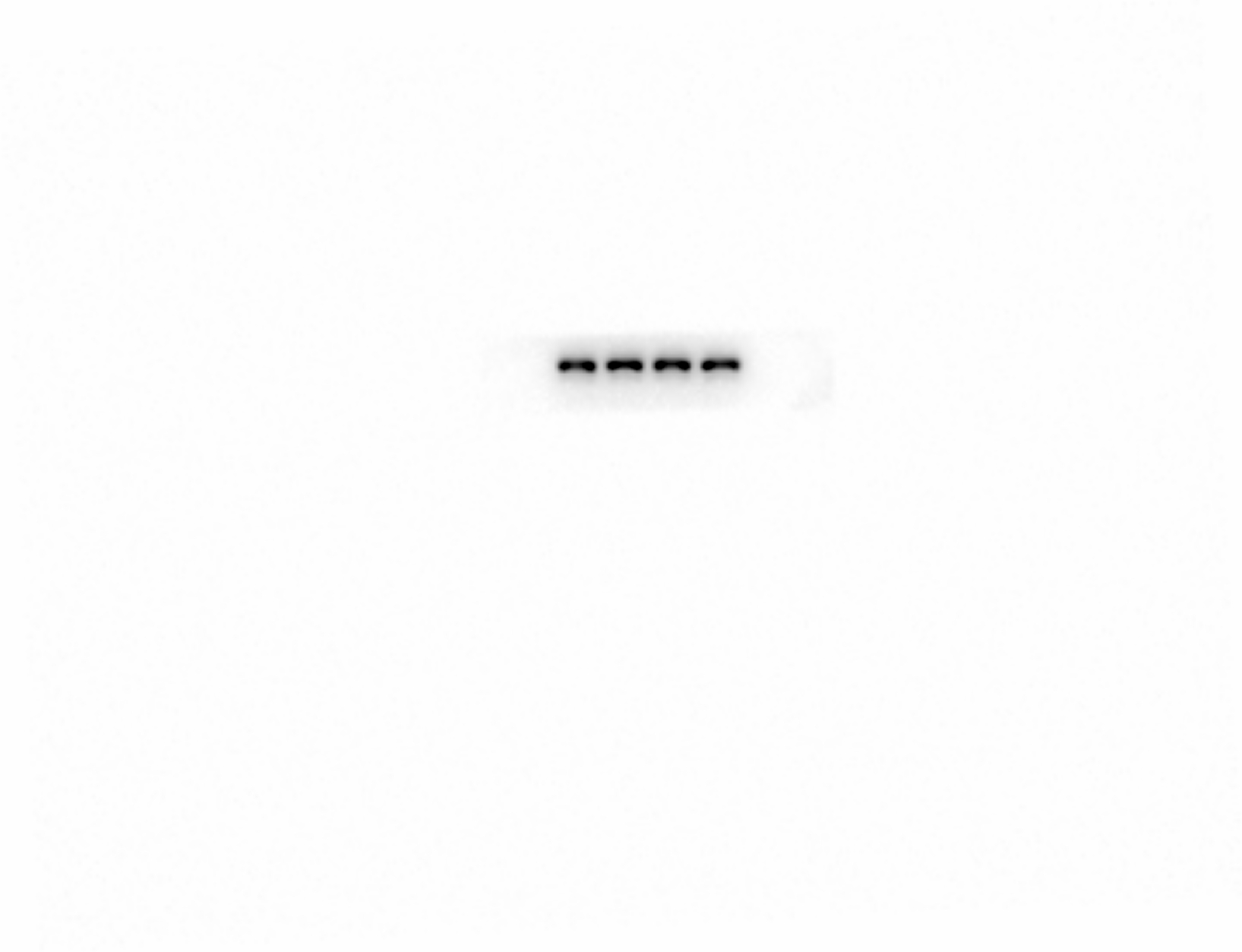


MGC803(OE) p-STAT3(Y705) MGC803(OE) E-cad


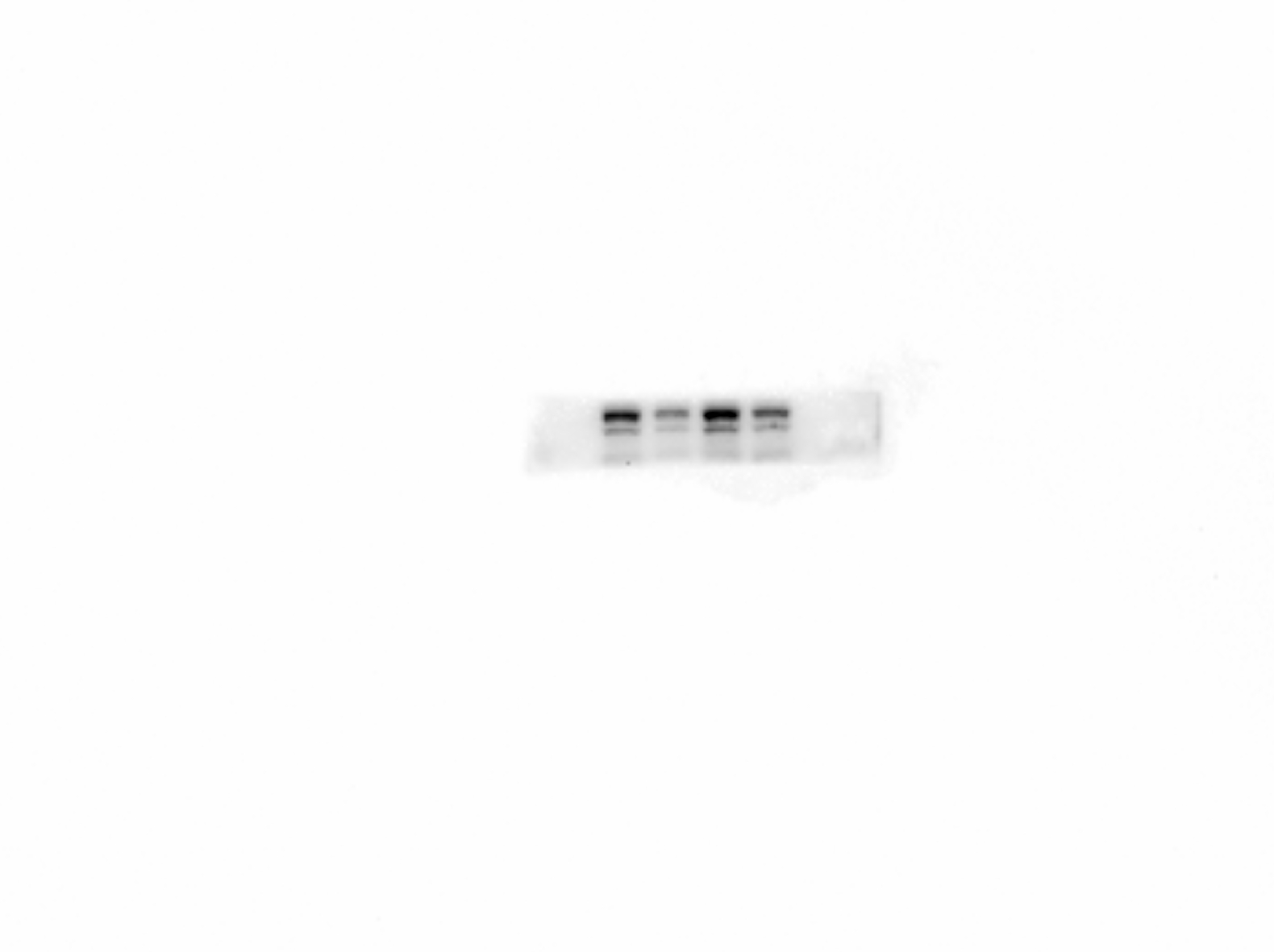

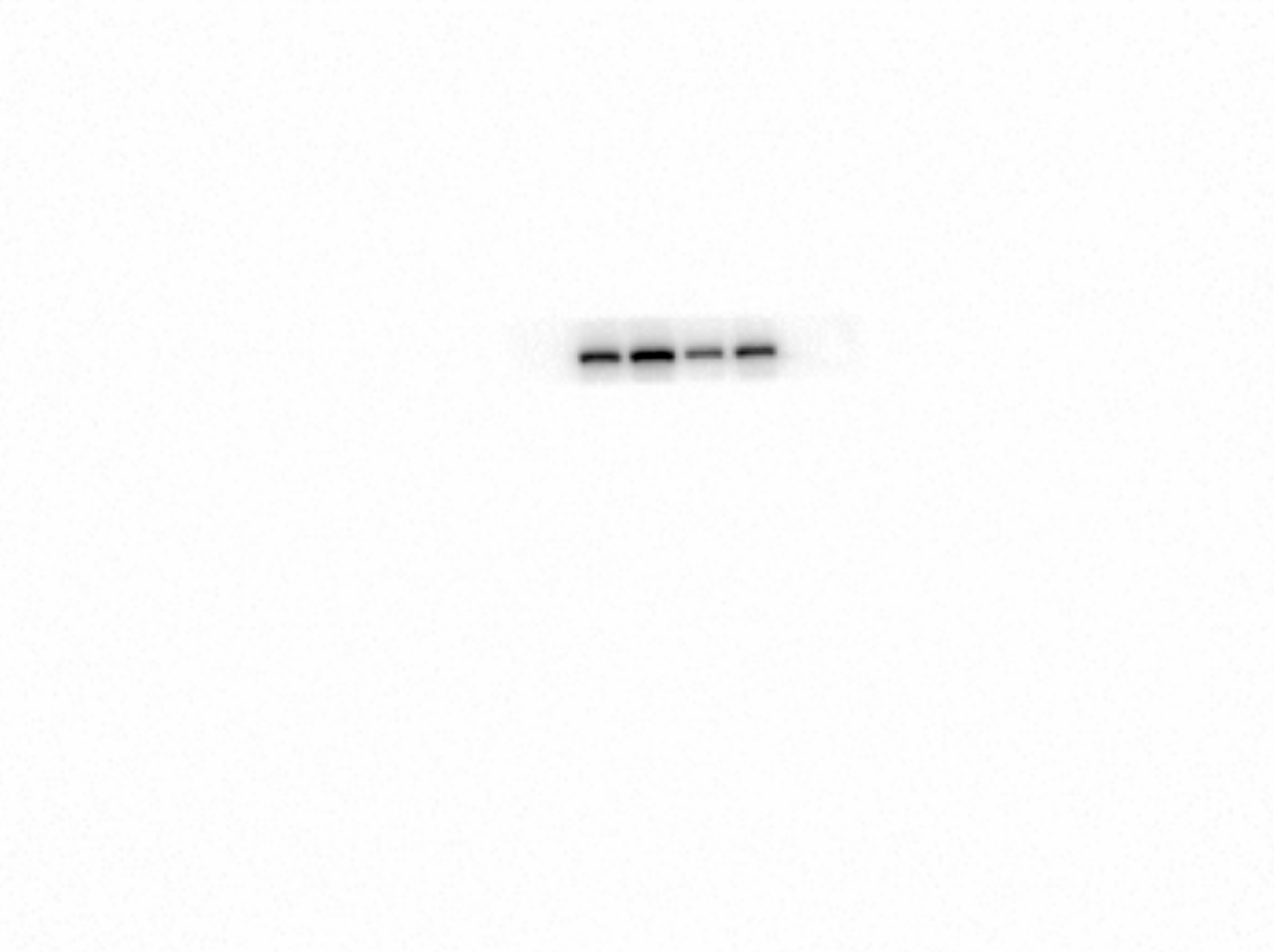


MGC803(OE) N-cad MGC803(OE) Vimentin


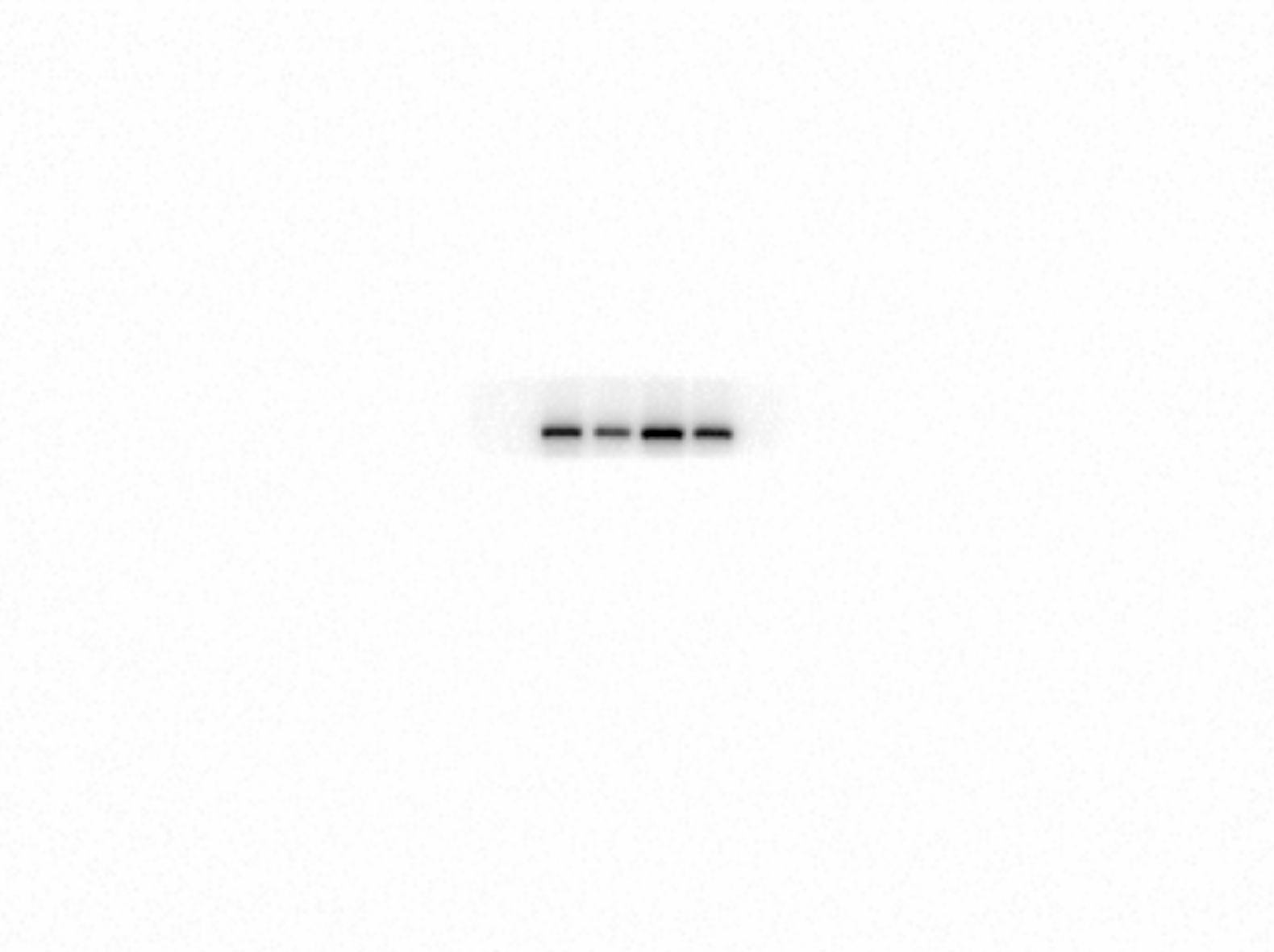

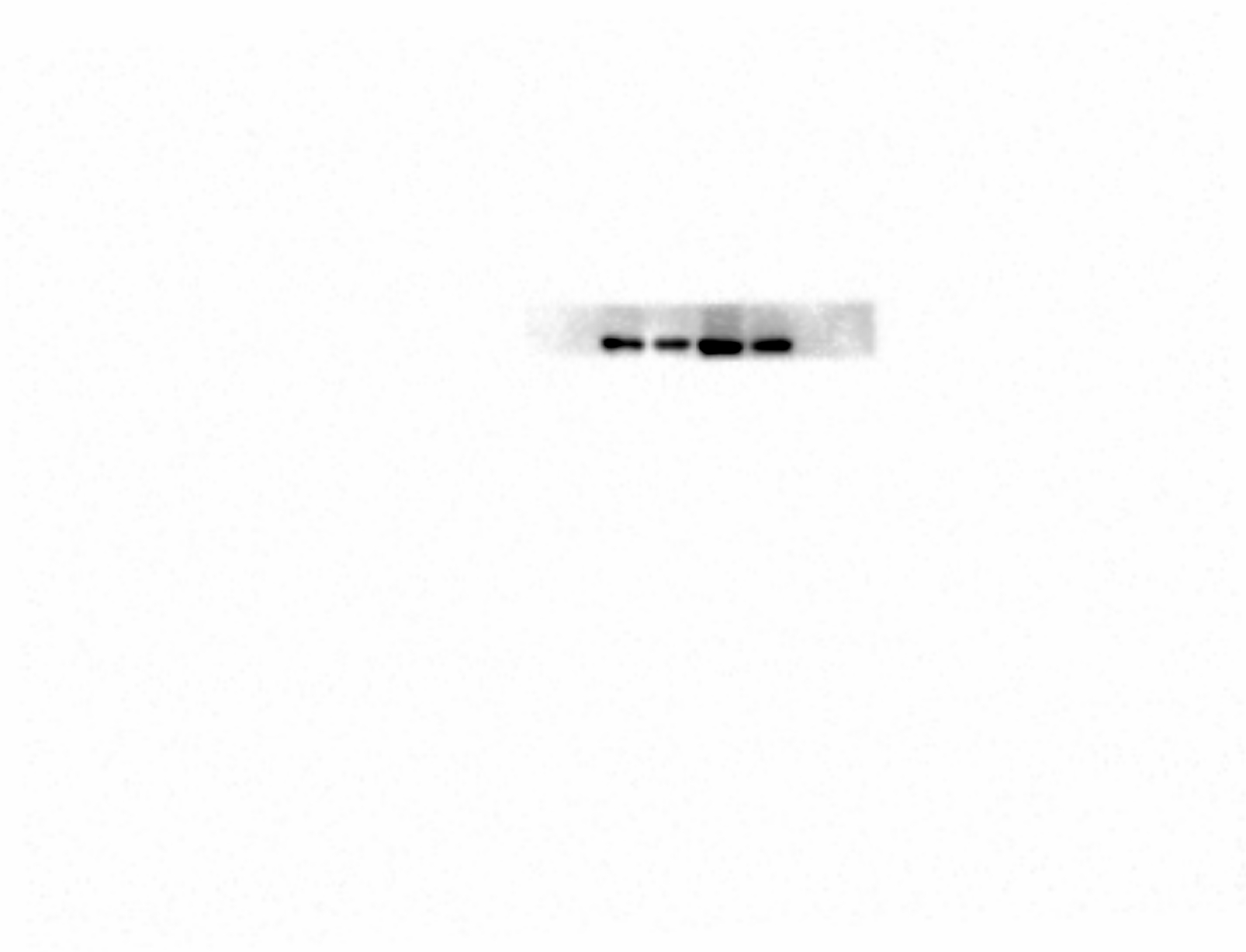


MGC803(OE) GAPDH


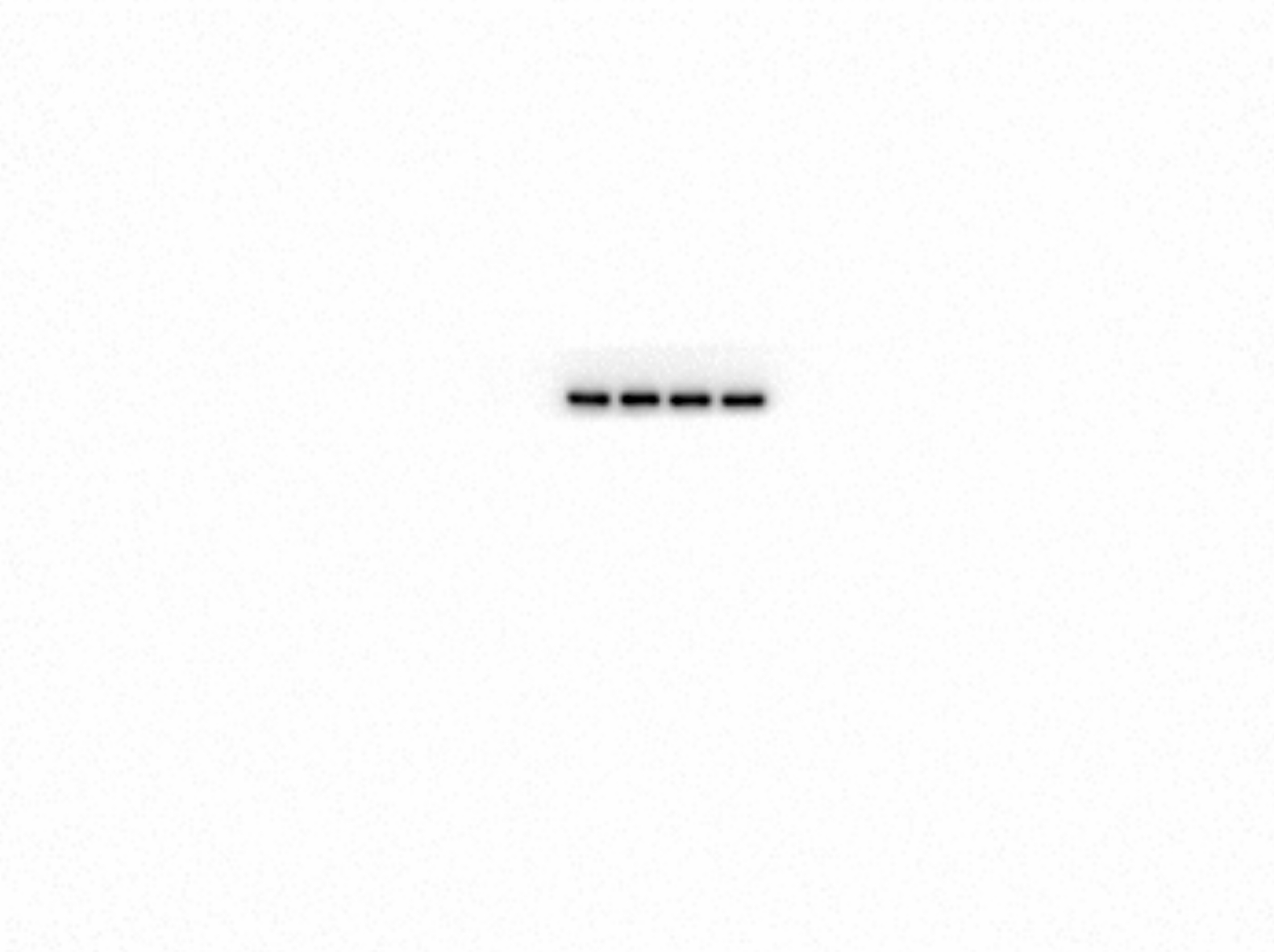


MGC803(sh) Syntenin MGC803(sh) STAT3


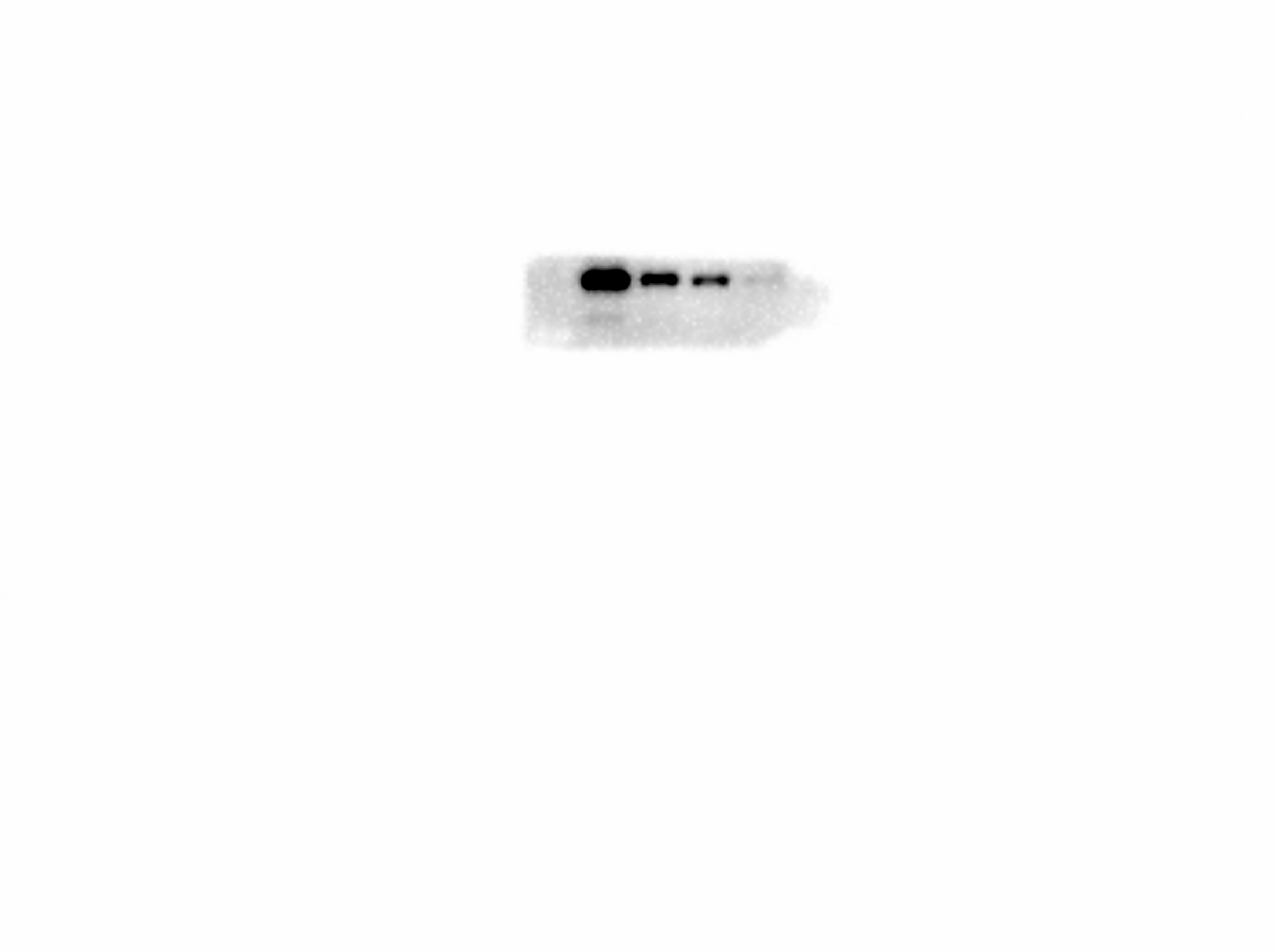

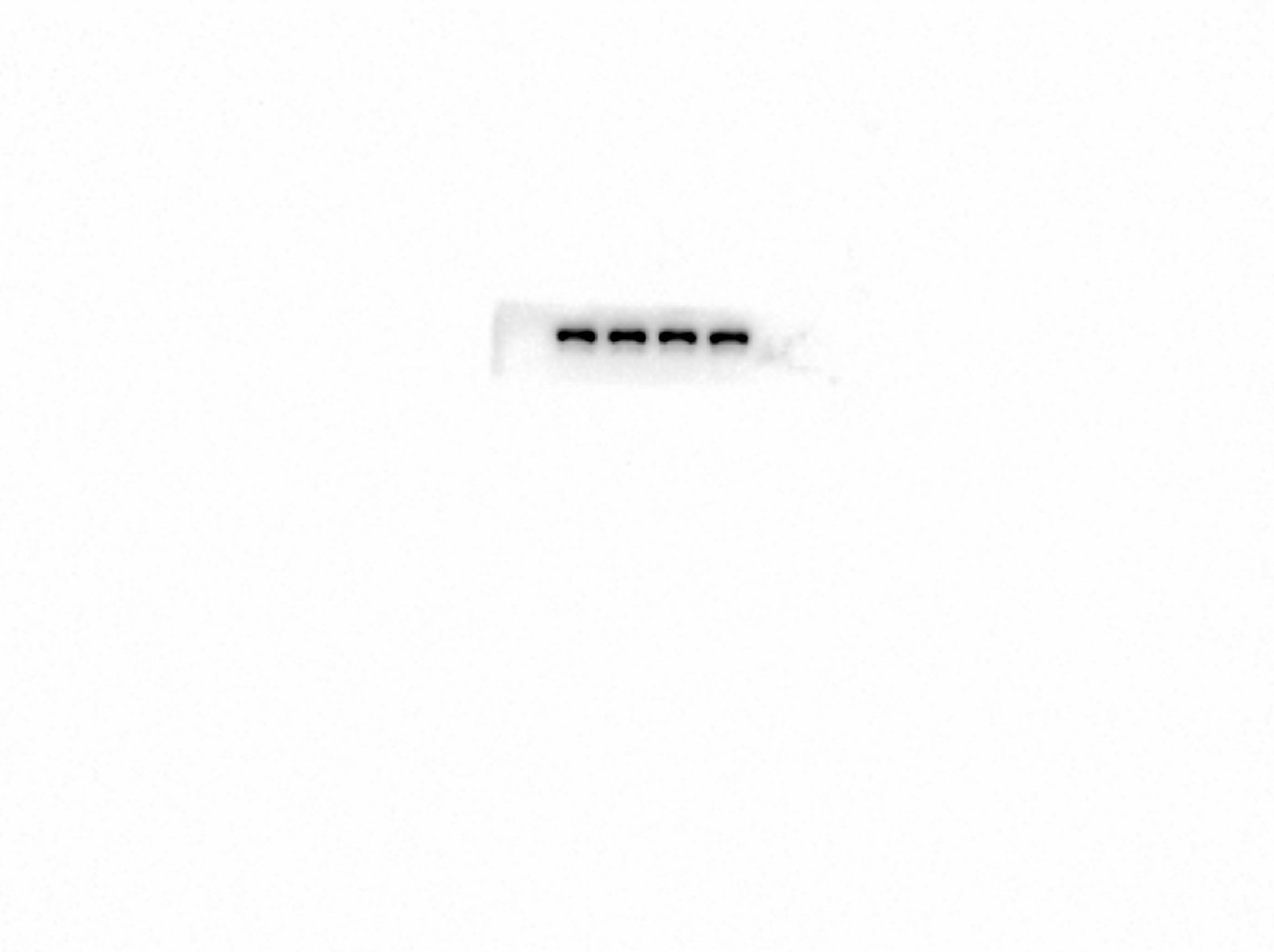


MGC803(sh) p-STAT3(Y705) MGC803(sh) E-cad


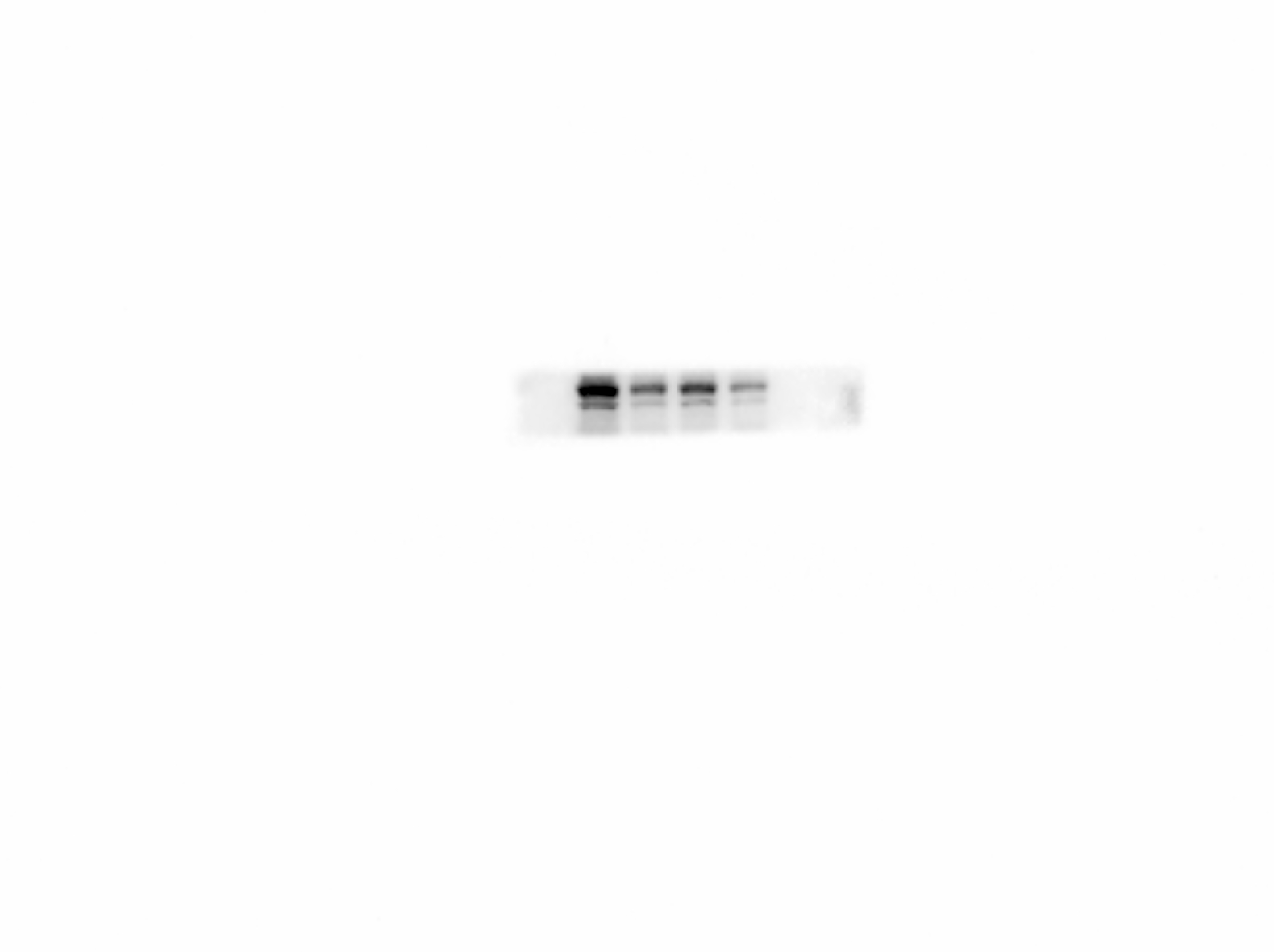

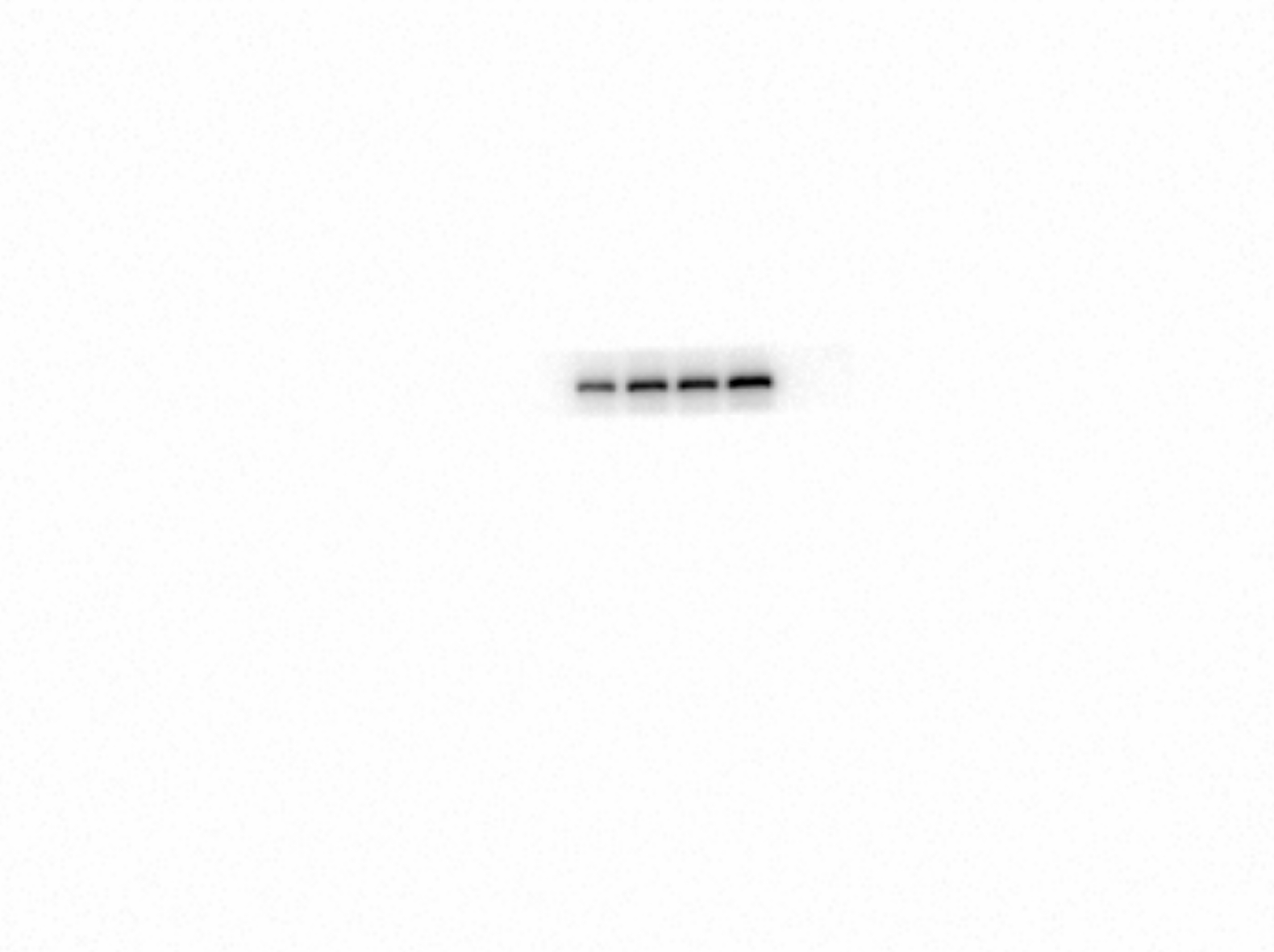


MGC803(sh) N-cad MGC803(sh) Vimentin


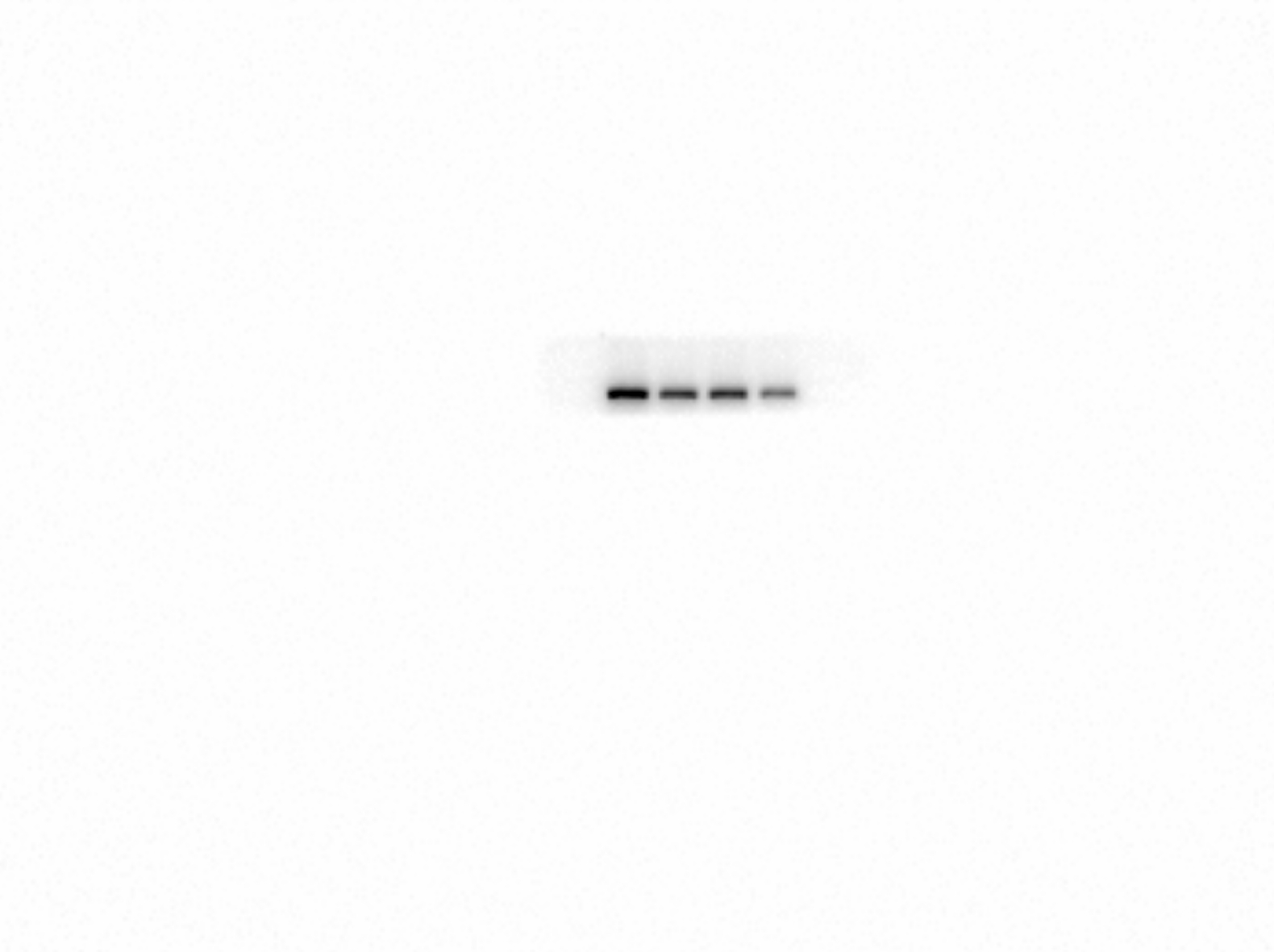

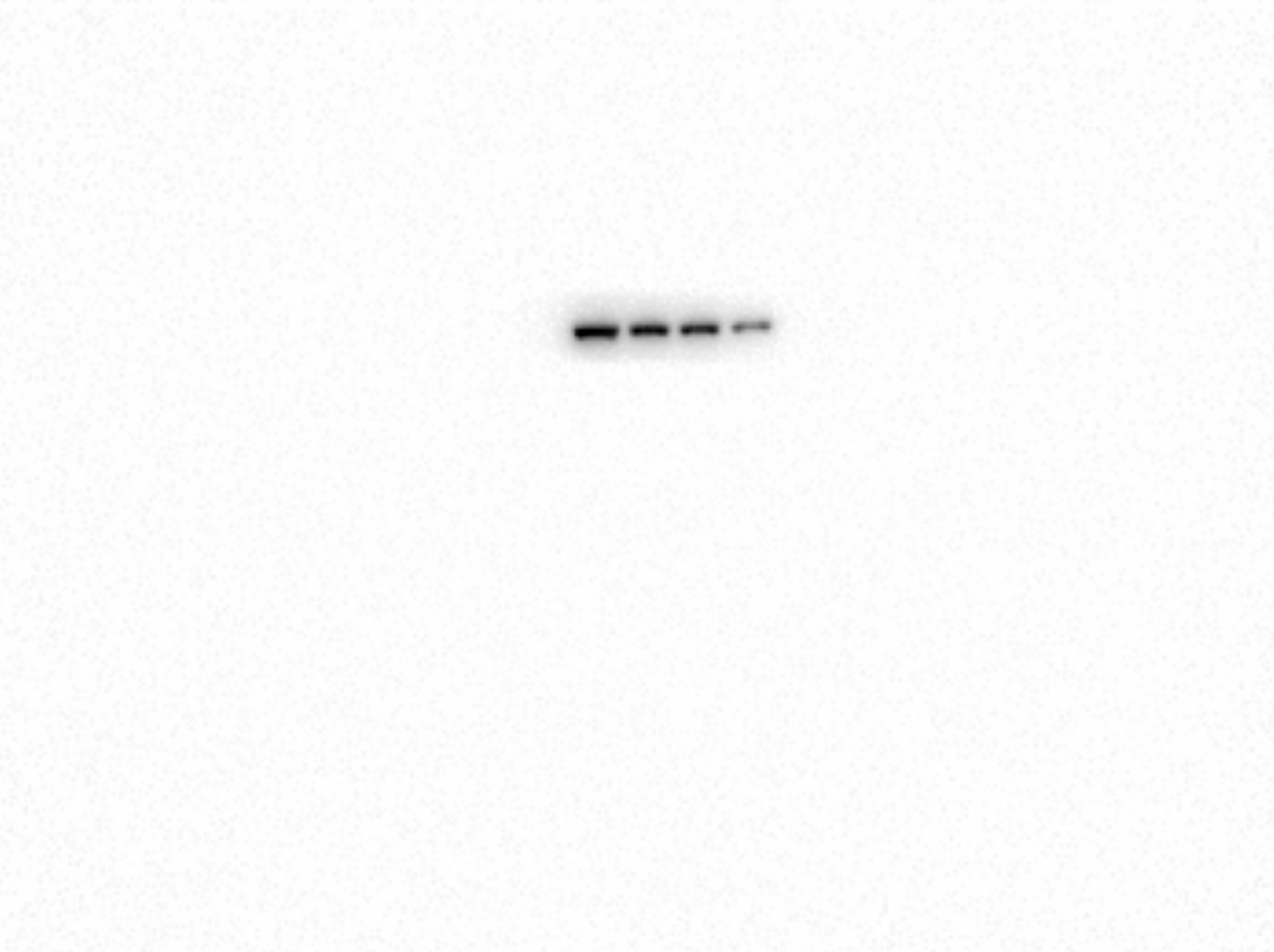


MGC803(sh) GAPDH


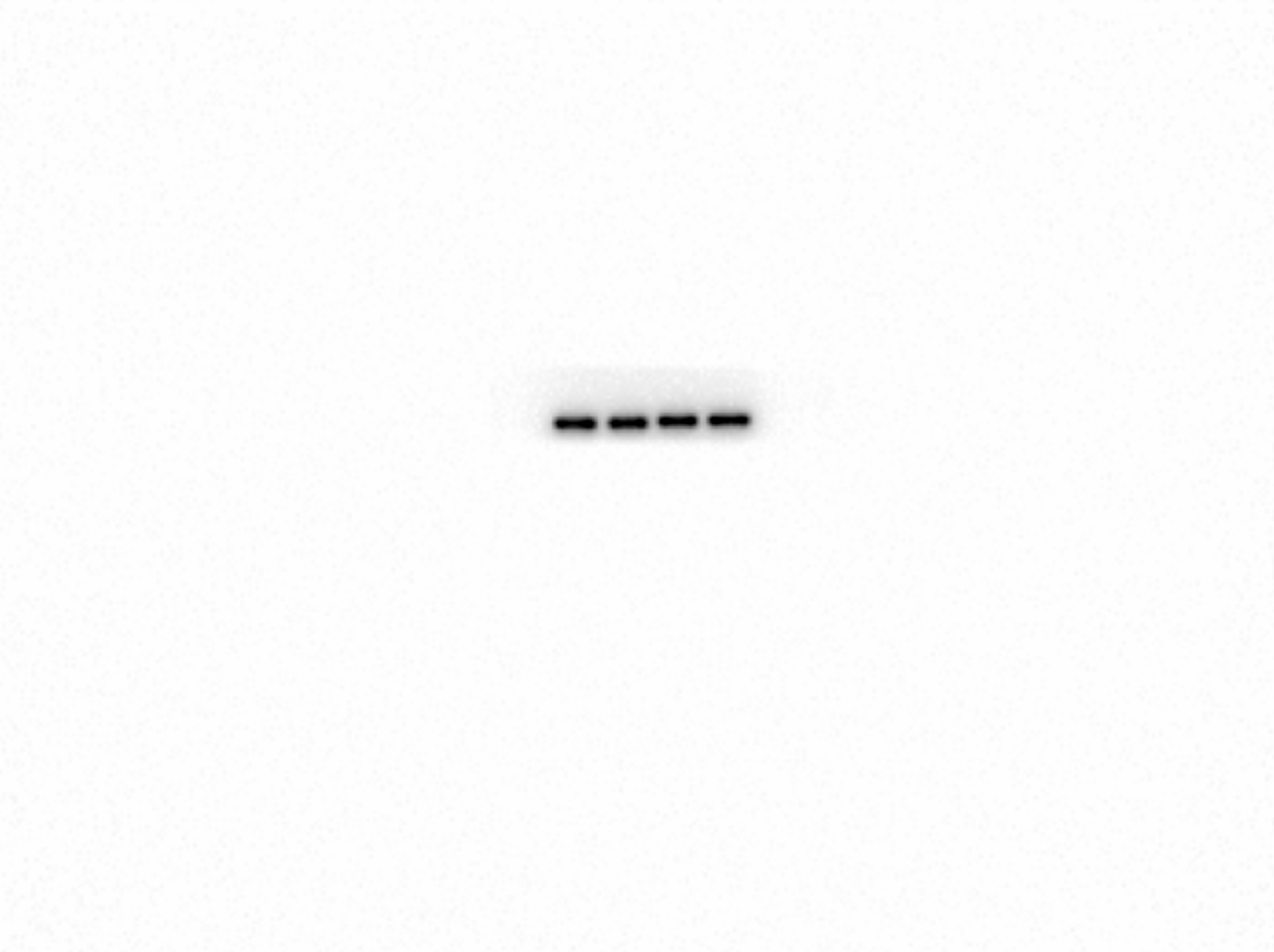


MKN74(OE) Syntenin MKN74(OE) STAT3


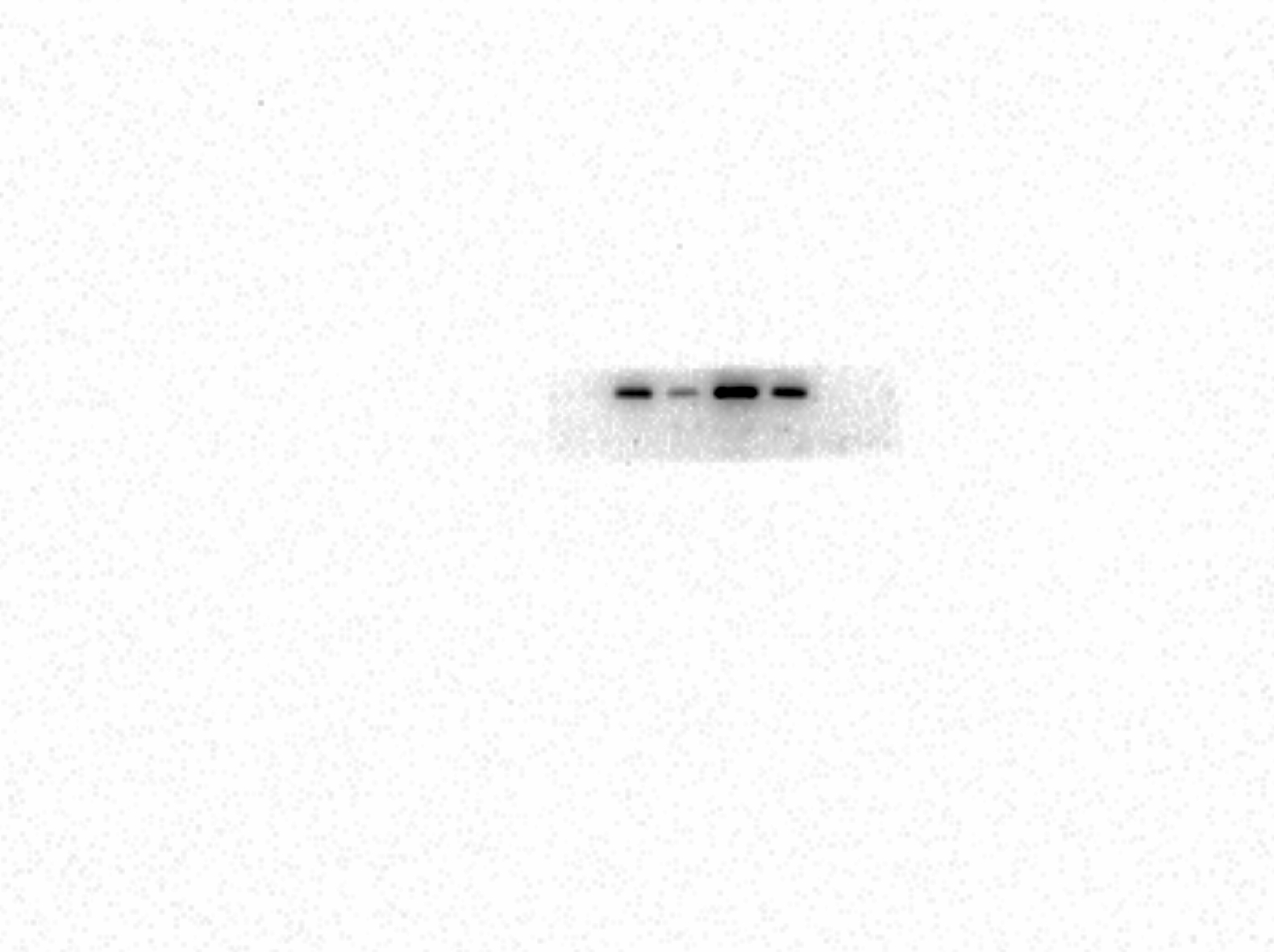

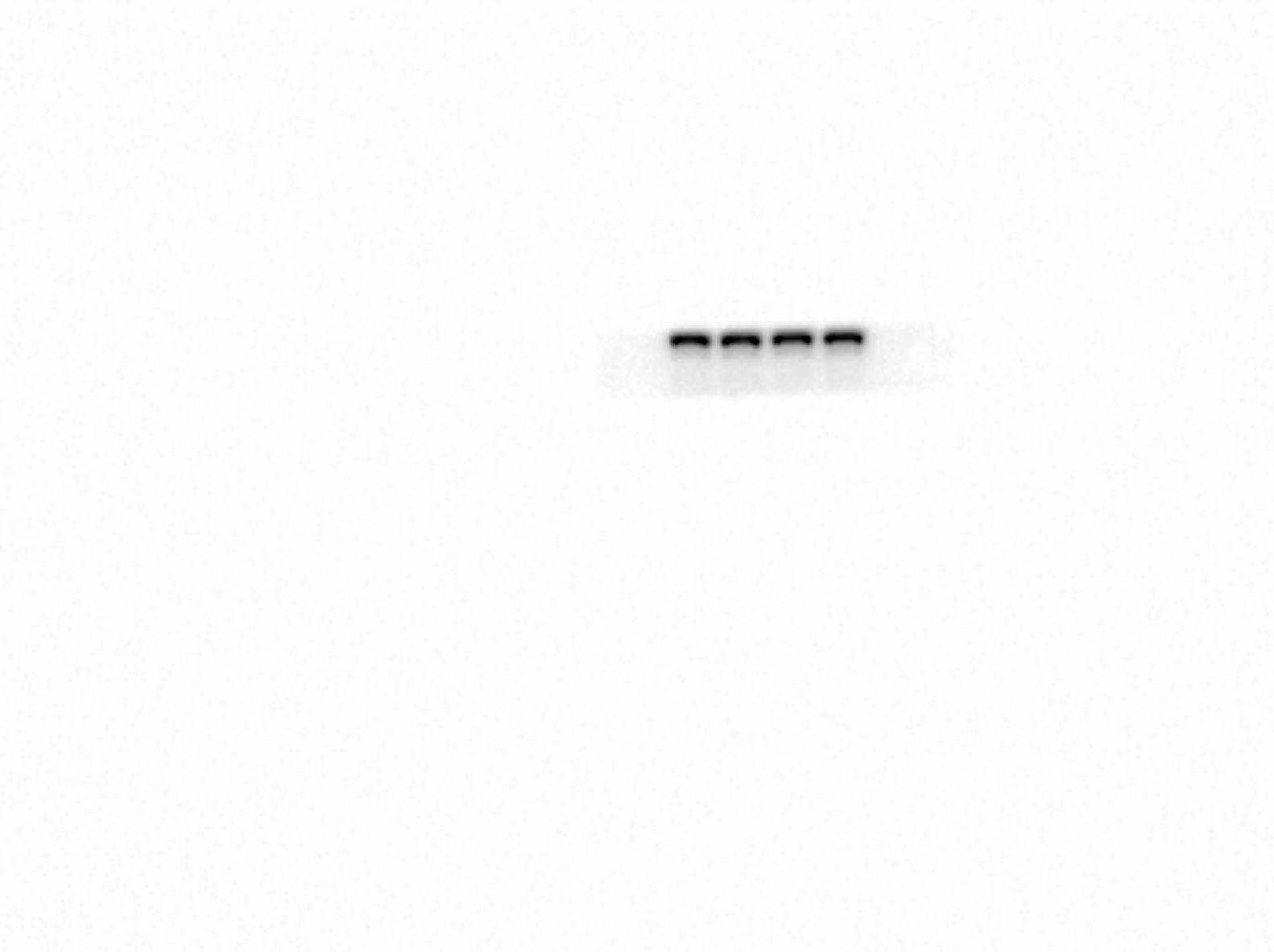


MKN74(OE) p-STAT3(Y705) MKN74(OE) E-cad


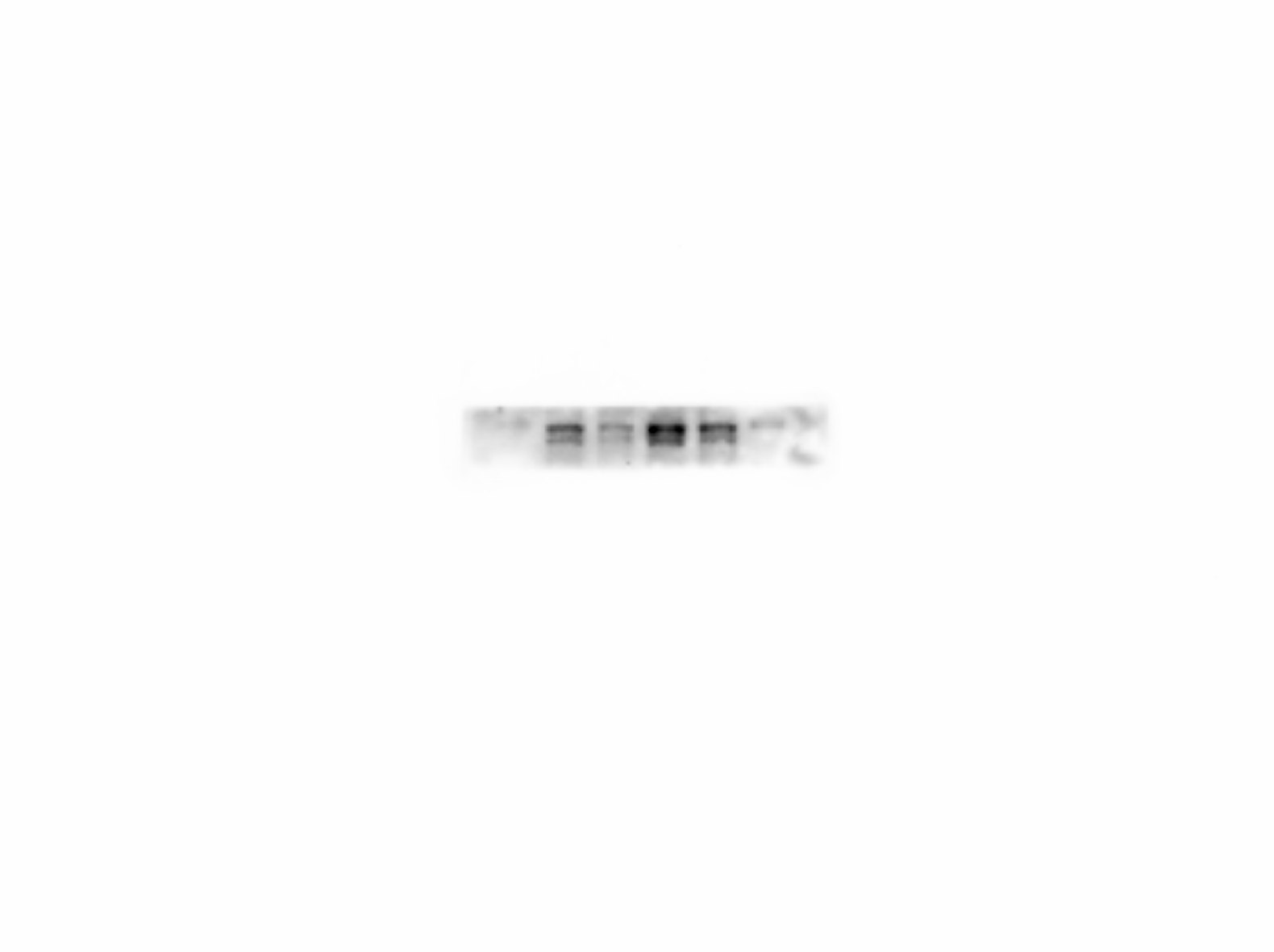

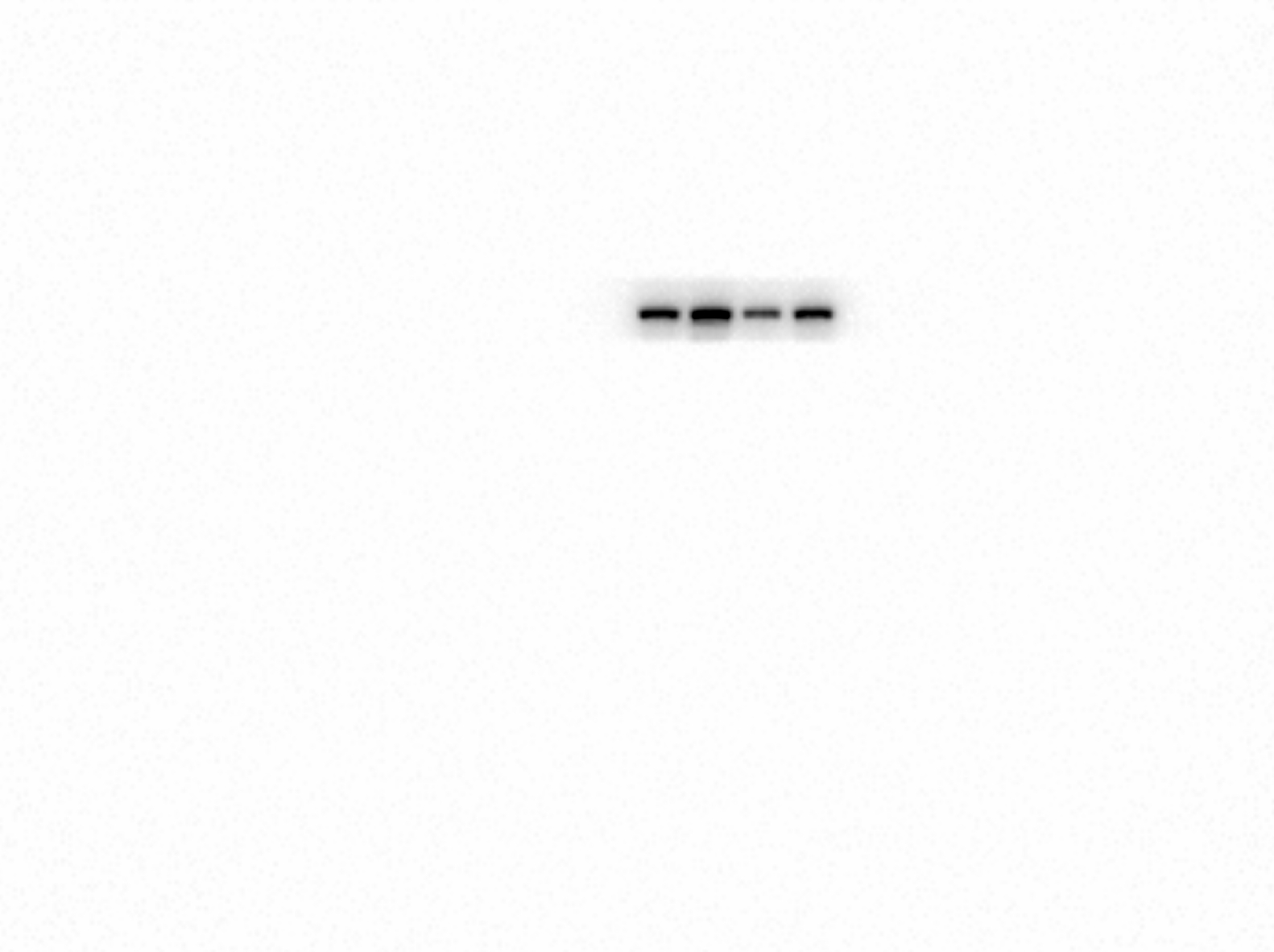


MKN74(OE) N-cad MKN74(OE) Vimentin


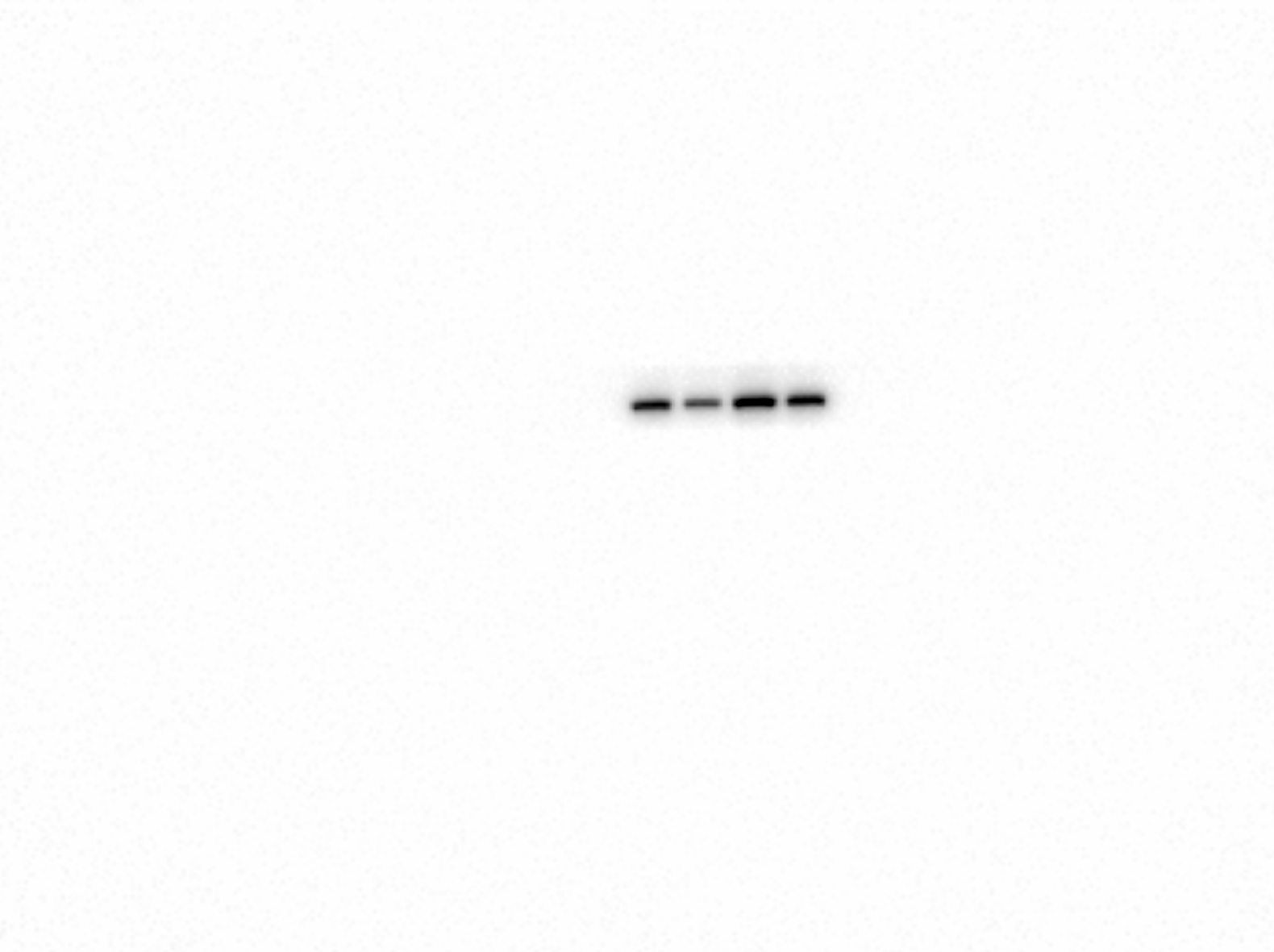

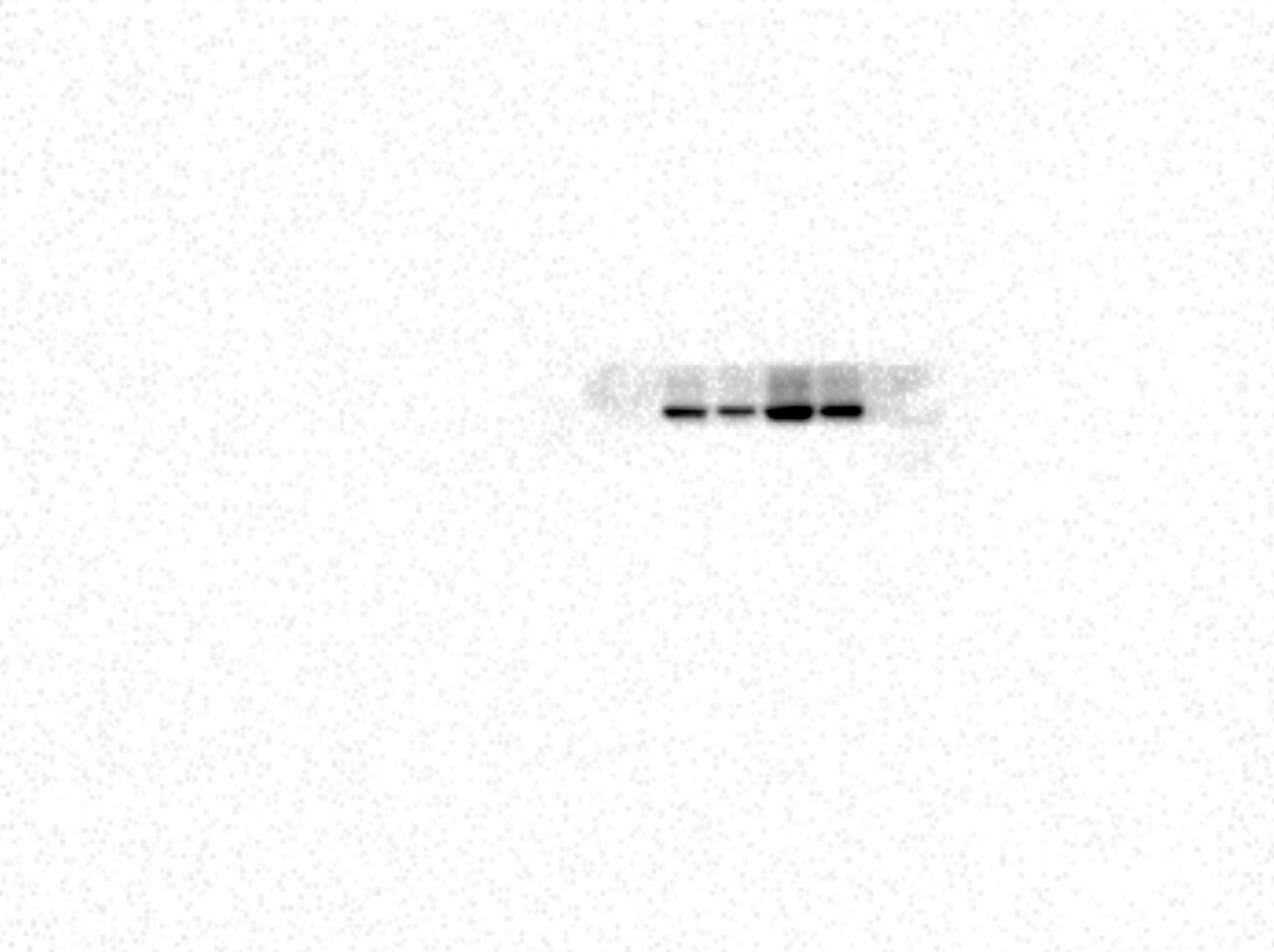


MKN74(OE) GAPDH


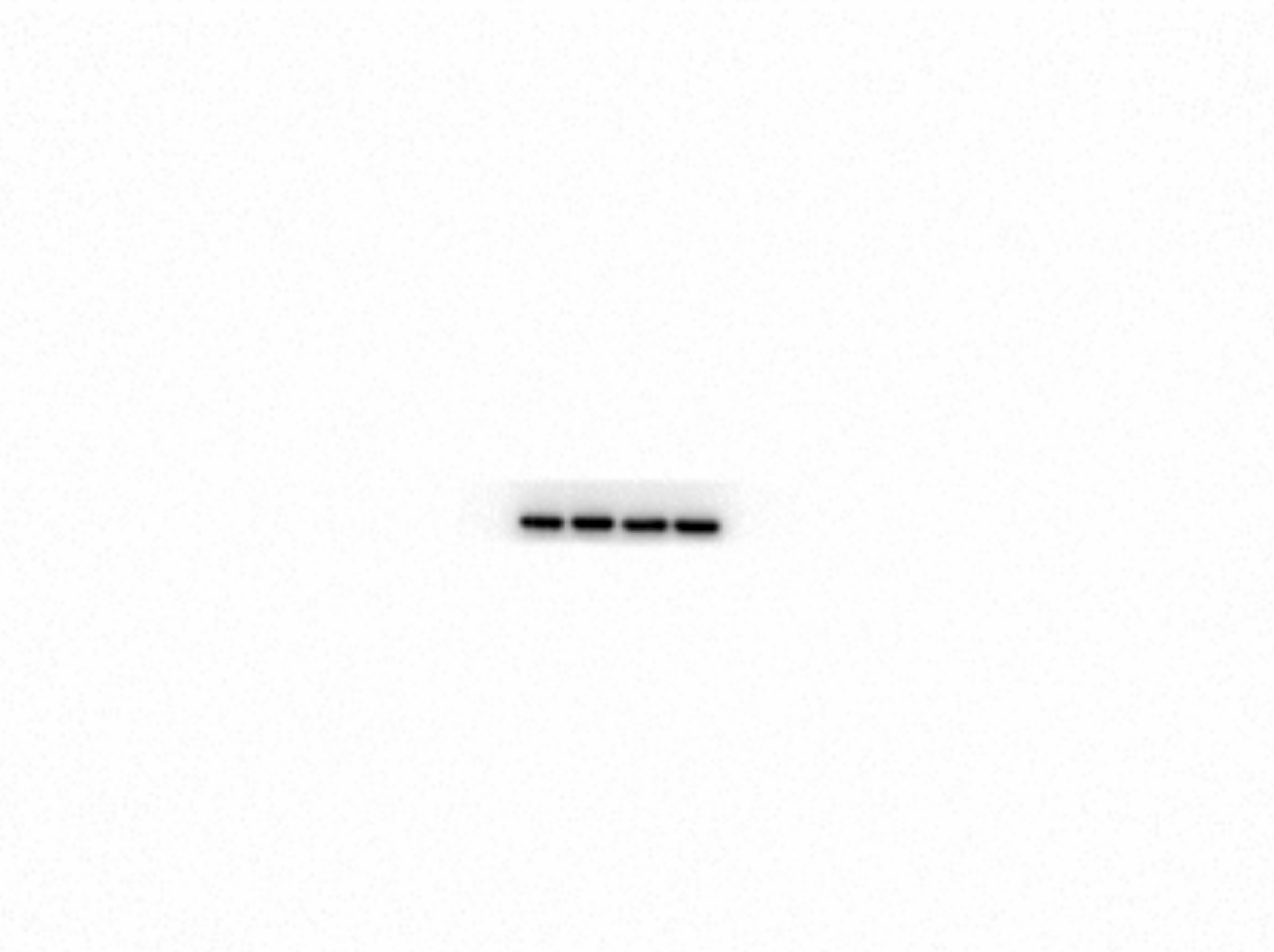


MKN74(sh) Syntenin MKN74(sh) STAT3


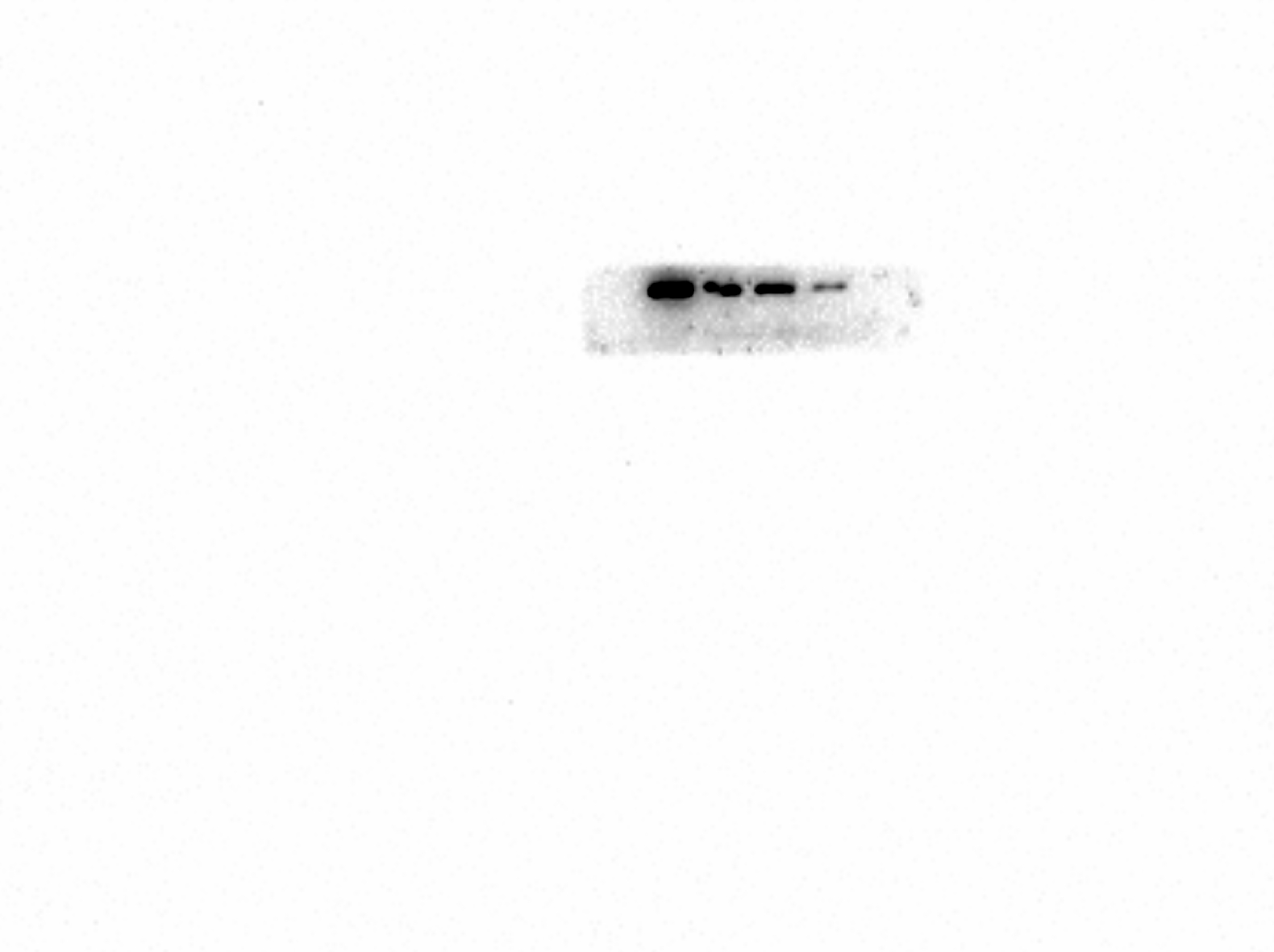

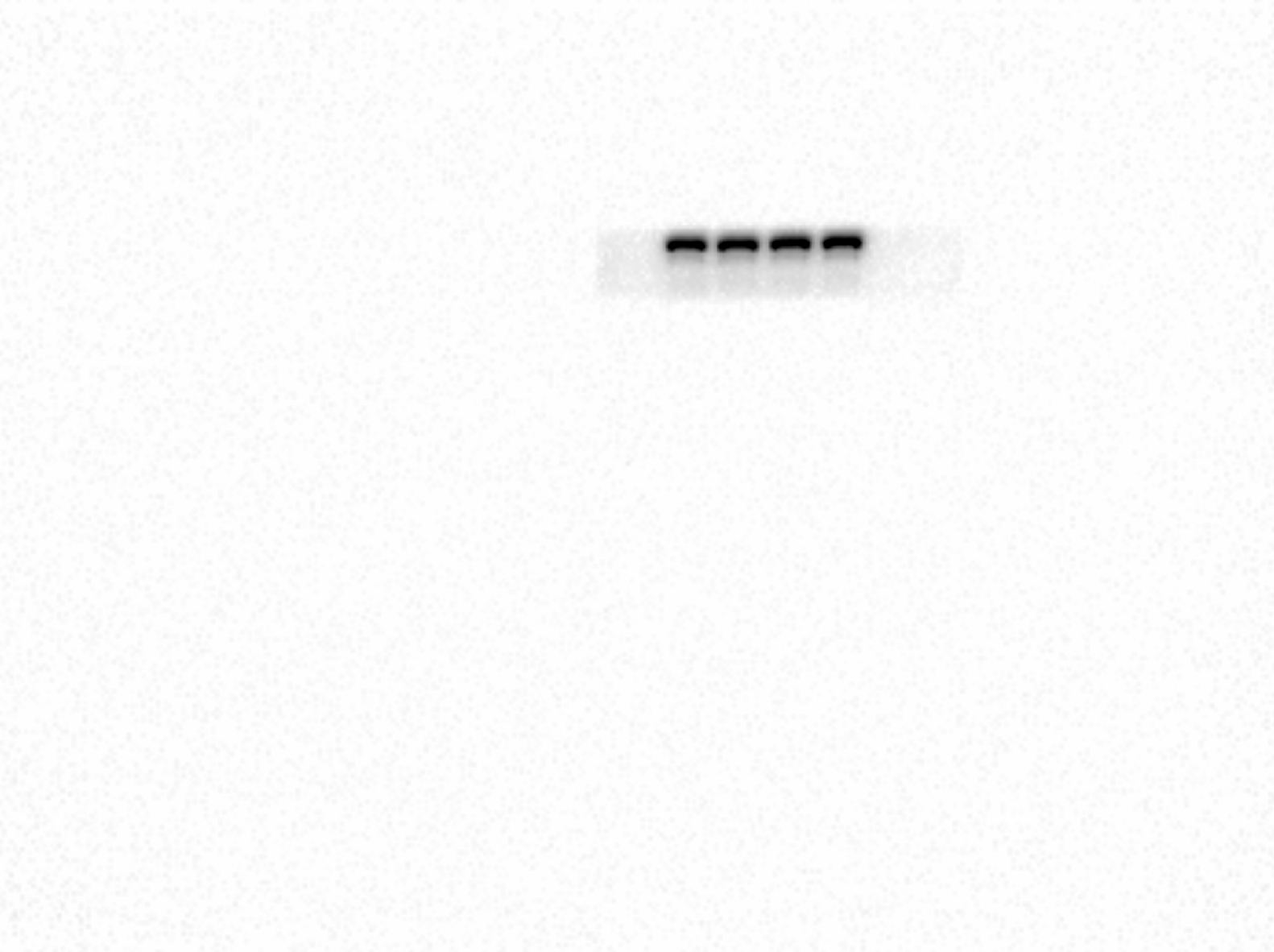


MKN74(sh) p-STAT3(Y705) MKN74(sh) E-cad


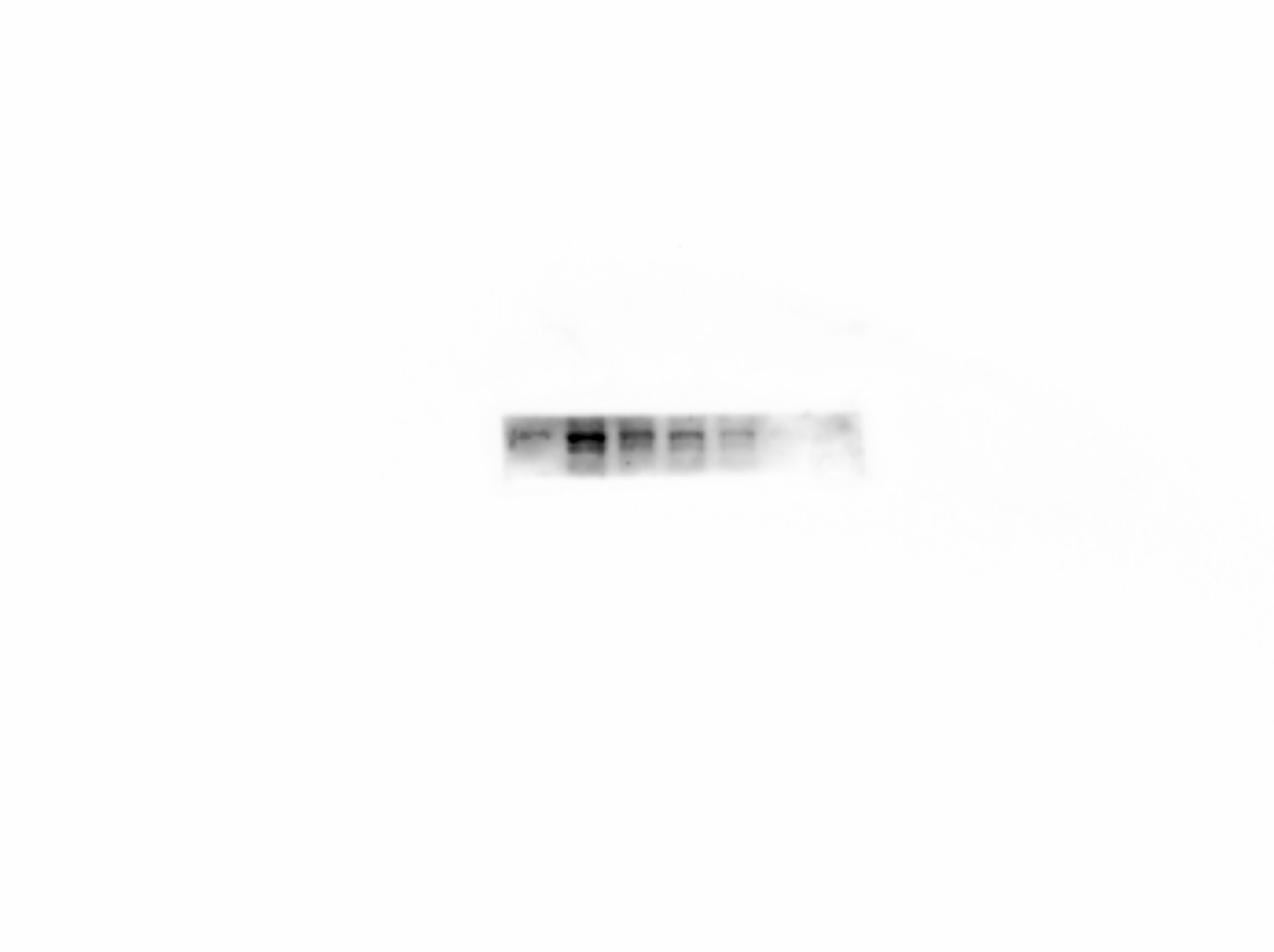

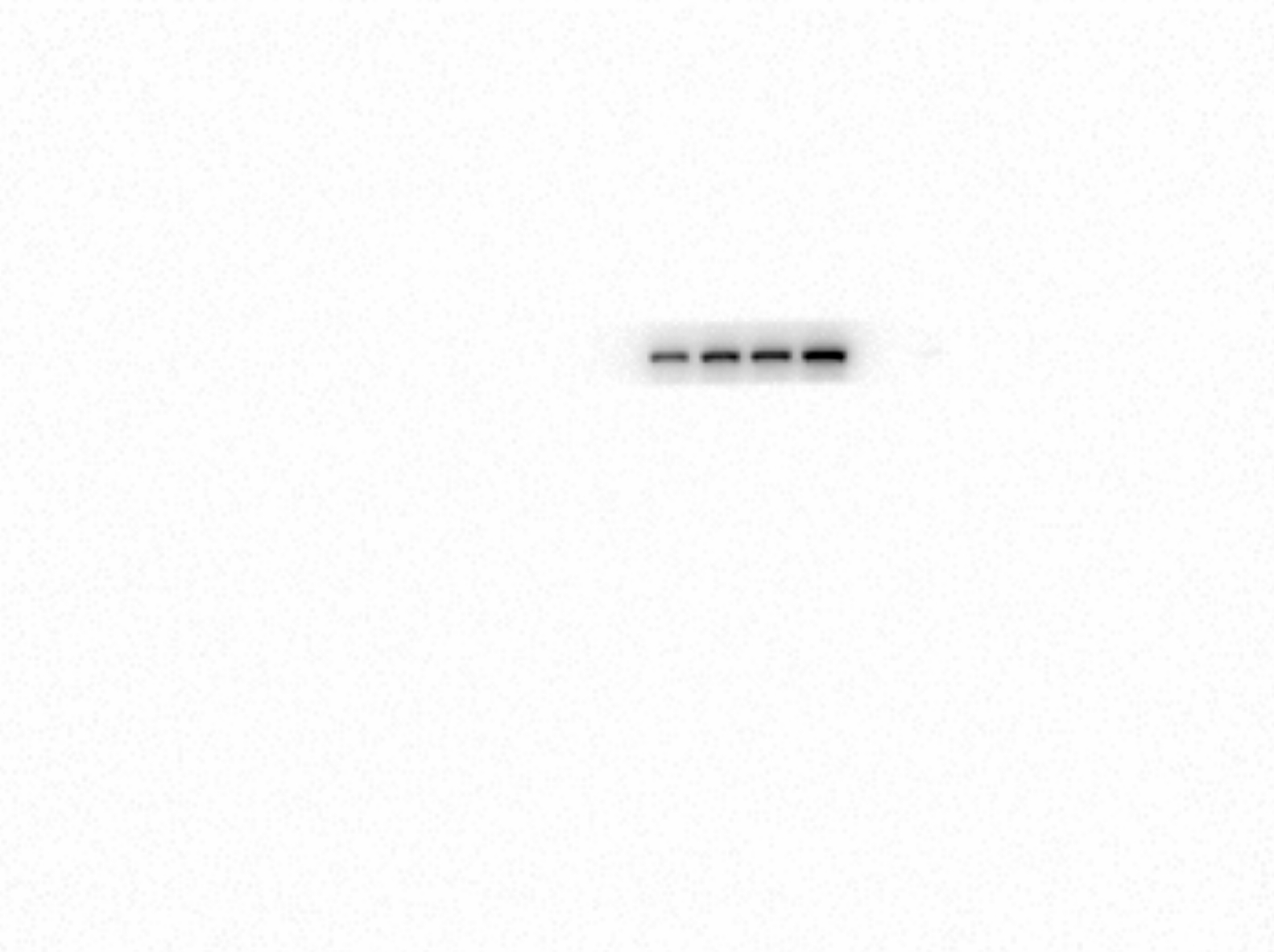


MKN74(sh) N-cad MKN74(sh) Vimentin


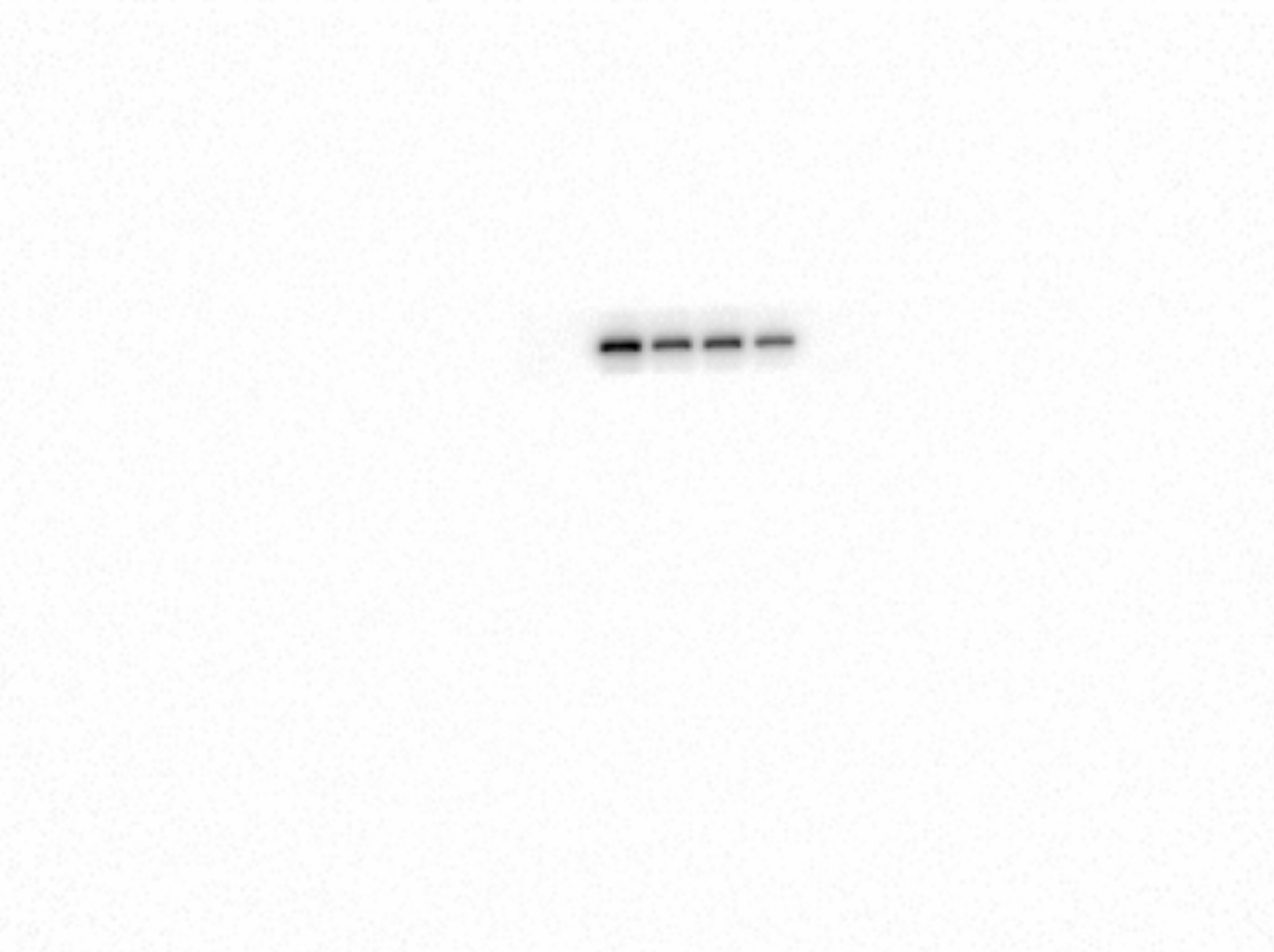

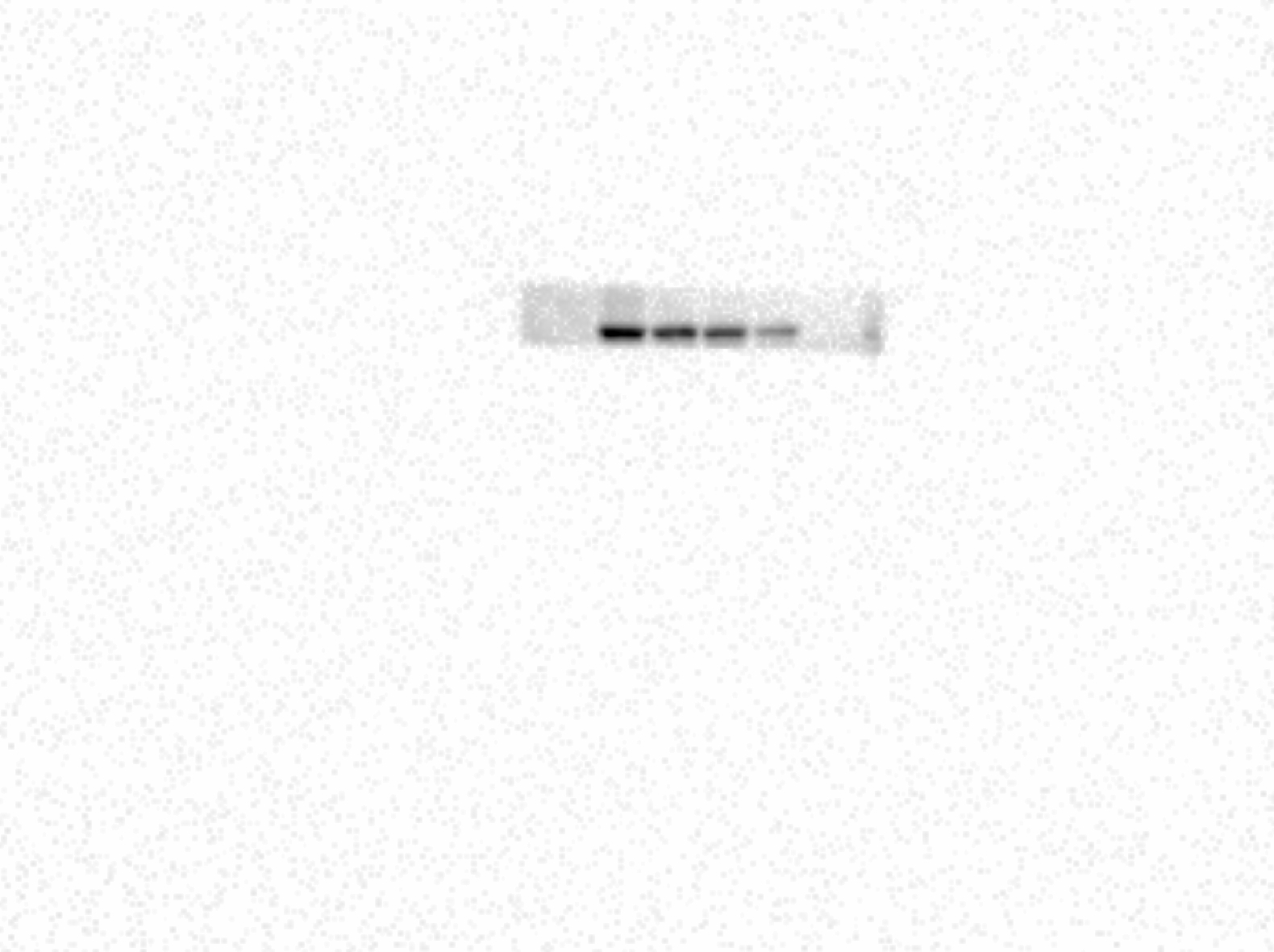


MKN74(sh) GAPDH


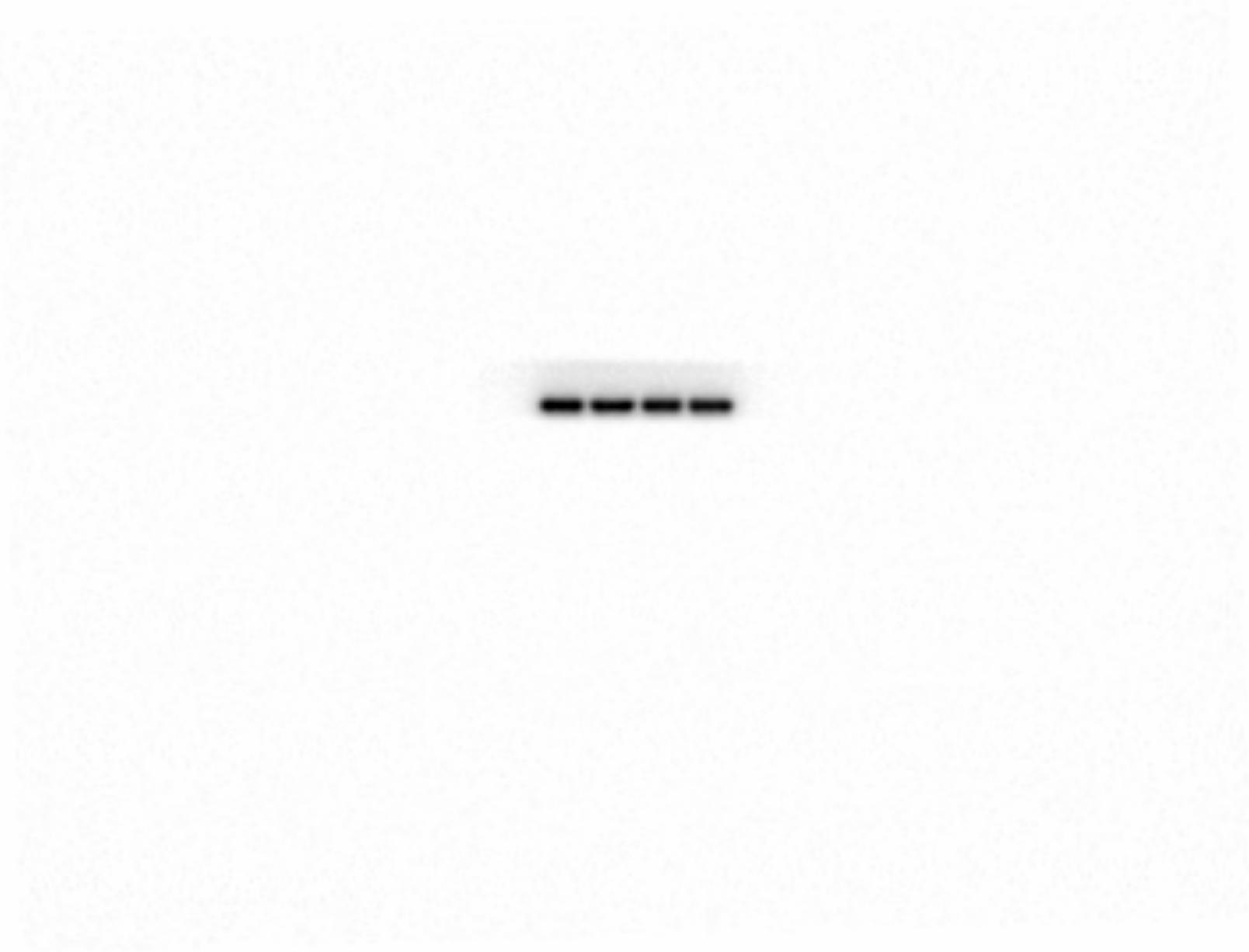

Supplement: Supplementary Materials — Figure S1: BPC chromatogram. Figure S2: mass 219.0286 with 30 ppm mass accuracy. Figure S3: mass 365.0903 with 30 ppm mass accuracy. Figure S4: mass 381.0808 with 30 ppm mass accuracy. Figure S5: mass 248.1147 with 30 ppm mass accuracy. Figure S6: mass 241.1557 with 30 ppm mass accuracy. Figure S7: mass 609.2589 with 30 ppm mass accuracy. Figure S8: mass 369.3534 with 30 ppm mass accuracy. Figure S9: the peripheral blood routine and the liver and kidney function showed no abnormalities. (A) The blood routine of mice in different groups. (B) The liver and kidney function in different groups. Table S1: the LC-MS component analysis of the Huaier n-butanol extract. Table S2: univariate and multivariate Cox analyses of prognostic factors for GC. [file 6065516.f1.doc]
